# The association between body mass index and metabolite response to a liquid mixed meal challenge: a Mendelian randomization study

**DOI:** 10.1016/j.ajcnut.2024.03.009

**Published:** 2024-03-16

**Authors:** David A Hughes, Ruifang Li-Gao, Caroline J Bull, Renée de Mutsert, Frits R Rosendaal, Dennis O Mook-Kanamori, Ko Willems van Dijk, Nicholas J Timpson

**Affiliations:** 1 MRC Integrative Epidemiology Unit, University of Bristol, Bristol, United Kingdom; 2 Population Health Science, Bristol Medical School, University of Bristol, Bristol, United Kingdom; 3 Department of Clinical Epidemiology, Leiden University Medical Center, Leiden, the Netherlands; 4 Department of Public Health and Primary Care, Leiden University Medical Center, Leiden, the Netherlands; 5 Department of Human Genetics, Leiden University Medical Center, Leiden, the Netherlands; 6 Division of Endocrinology, Department of Internal Medicine, Leiden University Medical Center, Leiden, the Netherlands

**Keywords:** metabolomics, lipidomics, obesity, instrumental variable analysis, causal inference

## Abstract

**Background:**

Metabolite abundance is a dynamic trait that varies in response to environmental stimuli and phenotypic traits, such as food consumption and body mass index (BMI, kg/m^2^).

**Objectives:**

In this study, we used the Netherlands Epidemiology of Obesity (NEO) study data to identify observational and causal associations between BMI and metabolite response to a liquid meal.

**Methods:**

A liquid meal challenge was performed, and Nightingale Health metabolite profiles were collected in 5744 NEO participants. Observational and one-sample Mendelian randomization (MR) analysis were conducted to estimate the effect of BMI on metabolites (*n* = 229) in the fasting, postprandial, and response (or change in abundance) states.

**Results:**

We observed 473 associations with BMI (175 fasting, 188 postprandial, and 110 response) in observational analyses. In MR analyses, we observed 20 metabolite traits (5 fasting, 12 postprandial, and 3 response) to be associated with BMI. MR associations included the glucogenic amino acid alanine, which was inversely associated with BMI in the response state (β: −0.081; SE: 0.023; *P* = 5.91 × 10^−4^), suggesting that as alanine increased in postprandial abundance, that increase was attenuated with increasing BMI.

**Conclusions:**

Overall, this study showed that MR estimates were strongly correlated with observational effect estimates, suggesting that the broad associations seen between BMI and metabolite variation has a causal underpinning. Specific effects in previously unassessed postprandial and response states are detected, and these may likely mark novel life course risk exposures driven by regular nutrition.

## Introduction

The excess accumulation of body fat and obesity are an established global health burden, the prevalence of which is increasing. BMI, or the ratio of one’s weight and height squared, is a common metric for measuring excess weight or fat and BMI thresholds ≥25 and 30 are used to designate the conditions of overweight and obesity, respectively [[1], [2], [3], [4], [5], [6]]. Increases in BMI have been associated with decreased life expectancy [[7], [8], [9], [10]], some cancers [[11], [12], [13]] and cardiometabolic diseases including cardiovascular disease, and type 2 diabetes [14].

Despite the scale of the BMI-related health burden, there remains a need to elucidate how and by what intermediate physiological traits excess body fat increases disease risk. Possible intermediates are metabolites and lipoproteins, some of which—such as low density lipoprotein (LDL) or its’ crucial protein apolipoprotein B—have been previously associated with both BMI and disease risk [[15], [16], [17], [18], [19], [20], [21], [22], [23]]. Previous cross-sectional, Mendelian randomization (MR), recall-by-genotype, and randomized controlled trials have established both observational and causal associations between BMI and fasted metabolite and protein abundances [15,[24], [25], [26]]. Such studies have informed and motivated downstream studies aimed at elucidating the causal effects of BMI-driven metabolite variation on disease outcomes, such as colorectal cancer [27,28].

However, with most individuals spending much of their waking hours in a nonfasted or postprandial state, it is important to understand both the variation in postprandial metabolite abundance and the association and influence BMI may have on dynamic ranges in metabolomic response. Response, defined as the change in metabolite abundance between fasted and postprandial states, may prove to be an emergent trait in disease risk research. Given that response is dependent on the starting, fasted, abundance level—which itself may be influenced by BMI—it becomes an instructive trait to evaluate interplays among BMI, metabolite variation, and disease risk. For example, we predict that some BMI-driven metabolite traits may have neared or reached a physiologic plateau in the fasted state, such that any response due to a meal has a limited range of change. Resultantly, response may prove to be a more powerful phenotype to identify molecular traits influenced by BMI than single time points. Previous work has provided evidence that this dynamic state is different and potentially informative versus classically measured fasting levels. For example, a recent genome-wide association analysis identified genetic variants uniquely associated with metabolite response traits that did not share associations with fasting or postprandial abundances [29]. Moreover, estimates of genotype heritability for some response traits, such as the (branched-chain) amino acids, glucose and extremely large very low density lipoprotein (VLDL), are as large or larger than those observed for fasting and postprandial estimates [29]. Taken together, results suggest that such state-specific traits are likely to be qualitatively different and may prove informative in explaining the etiology of disease.

**FIGURE 1:**
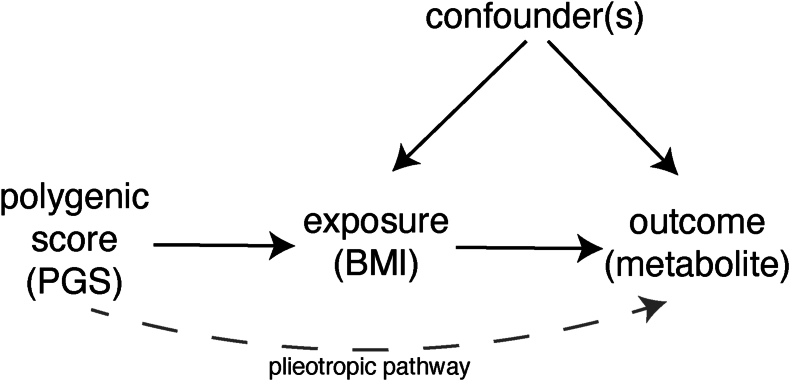
A schematic or directed acyclic graph of the Mendelian randomization (MR) framework. The MR framework in this study assumed that *1*) the instrument is robustly associated with the outcome, *2*) the instrument is not associated with variables that are associated to the outcome, and *3*) the instrument acts on the outcome only through the exposure and not through a pleiotropic pathway.

We combined data measuring the metabolic response to a liquid mixed meal and measures of the exposure, BMI, in observational and MR frameworks. MR is a framework using instrumental variable (IV) analysis to deploy genetic variants as exposure proxies in efforts to obtain evidence of causal relationships (Figure 1) [30,31]. These analyses, undertaken in the Netherlands Epidemiology of Obesity (NEO) study, aimed to estimate the relationship between variation in BMI and metabolite abundance in both fasted and postprandial states and to assess metabolite response—or change in abundance, between fasted and postprandial states.

## Methods

### Study sample

The sample population was 6671 participants of the NEO study, a prospective cohort study [32]. The NEO study was conducted at the Leiden University Medical Center (LUMC) and approved by the LUMC Medical Ethical Committee. All participants gave written informed consent. Recruitment was conducted between September 2008 and September 2012 by invitation, and sample size is a product of population response.

The NEO sample population is derived from 2 populations. First, all individuals between the ages of 45 and 65 y, living in the greater Leiden area, with a self-reported BMI of 27 or higher were invited to participate. Second, all individuals in the municipality of Leiderdorp of the same age range, regardless of BMI (Supplemental Figure 1) were invited to participate. We will refer to these 2 sample populations as “Leiden” and “Leiderdorp,” respectively, and the entire study sample as “NEO.” In this study, our study sample population had a size of 5744 defined by those participants with genotype data, metabolite data, and other phenotype or covariable data available. Further details of the NEO study and study design are previously published [32].

### Meal challenge

An overnight fasting was requested of all participants who subsequently visited the LUMC NEO study center and given a food challenge [29]. During the visit, first, an initial baseline, fasted blood sample was drawn. Second, within 5 min of the initial blood draw, participants were given a liquid mixed meal. The meal was 400 mL in volume with 600 kcal of energy, of which 16% of energy was derived from protein, 50% from carbohydrates, and 34% was from fat. Third, after completion of the food challenge, a blood sample was drawn at 30 min and again at 150 min. The NEO meal challenge occurred between the months of September 2008 and September 2012, with participants from Leiden undergoing sampling throughout this period. The participants from Leiderdorp were underwent sampling nonexclusively during the months of June 2011 and September 2012.

The meal challenge sampling of Leiderdorp and Leiden population participants was structured by sampling date, and as a product, BMI was structured by sampling date. Specifically, BMI was associated with the date of measurement (univariable linear model *F* test *P* value = 4.7 × 10^−229^), explaining 20.3% of the total variation in BMI (Supplemental Figure 2). Despite this, the BMI polygenic score (BMI-PGS) had a smaller proportion (2.0%) of its variation explained by sampling date (univariate linear model *F* test *P* value = 3.9 × 10^−7^). As a product of this structure, sampling date was included as a covariate in all observational and MR analyses.

### Metabolite data

NEO participants had plasma-derived metabolite profiles measured using the Nightingale Health (Helsinki, Finland) ^1^H nuclear magnetic resonance platform [33]. For each participant, both their fasting and 150-min postprandial samples were assayed. We note here that metabolites are commonly defined as biological molecules of <1.5 kDa in size and that many of the molecules (lipids) assayed in this study are larger than this threshold. For simplicity, we will refer to them all as metabolites. At the time of sampling, this metabolomics platform provided 229 measurements for 149 metabolites, including 80 derived ratio measurements (Supplemental Table 1) from 14 substance classes: amino acids (*n* = 8), apolipoproteins (*n* = 3), cholesterol (*n* = 9), fatty acids (*n* = 11), fatty acids ratios (*n* = 8), fluid balance (*n* = 2), glycerides and phospholipids (*n* = 9), glycerides and phospholipid ratios (*n* = 2), glycolysis-related metabolites (*n* = 3), inflammation (*n* = 1), ketone bodies (*n* = 2), lipoprotein particle size (*n* = 3), lipoprotein subclasses (*n* = 98), and lipoprotein subclass ratios (*n* = 70).

### Data quality control

Prior to data analysis, metabolites and covariable quality control (QC) and data filtering steps were implemented. First, a single individual who self-described themselves as “other” rather than “White” was excluded from the analysis. Second, any nonmetabolite covariable with <1000 observations (*n* = 1), with no variation (*n* = 1), or binary covariables with fewer than 10 observations in either of the 2 binary classes (*n* = 1) were removed. A complete list of evaluated nonmetabolite covariables is available in Supplemental Table 2.

The initial metabolite data set consisted of 5744 individuals, 229 fasting metabolites, 229 postprandial metabolites, and 148 previously derived [29] orthogonal nonlinear least squares (ornls) response metabolite traits. Data QC of this data set was performed with the R package *metaboprep* [34]. A complete description of the procedure and parameters used with *metaboprep* can be found in Supplemental Methods. After data QC, 226 samples and 3 metabolites (all ornls response traits) were filtered from the data set. The log file and report generated by *metaboprep* are provided in Supplemental Materials. After running *metaboprep*, we also performed the following steps on the fasted and postprandial data. First, all zero values were converted into not applicable (NA) values. Second, for each metabolite (in the fasting and postprandial state, individually) all values of 10 interquartile distances from the median were also turned into NA values. Third, we used the expected correlated nature of the fasting and postprandial data to identify outliers of that relationship and turned them into NA values, in both dietary states. Further details on this procedure can be found in Supplemental Methods along with an illustration in Supplemental Figure 3.

### Response trait

A response trait, or a measure of change between the postprandial and fasting dietary state, was derived for each of the 229 metabolites traits by univariable Deming regression. A Deming regression was used in this study because it allows for error in both independent (fasting) and dependent (postprandial) variables of the model and, as such, is a type of total least squares regression. Following the above-described QC steps, we fit a Deming regression of postprandial on fasting data, for each metabolite. After model fitting, the residuals were extracted and used as the response trait. The function deming() from the deming (v1.4) R package was used in the analysis. The intercept and slope of Deming regression were recorded (Supplemental Table 1), along with trait annotations and population summary statistics for all metabolite traits.

The residuals of the Deming regressions derived in this study were used as metabolite traits of response. These response traits effectively mirror those of a simple delta, in that positive values indicate an increase in metabolite abundance in the postprandial state and negative values would indicate a decrease. Effect estimates from an association analysis with BMI, described further, therefore suggests whether response is associated with BMI. Positive effect estimates suggest that as BMI increases, so too does the change in metabolite abundance. Negative effect estimates suggest that as BMI increases the change in metabolite abundance decreases.

Deming regressions residuals were taken to be the most appropriate way to summarize response for second stage analyses—observational and MR—because it accounts for the measurement error in metabolite variation at both time points. However, in addition to the Deming regression, we also used 2 alternative methods to derive response. The first was a simple Δ estimate (postprandial minus fasting abundance) and the second was an ornls regression. We note that simple Δ values and Deming residuals correlate with a mean Pearson *r* of 0.94 (95% CI: 0.65, 0.99). The ornls response traits were derived and used previously in a genome-wide association study of metabolite response and was used in our metabolite QC steps described earlier [29]. Our result and discussion will focus exclusively on the Deming response traits, but all effect estimates for the Δ and ornls response traits are provided in Supplemental Table 3.

### Effective number of tested metabolites

Although there were 687 metabolite traits (fasting = 229, postprandial = 229, response = 229), these were not independent (Supplemental Figure 4). We used the R package “identification of principal variable” (iPV; https://github.com/hughesevoanth/iPVs) to estimate the effective number of metabolites in the data set [[35], [36], [37]]. This allowed us to ensure that we were not overcorrecting for the number tests we performed in the study. We estimated 43 representative variables in the NEO metabolite data set. The data-reduced, study-wide Bonferroni-corrected *P* value was set to 0.05/43 or 1.163 × 10^−3^. A full description of the iPV procedure and parameters can be found in Supplemental Methods.

### Metabolite data description

Two analyses were carried out to describe this Nightingale Health metabolomics data set. First, given the abundance of lipoproteins and their lipids in this data set, we estimated the mean abundance of each lipid for each lipoprotein in both the fasting and postprandial dietary states. Second, a paired Student *t* test was performed to determine whether mean abundances differed between the fasting and postprandial states. Our threshold for declaring a change in mean abundance was 0.05/229, where 229 is the number of metabolites tested. We also estimated the change as a Δ value between the postprandial and fasting states and then derived estimates for mean change and the 95% CI of the change distribution.

### Rank normal transformation of metabolite traits

Each metabolite trait distribution was tested for normality with a Shapiro-Wilk test. All W-statistics (untransformed distributions and model residuals) are reported in supplemental tables. In total, 55.56% of all metabolite traits had a W-statistics <0.95, a threshold used in this study to define an inconsistency with normality. A total of 43.94% of all log-transformed metabolite traits had a W-statistics of <0.95. Consequently, for the purpose of signal discovery and to allow for parametric analysis, each metabolite was rank normal transformed (tied values randomly ranked), prior to linear modelning. These steps resulted in the residuals from all observational and MR models to have a W-statistic >0.9 and >0.99, respectively.

### Covariables

Variables from 14 categories were compiled and available to identify possible confounders by evaluating associations with BMI, BMI-PGS, and metabolite traits (Supplemental Table 2). These 14 categories are as follows: anthropomorphic (*n* = 8); education (*n* = 5); income (*n* = 2); smoking (*n* = 3); diet including alcohol intake in grams (*n* = 24); medication including glucose and lipid lowering (*n* = 10); systolic and diastolic blood pressure (*n* = 2); health including diabetes, glucose, hypertension, and cancer status (*n* = 4); physical exercise (*n* = 18); basal metabolic rate (*n* = 1); indirect calorimetry (*n* = 5); and genotype principal components (*n* = 4). In addition, 7 metrics of biological sample quality (*n* = 7) and the sampling information (visit date and subpopulation, *n* = 2) were included in the study to assess influence on metabolites. During QC, described earlier, 3 variables were filtered [Nightingale Health low protein flag, education type, medication C07B (β-blockers)]. As performed with aforementioned metabolites, we estimated the effect number of covariables in the data set. After running an iPV analysis with a tree cut height of 0.5, we estimated that there are 54 representative or independent covariables. As a product any test performed across all covariables was corrected for multiple testing at a *P* value of 0.05/54 or 9.29 × 10^−4^.

### Genotype data

The NEO participants were genotyped on the Illumina HumanCoreExome-24 BeadChip (Illumina), at the Centre National de Génotypage (Evry Cedex, France). Following genotype QC steps, as previously described [38], sample were imputed to the Haplotype Reference Consortium release 1.1 [39], with further details in the study by Li-Gao et al. [29].

### Construction of the BMI-PGS

BMI-PGS was derived for each individual using variants previously and independently associated with BMI (*n* = 656) at a *P* value of <1 × 10^−8^ from the study by Yengo et al. [40]. To ensure the integrity of the PGS, only those genetic variants, in the NEO genotype dosage data set, where the effect allele, the alternative allele, and the minor allele frequency (±0.1) matched the data from Yengo et al. [40] were retained. If a match could not be made, then that single nucleotide polymorphism (SNP) was omitted. All effect estimates were aligned to be positive, such that the effect allele was always that which increased an individual’s genotype predicted BMI. Finally, a weighted PGS was constructed by weighing the number of BMI-increasing alleles, at each SNP (*n* = 646, after data harmonization), by the effect estimate for that SNP and then summing across all values [40]. The R script “01_generate_bmi_grs.R” for deriving the PGS can be found in the github repository https://github.com/hughesevoanth/NEO_BMI_Metabolite_MR.

### Observational and MR analysis

Two observational association analyses were undertaken in cross-section. The first was an association analysis between individual metabolite traits and BMI. The second uses a PGS as a proxy instrument for the exposure in a MR framework (Figure 1) to provide an estimate of the causal effect of the exposure on outcome (in this study, dietary state-specific metabolite values). We will refer to the first as an observational analysis and the later as an MR analysis.

Observational analyses were performed by a multivariable generalized linear regression with sampling date, subpopulation, age, and sex as additional covariables—taking the form of glm(metabolite ∼ visit date + subpopulation + age + sex + BMI). For each generalized linear model, we also performed a Breusch-Pagan test of homoskedasticity using the bptest() function from the lmtest R package [41]. In addition, for each association analysis, we performed a type I ANOVA, producing a table of deviances and estimated an η^2^ statistic, providing an estimate of the variance explained, for the model and for the primary exposure, BMI. These estimates are reported in supplemental tables.

One-sample MR analyses were performed using the ivreg R package (https://cran.r-project.org/web/packages/ivreg/), implementing a 2-stage least squares IV analysis. In all instances and given the relative viability of BMI instruments versus reciprocal metabolite instrumentation, BMI was defined as our exposure; the PGS described earlier was our instrument for BMI, and each metabolite trait was iteratively defined as the outcome. The same model and covariables as described earlier in the association analysis was used here. Along with MR effect estimates, we also report in supplemental tables the Breusch-Pagan test of homoskedasticity, the *F* statistic and *F* test *P* value testing for weak instruments, and summary statistics for the Durbin-Wu-Hausman endogeneity test.

Unless stated otherwise—because our outcomes are rank normal transformed (zero centered with a standard deviation of 1)—the effect estimates (*β*) and standard errors (SEs) are reported as rank-normalized standard deviation unit change per unit cross-sectional increase in BMI. We will refer to increases in BMI or increases in metabolite abundances throughout the article to infer cross-sectional increases in BMI, not individual-level units of change.

### Study structure

In all observational and MR analyses, 4 (sub)population analyses were performed. The primary analysis, in all instances, was a weighted Netherlands Epidemiology of Obesity (wNEO) regression analysis using data from all available samples. wNEO observational and MR summary statistics are reported in Supplemental Table 4. The wNEO analyses included weights for each sample that were previously derived to have the Leiden subpopulation BMI distribution emulate that of the randomly sampled Leiderdorp subpopulation [42]. Sensitivity analyses included the following: *1*) a wNEO analysis with additional covariables to account for possible confounders (Supplemental Table 5); *2*) a wNEO analysis with no transformation of the outcome traits (Supplemental Table 6); *3*) an unweighted NEO analysis (Supplemental Table 7); *4*) a Leiderdorp subpopulation analysis (Supplemental Table 8); and *5*) a Leiden subpopulation analysis (Supplemental Table 9). In addition, sex-specific analyses were performed using the primary wNEO framework (Supplemental Tables 10 and 11) and the Leiderdorp subpopulation (Supplemental Tables 12 and 13), as neither have a sampling bias in their BMI distributions.

### Data and code availability

Individual-level NEO data are available to researchers according to the NEO research procedure, which can be obtained by contacting the scientific director of the NEO study for access [32]. All statistical analysis were conducted in the R language (v 4.0.2, Taking Off Again), and all bespoke functions and analytical code for the study can be found in the github repository https://github.com/hughesevoanth/NEO_BMI_Metabolite_MR.

## Results

### NEO cohort description

Following QC and derivation of the response trait, the data set consisted of 5517 individuals (51.6% female), 687 metabolite traits (229 fasting, 229 postprandial, and 229 response), and 85 covariables. The mean age of participants was 56 y, with an mean BMI of 29.98 (Table 1).

**TABLE 1:** Population Summary Statistics

Trait	NEO	Males	Females	Leiderdorp	Leiden	Subpopulation *R*^2^ (%)	*P*
Sample size	5571	2671	2846	1406	4111	NA	NA
Females (*n*)	2846	0	2846	783	2063	0.17	3.65 × 10^−4^
Age (y)	55.99 (45.00–65.00)	56.13 (45.00–65.00)	55.86 (45.00–65.00)	56.13 (46.00–65.00)	55.95 (45.00–65.00)	0.02	3.26 × 10^−1^
Height (m)	1.74 (1.57–1.92)	1.81 (1.68–1.95)	1.67 (1.55–1.79)	1.73 (1.57–1.92)	1.74 (1.57–1.92)	0.03	2.03 × 10^−1^
Weight (kg)	90.58 (60.00–126.80)	97.45 (72.95–131.60)	84.14 (57.20–121.52)	79.16 (54.00–116.15)	94.49 (69.60–128.85)	16.12	9.63 × 10^−213^
Waist circumference (cm)	102.01 (74.00–129.00)	106.32 (85.79–131.00)	97.96 (71.00–126.00)	91.22 (70.00–119.00)	105.70 (86.00–131.00)	22.87	2.85 × 10^313^
Hip circumference (cm)	110.23 (94.00–133.00)	108.57 (96.00–125.00)	111.79 (92.00–138.00)	103.40 (90.00–124.17)	112.57 (99.00–135.00)	16.22	3.33 × 10^−214^
Pack-y	11.21 (0.00–52.38)	13.71 (0.00–61.69)	8.88 (0.00–43.83)	8.69 (0.00–43.32)	12.09 (0.00–55.20)	0.83	1.01 × 10^−10^
Alcohol	15.52 (0.00–60.52)	21.40 (0.00–73.24)	10.00 (0.00–41.72)	14.58 (0.00–54.06)	15.84 (0.00–62.57)	0.10	1.95 × 10^−2^
Smoking (never/former/current)	34.11/49.85/16.03	30.32/50.49/19.19	37.67/49.26/13.07	39.29/46.76/13.95	32.34/50.91/16.74	0.38	4.11 × 10^−6^
Higher education (%)	38.58	42.27	35.12	50.5	34.48	1.52	6.24 × 10^−26^
BMI (kg/m^2^)	29.98 (21.40–41.35)	29.74 (22.72–38.99)	30.20 (20.64–42.90)	26.24 (19.94–37.20)	31.26 (26.02–42.19)	20.70	3.66 × 10^−280^
BMI-PGS	10.20 (9.64–10.78)	10.20 (9.63–10.78)	10.20 (9.65–10.77)	10.14 (9.56–10.71)	10.22 (9.67–10.80)	1.63	1.53 × 10^−21^
*R*^2^	0.0431, 0.0473^1^	0.0439	0.0453	0.0427	0.0243	—	—
β (SE)	3.38 (0.21), 3.26 (0.20)^1^	2.77 (0.25)	4.00 (0.34)	3.14 (0.40)	2.25 (0.22)	—	—
*P*	8.10 × 10^−55^; 3.61 × 10^−60,^^1^	7.23 × 10^−28^	1.57 × 10^−30^	5.10 × 10^−15^	8.22 × 10^−24^	—	—

Population summary statistics for *1*) the complete NEO cohort, *2*) males, and *3*) females of the NEO cohort and the 2 NEO subpopulations; *4*) Leiderdorp; and *5*) Leiden. For each variable—age, height, weight, waist circumference, hip circumference, smoking in pack-years, alcohol consumption in grams per day, smoking (never/former/current), the percentage of the population with higher education, BMI, and BMI-PGS—the mean are provided along with the (95% quantile intervals) of all distributions. In addition, the number of females in each sample (sub)population is provided. The last 3 rows of data provide summary statistics describing the univariable linear relationship between the instrumental variables (BMI-PGS) on the exposure (BMI). Specifically, the variance explained (*R*^2^), the effect estimate (β) and standard error (SE), and the *P* value as derived by univariable linear regression are provided. The last 2 columns of data provide an estimate of the proportion of variation (in NEO) of the trait (in the row) explained by variation between the subpopulation (Leiderdorp vs. Leiden) samples (subpopulation *R*^2^) and the model *P* value, as estimated by a univariable linear regression.

Abbreviations: NEO, Netherlands Epidemiology of Obesity Study; PGS, polygenic score.

1 Estimates are derived from linear models that include sample weights (wNEO; see Methods and Study structure).

The NEO cohort is made up of 2 distinct subsamples, (1: “Leiderdorp”) a randomly sampled population of 1406 individuals from the municipality of Leiderdorp (mean BMI = 26.24, 95% quantile interval: 19.94, 37.2), and (2: “Leiden”) a sample population of 4111 participant from the city of Leiden who were oversampled for individuals with a BMI >27 (mean BMI = 31.26, 95% quantile interval: 26.02, 42.19). BMI distributions differed between the 2 subpopulations (generalized linear model Student *t* test *P* value = 3.66 × 10^−280^) (Supplemental Figure 1). In addition, the 2 subpopulations also differ in sex ratio, alcohol intake (in grams per day), smoking (pack-years), educational attainment, and BMI-PGS (generalized linear model Student *t* test *P* value of <1.95 × 10^−2^) but not in age or height (Table 1). Although the variance explained by subpopulations is large for BMI (20.7%) and closely related traits (weight, hip and waist circumference), it is an order smaller for other variables such as BMI-PGS, pack-years, and educational attainment (variance explained by subpopulation < 2%) (Table 1).

### Description of metabolite data

Of the 229 assayed metabolites 98 are lipoproteins and their particle concentrations and an additional 70 are lipoprotein ratios. The expected lipid concentrations were observed across lipoprotein classes (Supplemental Figure 5). Namely, triglycerides dominate VLDLs, cholesterol dominates intermediate density lipoprotein (IDL) and LDLs, and phospholipids and cholesterol dominate high density lipoproteins (HDLs), on average. This remains true in both the fasting and postprandial data. All except 14 metabolites differed in mean concentration or ratio between the fasted and postprandial states (paired *t* test, *P* < 0.05/229) (Supplemental Table 14). These 14 include the concentration of particles: total lipids, total cholesterol, cholesterol esters, and free cholesterol in medium LDL. It also includes total cholesterol in LDL, large LDL, and small LDL, and cholesterol esters in small LDL. Overall, 126 metabolites increased in abundance, and 89 decreased postprandially. Those with the largest centered and scaled, mean increase were the amino acids tyrosine, leucine, valine, isoleucine, and phenylalanine (Supplemental Figure 6). Those with the largest mean decrease were the ratio of saturated fatty acids to total fatty acids, the ratio of triglycerides to total lipids in very large VLDL, the ratio of phospholipids to total lipids in small VLDL and the ketone body 3-hydroxybutyrate. For all metabolites, 150-min postprandial change was not in the same direction for all participants (Supplemental Figure 6).

### Estimating the observational effect of BMI on metabolite trait variation

**FIGURE 2:**
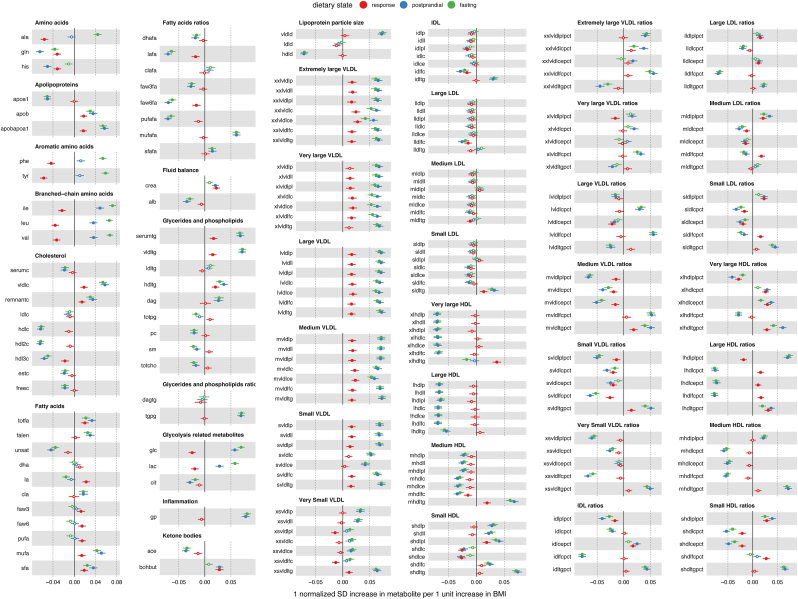
Observational effect estimate forest plot. Point estimates (β) and 95% CIs for BMI on fasting metabolite abundance (green), postprandial metabolite abundance (blue), and metabolite response (red). Estimates are derived from the weighted Netherlands Epidemiology of Obesity (wNEO) multivariable generalized linear regression that include sample date, subpopulation, age, and sex as covariables. Metabolites are classified and organized by Nightingale Health subclass assignments. Metabolites that are associated with BMI, after multiple test correction (*P* < 0.05/43), are indicated as solid color point estimates. Effect estimates are reported as rank-normalized standard deviation unit change per unit increase in BMI (kg/m^2^). All results can be found in Supplemental Table 4. Metabolite annotations and abbreviations can be found in Supplemental Table 1.

**FIGURE 3:**
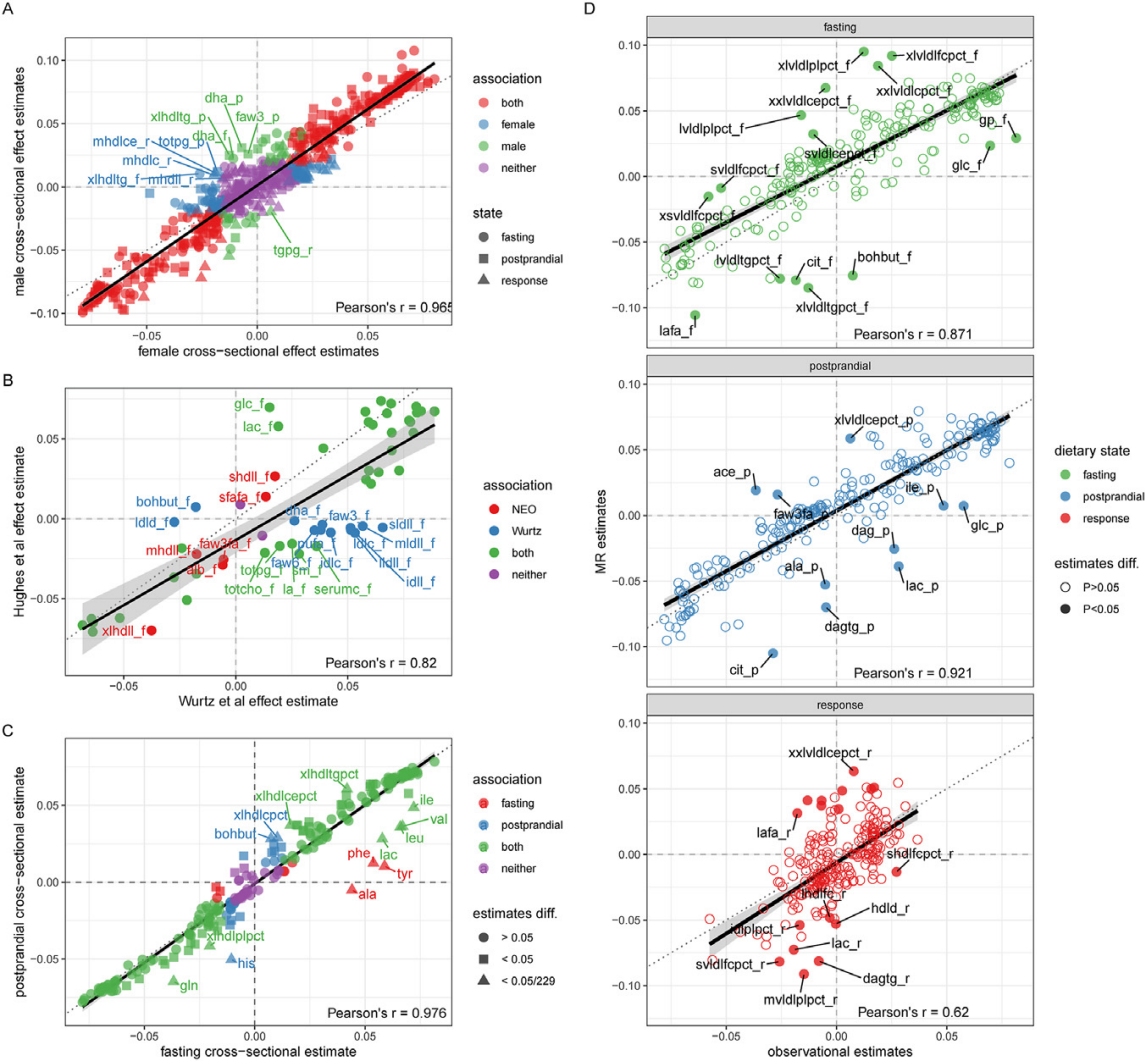
Scatter plots. In all plots, the solid black line is a best-fit regression line, the dotted black line is an equivalency line (intercept = 0, slope = 1), the dashed vertical and horizontal line indicate the zero values, and a Pearson correlation coefficient is in the bottom right corner. (A) Scatter plot of BMI–metabolite cross-sectional effect estimates for females (x-axis) and males (y-axis). Point estimates are colored red if there is an association between the metabolite and BMI in both sexes, blue if an association was only observed in females, green if an association was only observed in males, and purple if no association was observed in either sex. Point estimates are shaped as circles, squares, and triangles to represent the 3 dietary states fasting, postprandial, and response, respectively. All results for plot A can be found in Supplemental Tables 10 and 11. (B) Scatter plot of BMI–metabolite cross-sectional effect estimates from Wurtz et al. [15] (x-axis) and Netherlands Epidemiology of Obesity (NEO; this study, y-axis). Point estimates are colored red if there is an association between the metabolite and BMI in the NEO study, blue if an association was only observed in the study by Wurtz et al., green if an association was observed in both studies, and purple if no association was observed in either study. (C) Scatter plot of BMI–metabolite cross-sectional effect estimates for fasting (x-axis) and postprandial (y-axis) dietary states. Point estimates are colored red if there is an association between the metabolite and BMI in the fasting state, blue if an association was only observed in the postprandial state, green an association was observed in both dietary states, and purple if no association was observed in either. Point estimates are shaped as circles if they are not different between the 2 dietary states—as determined by a *z* test, shaped as squares if they are nominally different (*P* < 0.05), and triangles and labeled if they are different after correcting for multiple tests (*P* < 0.05/229). All results for plot C can be found in Supplemental Table 4. (D) Scatter plot of BMI–metabolite cross-sectional (x-axis) and Mendelian randomization (MR) (y-axis) effect in the fasting (top and green), postprandial (middle and blue), and response (bottom and red) dietary states. Metabolites with effect estimates that differ between the cross-sectional and MR analyses are solid circles and labeled with the metabolite name. All results for plot D can be found in Supplemental Table 4. Metabolite annotations and abbreviations can be found in Supplemental Table 1.

wNEO observational analyses suggested broad association between BMI and metabolite traits, with 473 metabolite traits (across fasting, postprandial, and response states) showing evidence of association with BMI (*P* < 1.163 × 10^−3^) (Figure 2, Supplemental Table 4, Supplemental Figure 7). Effect estimates correlated between sexes (Pearson *r* = 0.965), but more associations were observed in females (426 total: 162 fasting, 175 postprandial, 89 response) than males (369 total: 161 fasting, 168 postprandial, 40 response) (Supplemental Figure 4). A total of 333 associations were shared between females and males, with 93 specific to females, 36 specific to males, and 225 metabolite traits having no association with BMI in either sex (Supplemental Tables 10 and 11). Among the associated traits all but 10 are directionally consistent among females and males (Figure 3A). Larger effect sizes were observed in males (mean absolute β: 0.0356) than those in females (mean β: 0.0291), across all traits on average (Figure 3A), but the variance explained by BMI, across all traits on average was similar among males and females (η^2^: males = 1.737%, females = 1.734%).

#### Observational results—fasting

A total of 175 or 76% of fasting traits showed evidence of association with BMI. The strongest association observed was with the inflammation marker glycoprotein acetyls (β: 0.081; SE: 0.0037; *P* = 2.89 × 10^−101^). This was followed closely by the branched-chain amino acids (BCAA) isoleucine (β: 0.072; SE: 0.0033; *P* = 4.18 × 10^−101^), leucine (β: 0.066; SE: 0.0031; *P* = 1.16 × 10^−94^), and valine (β: 0.067; SE: 0.0032; *P* = 8.70 × 10^−92^). The strongest inverse associations were observed for mean diameter of HDL particles (β:−0.071; SE: 0.0035; *P* = 6.74 × 10^−95^), the ratio of free cholesterol to total lipids in IDL (β:−0.078; SE: 0.0036; *P* = 1.04 × 10^−100^), and large HDL (β:−0.077; SE: 0.0037; *P* = 8.09 × 10^−95^). If data are summarized by metabolite annotation class (Supplemental Table 1), we find that the classes with the largest mean absolute BMI effect are inflammation (mean absolute β: 0.081), amino acids (0.051), lipoprotein particle size (0.049), and glycolysis-related metabolites (0.049). Those with the smallest mean absolute effect are ketone bodies (0.021), fluid balance (0.019) and fatty acids (0.018) (Supplemental Figure 7).

#### Observational results—postprandial

A total of 188 or 82% of postprandial traits showed evidence of association with BMI. Fasting and postprandial point estimates correlated strongly (Pearson *r* = 0.976) with each other, with no mean difference in their distributions (*t* test, *P* = 0.83) and 166 shared associations. However, 31 associations are unique to 1 of the 2 dietary states, with 9 associations specific to fasting and 22 specific to postprandial data (Figure 3C). We tested for a difference in effect estimates between the dietary states and observed 14 estimates to differ (*z* test, *P* < 0.05/229) (Figure 3C). Eight of those 14 are amino acids (tyrosine, alanine, phenylalanine, histidine, valine, leucine, glutamine, and isoleucine). The other 6 that differ between dietary states include 4 very large HDL ratios, the ketone β-hydroxybutyrate, and the glycolysis-related metabolite lactate.

#### Observational results—response

**FIGURE 4:**
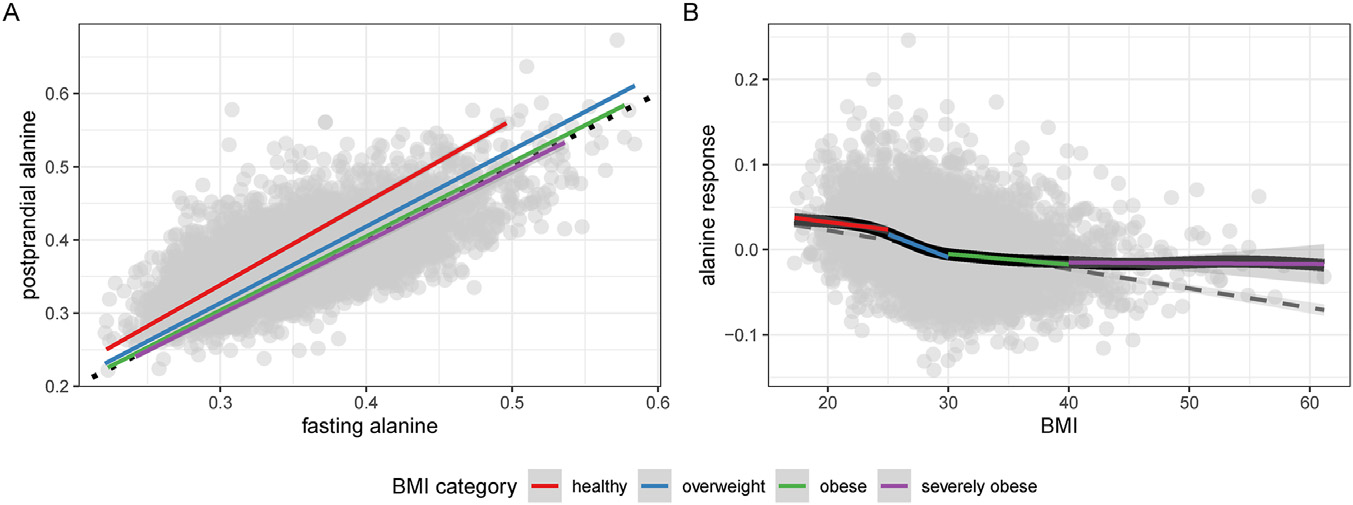
Alanine response. (A) Scatter plot illustrating the correlation between fasting (x-axis) and postprandial (y-axis) alanine abundance, with a best-fit line for 4 BMI (kg/m^2^) classes of individuals: healthy (BMI < 25), overweight (25 ≤ BMI < 30), obese (30 ≤ BMI < 40), and severely obese (BMI > 40). All best-fit lines were forced through an intercept of zero. Note that the slope of the best-fit line is the largest for individuals who have a healthy BMI and the smallest for those who have severe obesity. (B) Scatter plot illustrating the inverse relationship between BMI (x-axis) and alanine response (y-axis). The blue line is a best-fit linear regression line; the black line is a best-fit generalized additive model smooth (nonlinear regression); and the red, blue, green, and purple line intervals are best-fit regression for each clinically defined portion of the BMI distribution, which mirror the nonlinear regression.

A total of 70 response traits showed evidence of a positive association with BMI and 40 traits had an inverse association. Response and fasting (Pearson *r* = 0.34; *P* = 1.39 × 10^−7^) and response and postprandial (Pearson *r* = 0.514; *P* = 7.96 × 10^−17^) point estimates modestly correlated with each other, indicative of the relative independence of the response trait. The strongest associations were all amino acids, which (ordered by association *P* value: tyrosine, alanine, phenylalanine, leucine, valine, glutamine, histidine, and isoleucine) were all inversely associated with BMI (Supplemental Figure 7). For example, although alanine levels (fasting mean = 0.359 mmol/L) were elevated after a liquid mixed meal (postprandial mean = 0.376 mmol/L; paired *t* test, *P* = 2.53 × 10^−181^), the effect attenuated as BMI increased (β:−0.056; SE: 0.004; *P* = 5.72 × 10^−51^) (Figure 4A) such that the effect of BMI on alanine response was negative (Figure 4B). This relationship was observed for all amino acids in the data set (Figure 2).

The strongest positive response association was observed for triglycerides in very large HDL (β: 0.037; SE: 0.004; *P* = 5.88 × 10^−21^). This was followed closely by the ratio of *1*) cholesterol esters, *2*) triglycerides, and *3*) total cholesterol to total lipids in very large HDL. Conversely, the response trait ratio of phospholipids to total lipids in very large HDL decreased as BMI increased. This suggests that after a liquid mixed meal, the increase of phospholipids relative to total lipids in very large HDL is attenuated by BMI and, conversely, the ratio of cholesterol and triglycerides to total lipids in very large HDL increased as BMI increased (Figure 2).

Outside of amino acids and lipoproteins, other metabolite response traits that had a positive association with BMI included 3-hydroxybutyrate (ketone body), creatinine (fluid balance), linoleic acid, and total fatty acids (fatty acids). Others with an inverse association with BMI included glucose and lactate (glycolysis), the ratio of linoleic acid to total fatty acids, and the ratio of ω-6 (n–6) fatty acids to total fatty acids (fatty acid ratios) (Supplemental Table 4).

### Possible confounders

An exhaustive assessment of the association between BMI and BMI-PGS with 91 study covariables was performed, by univariable linear modeling and analysis of variance, to identify possible confounders or those that may violate the second MR assumption (exchangeability). In brief, 60 covariables were associated with BMI (wNEO; *P* < 9.26 × 10^−4^), and 17 were associated with BMI-PGS (Supplemental Figures 8 and 9, Supplemental Table 15). The 17 covariables that associated with the BMI-PGS were tested for an association with each metabolite trait (the outcomes), by univariable linear modeling, to identify possible confounders that violate MR assumption 2 (Figure 1). Results illustrated broad association across all traits and covariables, defining each covariable as a confounder in MR analysis leading to sensitivity analyses discussed further (Supplemental Figure 10). A more comprehensive description of these analyses is in Supplemental Results.

### Estimating the causal effect of BMI on metabolite trait variation

MR analyses (wNEO) support broad associations between BMI and metabolites as suggested by the overall strong correlation between observational and MR estimates (Pearson *r* = 0.852; *P* = 1.02 × 10^−194^; intercept = 1.54 × 10^−3^; slope = 0.911). The correlation was the strongest for postprandial traits (Pearson *r* = 0.921; *P* = 4.44 × 10^−95^; intercept = 3.59 × 10^−3^; slope = 0.922) and weaker for fasting (Pearson *r* = 0.871; *P* = 7.71 × 10^−72^; intercept = 7.62 × 10^−3^; slope = 0.855) and response traits (Pearson *r* = 0.62; *P* = 1.11 × 10^−25^; intercept = −6.36^−3^; slope = 1.08) (Figure 3D). Forty-two observational and MR effect estimates showed evidence of being different from each other (*z* test, *P* < 0.05), yet none remain so after correcting for multiple tests (*P* < 0.05/687). Overall, these observations indicate that cross-sectional estimates have reasonable power at predicting MR estimates for these exposure-outcome analyses, consistent with previous work [15].

**FIGURE 5:**
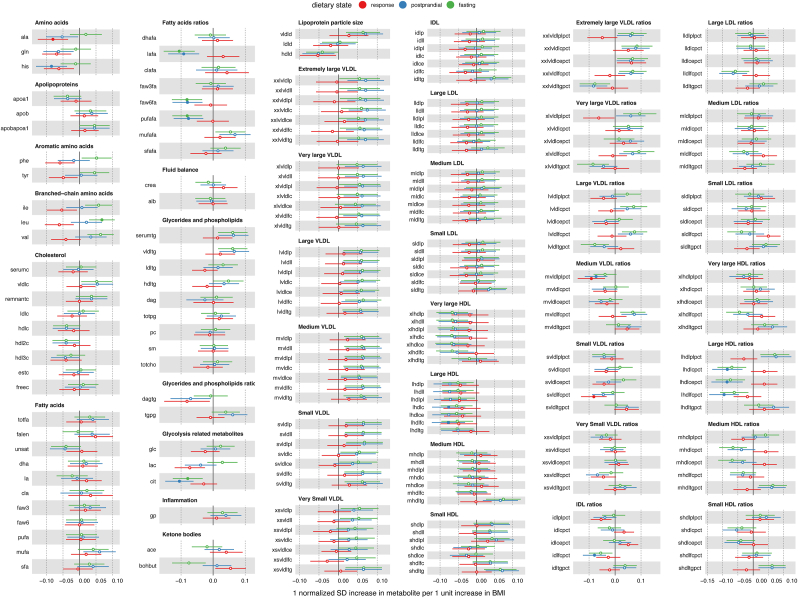
Forest plot of Mendelian randomization (MR) effect estimates. MR point estimates and 95% CIs for BMI on fasting metabolite abundance (green), postprandial metabolite abundance (blue), and metabolite response (red). MR estimates are derived from the weighted Netherlands Epidemiology of Obesity multivariable generalized linear regression that include sample date, subpopulation, age, and sex as covariables. Metabolites are classified and organized by Nightingale Health subclass assignments. Metabolites that are associated with BMI, after multiple test correction (*P* < 0.05/43), are indicated as solid color point estimates. Effect estimates are reported as rank-normalized standard deviation unit change per unit increase in BMI (kg/m^2^). All results can be found in Supplemental Table 4. Metabolite annotations and abbreviations can be found in Supplemental Table 1. ace, acetate; ala, alanine; alb, albumin; apoa1, apolipoprotein A-I; apob, apolipoprotein B; apobapoa1, ratio of apolipoprotein B to apolipoprotein A-I; bohbut, 3-hydroxybutyrate; cit, citrate; cit_f, fasting citrate; cit_p, postprandial citrate; cla, conjugated linoleic acid; clafa, ratio of conjugated linoleic acid to total fatty acids; crea, creatinine; dag, diacylglycerol; dagtg, ratio of diacylglycerol to triglycerides; dha, 22:6, docosahexaenoic acid; dhafa, ratio of 22:6 docosahexaenoic acid to total fatty acids; estc, esterified cholesterol; falen, estimated description of fatty acid chain length, not actual carbon number; faw3, ω-3 fatty acids; faw3fa, ratio of ω-3 fatty acids to total fatty acids; faw6, ω-6 fatty acids; faw6fa, ratio of ω-6 fatty acids to total fatty acids; freec, free cholesterol; glc, glucose; gln, glutamine; gln_p, postprandial glutamine; gp, glycoprotein acetyls, mainly a1-acid glycoprotein; hdl2c, total cholesterol in HDL2; hdl3c, total cholesterol in HDL3; hdlc, total cholesterol in HDL; hdld, mean diameter for HDL particles; hdltg, triglycerides in HDL; his, histidine; iPVs, identification of principal variables; idlc, total cholesterol in IDL; idlce, cholesterol esters in IDL; idlcepct, cholesterol esters in IDL to total lipids in IDL ratio; idlcpct, total cholesterol in IDL to total lipids in IDL ratio; idlfc, free cholesterol in IDL; idlfcpct, free cholesterol in IDL to total lipids in IDL ratio; idll, total lipids in IDL; idlp, concentration of IDL particles; idlpl, phospholipids in IDL; idlplpct, phospholipids in IDL to total lipids in IDL ratio; idltg, triglycerides in IDL; idltgpct, triglycerides in IDL to total lipids in IDL ratio; ile, isoleucine; la, 18:2, linoleic acid; lac, lactate; lafa, ratio of 18:2 linoleic acid to total fatty acids; lafa_f, fasting ratio of linoleic acid to total fatty acids; lafa_p, postprandial ratio of linoleic acid to total fatty acids; ldlc, total cholesterol in LDL; ldld, mean diameter for LDL particles; ldltg, triglycerides in LDL; leu, leucine; lhdlc, total cholesterol in large HDL; lhdlce, cholesterol esters in large HDL; lhdlcepct, cholesterol esters in large HDL to total lipids in large HDL ratio; lhdlcpct, total cholesterol in large HDL to total lipids in large HDL ratio; lhdlfc, free cholesterol in large HDL; lhdlfcpct, free cholesterol in large HDL to total lipids in large HDL ratio; lhdll, total lipids in large HDL; lhdlp, concentration of large HDL particles; lhdlpl, phospholipids in large HDL; lhdlplpct, phospholipids in large HDL to total lipids in large HDL ratio; lhdltg, triglycerides in large HDL; lhdltgpct, triglycerides in large HDL to total lipids in large HDL ratio; lldlc, total cholesterol in large LDL; lldlce, cholesterol esters in large LDL; lldlcepct, cholesterol esters in large LDL to total lipids in large LDL ratio; lldlcpct, total cholesterol in large LDL to total lipids in large LDL ratio; lldlfc, free cholesterol in large LDL; lldlfcpct, free cholesterol in large LDL to total lipids in large LDL ratio; lldll, total lipids in large LDL; lldlp, concentration of large LDL particles; lldlpl, phospholipids in large LDL; lldlplpct, phospholipids in large LDL to total lipids in large LDL ratio; lldltg, triglycerides in large LDL; lldltgpct, triglycerides in large LDL to total lipids in large LDL ratio; lvldlc, total cholesterol in large VLDL; lvldlce, cholesterol esters in large VLDL; lvldlcepct, cholesterol esters in large VLDL to total lipids in large VLDL ratio; lvldlcpct, total cholesterol in large VLDL to total lipids in large VLDL ratio; lvldlfc, free cholesterol in large VLDL; lvldlfcpct, free cholesterol in large VLDL to total lipids in large VLDL ratio; lvldll, total lipids in large VLDL; lvldlp, concentration of large VLDL particles; lvldlpl, phospholipids in large VLDL; lvldlplpct, phospholipids in large VLDL to total lipids in large VLDL ratio; lvldltg, triglycerides in large VLDL; lvldltgpct, triglycerides in large VLDL to total lipids in large VLDL ratio; mhdlc, total cholesterol in medium HDL; mhdlce, cholesterol esters in medium HDL; mhdlcepct, cholesterol esters in medium HDL to total lipids in medium HDL ratio; mhdlcpct, total cholesterol in medium HDL to total lipids in medium HDL ratio; mhdlfc, free cholesterol in medium HDL; mhdlfcpct, free cholesterol in medium HDL to total lipids in medium HDL ratio; mhdll, total lipids in medium HDL; mhdlp, concentration of medium HDL particles; mhdlpl, phospholipids in medium HDL; mhdlplpct, phospholipids in medium HDL to total lipids in medium HDL ratio; mhdltg, triglycerides in medium HDL; mhdltgpct, triglycerides in medium HDL to total lipids in medium HDL ratio; mldlc, total cholesterol in medium LDL; mldlce, cholesterol esters in medium LDL; mldlcepct, cholesterol esters in medium LDL to total lipids in medium LDL ratio; mldlcpct, total cholesterol in medium LDL to total lipids in medium LDL ratio; mldlfc, free cholesterol in medium LDL; mldlfcpct, free cholesterol in medium LDL to total lipids in medium LDL ratio; mldll, total lipids in medium LDL; mldlp, concentration of medium LDL particles; mldlpl, phospholipids in medium LDL; mldlplpct, phospholipids in medium LDL to total lipids in medium LDL ratio; mldltg, triglycerides in medium LDL; mldltgpct, triglycerides in medium LDL to total lipids in medium LDL ratio; mufa, monounsaturated fatty acids; 16:1, 18:1; mufafa, Ratio of monounsaturated fatty acids to total fatty acids; mvldlc, total cholesterol in medium VLDL; mvldlce, cholesterol esters in medium VLDL; mvldlcepct, cholesterol esters in medium VLDL to total lipids in medium VLDL ratio; mvldlcpct, total cholesterol in medium VLDL to total lipids in medium VLDL ratio; mvldlfc, free cholesterol in medium VLDL; mvldlfcpct, free cholesterol in medium VLDL to total lipids in medium VLDL ratio; mvldll, total lipids in medium VLDL; mvldlp, concentration of medium VLDL particles; mvldlpl, phospholipids in medium VLDL; mvldlplpct, phospholipids in medium VLDL to total lipids in medium VLDL ratio; mvldltg, triglycerides in medium VLDL; mvldltgpct, triglycerides in medium VLDL to total lipids in medium VLDL ratio; ornls, orthogonal nonlinear least squares; pc, phosphatidylcholine and other cholines; phe, Phenylalanine; pufa, polyunsaturated fatty acids; pufafa, ratio of polyunsaturated fatty acids to total fatty acids; remnantc, remnant cholesterol; se, standard error; serumc, serum total cholesterol; serumtg, serum total triglycerides; sfa, saturated fatty acids; sfafa, ratio of saturated fatty acids to total fatty acids; shdlc, total cholesterol in small HDL; shdlce, cholesterol esters in small HDL; shdlcepct, cholesterol esters in small HDL to total lipids in small HDL ratio; shdlcpct, total cholesterol in small HDL to total lipids in small HDL ratio; shdlfc, free cholesterol in small HDL; shdlfcpct, free cholesterol in small HDL to total lipids in small HDL ratio; shdll, total lipids in small HDL; shdlp, concentration of small HDL particles; shdlpl, phospholipids in small HDL; shdlplpct, phospholipids in small HDL to total lipids in small HDL ratio; shdltg, triglycerides in small HDL; shdltg_f, fasting small HDL triglycerides; shdltgpct, triglycerides in small HDL to total lipids in small HDL ratio; sldlc, total cholesterol in small LDL; sldlce, cholesterol esters in small LDL; sldlcepct, cholesterol esters in small LDL to total lipids in small LDL ratio; sldlcpct, total cholesterol in small LDL to total lipids in small LDL ratio; sldlfc, free cholesterol in small LDL; sldlfcpct, free cholesterol in small LDL to total lipids in small LDL ratio; sldll, total lipids in small LDL; sldlp, concentration of small LDL particles; sldlpl, phospholipids in small LDL; sldlplpct, phospholipids in small LDL to total lipids in small LDL ratio; sldltg, triglycerides in small LDL; sldltgpct, triglycerides in small LDL to total lipids in small LDL ratio; sm, sphingomyelins; svldlc, total cholesterol in small VLDL; svldlce, cholesterol esters in small VLDL; svldlcepct, cholesterol esters in small VLDL to total lipids in small VLDL ratio; svldlcpct, total cholesterol in small VLDL to total lipids in small VLDL ratio; svldlfc, free cholesterol in small VLDL; svldlfcpct, free cholesterol in small VLDL to total lipids in small VLDL ratio; svldll, total lipids in small VLDL; svldlp, concentration of small VLDL particles; svldlpl, phospholipids in small VLDL; svldlplpct, phospholipids in small VLDL to total lipids in small VLDL ratio; svldltg, triglycerides in small VLDL; svldltgpct, triglycerides in small VLDL to total lipids in small VLDL ratio; tgpg, ratio of triglycerides to phosphoglycerides ratio; totcho, total cholines; totfa, total fatty acids; totpg, total phosphoglycerides; tyr, tyrosine; unsat, estimated degree of unsaturation; val, valine; vldlc, total cholesterol in VLDL; vldld, mean diameter for VLDL particles; vldltg, triglycerides in VLDL; wNEO, weighted NEO regression analysis, the primary analysis framework; xlhdlc, total cholesterol in very large HDL; xlhdlce, cholesterol esters in very large HDL; xlhdlcepct, cholesterol esters in very large HDL to total lipids in very large HDL ratio; xlhdlcpct, total cholesterol in very large HDL to total lipids in very large HDL ratio; xlhdlfc, free cholesterol in very large HDL; xlhdlfcpct, free cholesterol in very large HDL to total lipids in very large HDL ratio; xlhdll, total lipids in very large HDL; xlhdlp, concentration of very large HDL particles; xlhdlpl, phospholipids in very large HDL; xlhdlplpct, phospholipids in very large HDL to total lipids in very large HDL ratio; xlhdltg, triglycerides in very large HDL; xlhdltgpct, triglycerides in very large HDL to total lipids in very large HDL ratio; xlvldlc, total cholesterol in very large VLDL; xlvldlce, cholesterol esters in very large VLDL; xlvldlcepct, cholesterol esters in very large VLDL to total lipids in very large VLDL ratio; xlvldlcpct, total cholesterol in very large VLDL to total lipids in very large VLDL ratio; xlvldlfc, free cholesterol in very large VLDL; xlvldlfcpct, free cholesterol in very large VLDL to total lipids in very large VLDL ratio; xlvldll, total lipids in very large VLDL; xlvldlp, concentration of very large VLDL particles; xlvldlpl, phospholipids in very large VLDL; xlvldlplpct, phospholipids in very large VLDL to total lipids in very large VLDL ratio; xlvldltg, triglycerides in very large VLDL; xlvldltgpct, triglycerides in very large VLDL to total lipids in very large VLDL ratio; xsvldlc, total cholesterol in very small VLDL; xsvldlce, cholesterol esters in very small VLDL; xsvldlcepct, cholesterol esters in very small VLDL to total lipids in very small VLDL ratio; xsvldlcpct, total cholesterol in very small VLDL to total lipids in very small VLDL ratio; xsvldlfc, free cholesterol in very small VLDL; xsvldlfcpct, free cholesterol in very small VLDL to total lipids in very small VLDL ratio; xsvldll, total lipids in very small VLDL; xsvldlp, concentration of very small VLDL particles; xsvldlpl, phospholipids in very small VLDL; xsvldlplpct, phospholipids in very small VLDL to total lipids in very small VLDL ratio; xsvldltg, triglycerides in very small VLDL; xsvldltg_f, fasting very small VLDL triglycerides; xsvldltgpct, triglycerides in very small VLDL to total lipids in very small VLDL ratio; xxlvldlc, total cholesterol in chylomicrons and extremely large VLDL; xxlvldlce, cholesterol esters in chylomicrons and extremely large VLDL; xxlvldlcepct, cholesterol esters in chylomicrons and extremely large VLDL to total lipids in chylomicrons and extremely large VLDL ratio; xxlvldlcpct, total cholesterol in chylomicrons and extremely large VLDL to total lipids in chylomicrons and extremely large VLDL ratio; xxlvldlfc, free cholesterol in chylomicrons and extremely large VLDL; xxlvldlfcpct, free cholesterol in chylomicrons and extremely large VLDL to total lipids in chylomicrons and extremely large VLDL ratio; xxlvldll, total lipids in chylomicrons and extremely large VLDL; xxlvldlp, concentration of chylomicrons and extremely large VLDL particles; xxlvldlpl, phospholipids in chylomicrons and extremely large VLDL; xxlvldlplpct, phospholipids in chylomicrons and extremely large VLDL to total lipids in chylomicrons and extremely large VLDL ratio; xxlvldltg, triglycerides in chylomicrons and extremely large VLDL; xxlvldltgpct, triglycerides in chylomicrons and extremely large VLDL to total lipids in chylomicrons and extremely large VLDL ratio.

**FIGURE 6:**
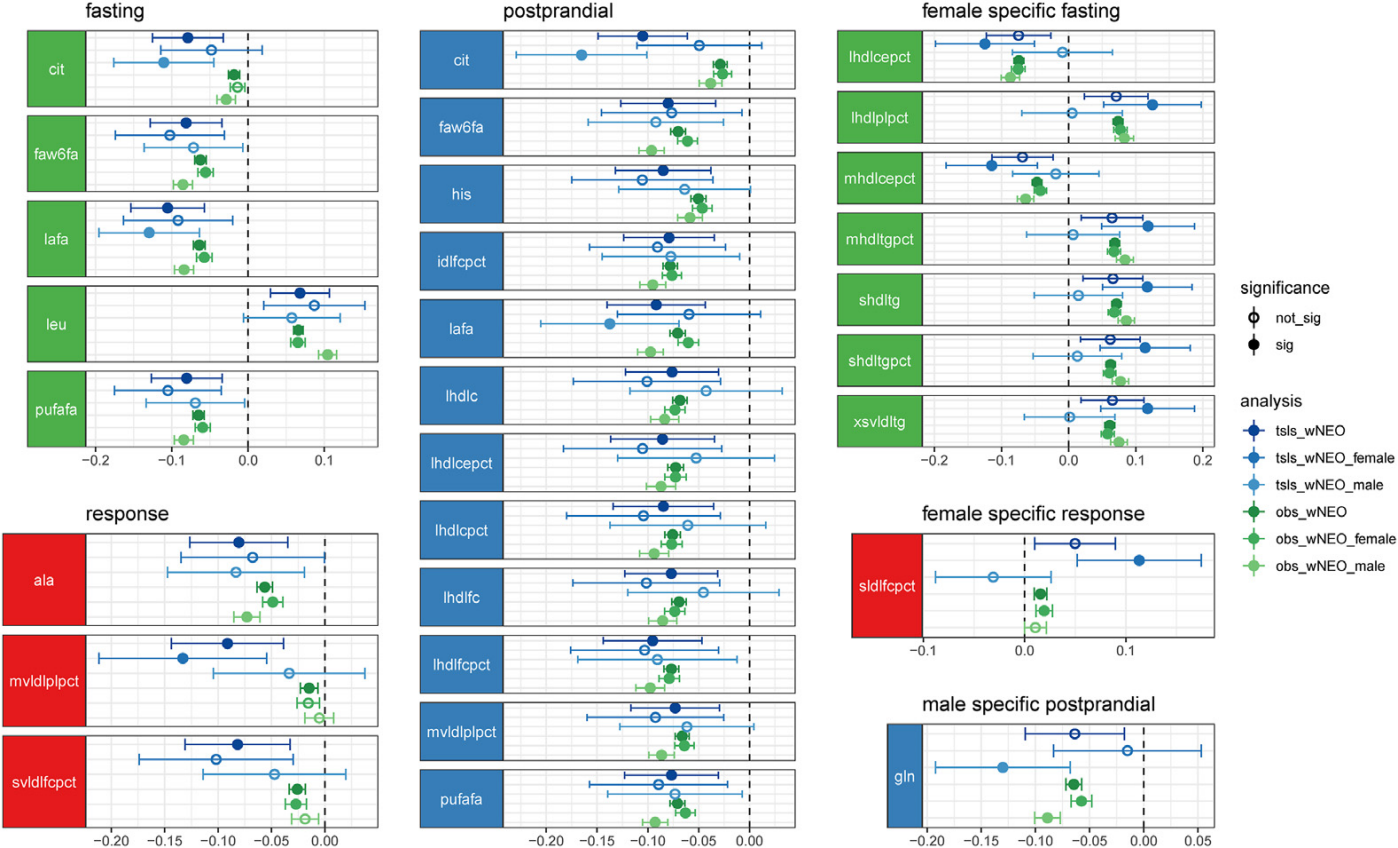
Metabolites associated with BMI in Mendelian randomization (MR) analyses. A forest plot of effect estimates (points) and 95% CIs (whiskers) for MR (blue, “tsls” prefix) and observational (green, “obs” prefix) effect estimates for the weighted Netherlands Epidemiology of Obesity (wNEO), weighted NEO female (wNEO_female), and weighted NEO male (wNEO_male) (sub)populations. Fasting, postprandial, and response association observed in the general population and those association seen in only females and males. All results can be found in Supplemental Tables 4, 10, and 11, respectively. Metabolite annotations and abbreviations can be found in Supplementary Table 1. ala, alanine; cit, citrate; faw6fa, ratio of ω-6 fatty acids to total fatty acids; gln, Glutamine; his, histidine; idlfcpct, free cholesterol in IDL to total lipids in IDL ratio; lafa, ratio of 18:2 linoleic acid to total fatty acids; leu, Leucine; lhdlc, total cholesterol in large HDL; lhdlcepct, cholesterol esters in large HDL to total lipids in large HDL ratio; lhdlcpct, total cholesterol in large HDL to total lipids in large HDL ratio; lhdlfcpct, free cholesterol in large HDL to total lipids in large HDL ratio; lhdlplpct, phospholipids in large HDL to total lipids in large HDL ratio; mhdlcepct, cholesterol esters in medium HDL to total lipids in medium HDL ratio; mhdltgpct, triglycerides in medium HDL to total lipids in medium HDL ratio; mvldlplpct, phospholipids in medium VLDL to total lipids in medium VLDL ratio; pufafa, ratio of polyunsaturated fatty acids to total fatty acids; shdltg, triglycerides in small HDL; shdltgpct, triglycerides in small HDL to total lipids in small HDL ratio; sldlfcpct, free cholesterol in small LDL to total lipids in small LDL ratio; svldlfcpct, free cholesterol in small VLDL to total lipids in small VLDL ratio; xsvldltg, triglycerides in very small VLDL.

A total of 201 metabolite traits (across fasting, postprandial, and response states) (Supplemental Table 4, Figure 5) showed nominal evidence of association (*P* < 0.05) with BMI and 20 (Table 2, Figure 6) showed evidence of association with BMI (*P* < 1.16 × 10^−3^) (Supplemental Figure 11). MR effect estimates varied more between sexes (Pearson *r* = 0.368; *P* = 1.93 × 10^−23^) than observed in observational results, with 86 showing evidence of being different from each other (*z* test, *P* < 0.05), yet none remaining so after correcting for multiple tests (*P* < 0.05/687). Nine metabolite traits (7 fasting and 2 response) showed evidence of association with BMI in females, 8 of which were unique to females (Figure 6). This included fasting small HDL triglycerides and fasting very small VLDL triglycerides, both of which increased with increases in BMI. By contrast, 5 metabolites were associated with BMI in males, one of which was unique to males—postprandial glutamine, which had an inverse relationship with BMI (Figure 6). The other 4 metabolites shared an association with the general population. They are fasting and postprandial citrate and the ratio of linoleic acid to total fatty acids in both the fasting and postprandial states. Moreover, each of them were also inversely associated with BMI. Mean MR effect estimates were larger in females (absolute mean β: 0.047) than those in males (absolute mean β: 0.026; paired *t* test, *P* = 1.43 × 10^−45^).

**TABLE 2:** Metabolite Traits Causally Associated With BMI

Trait	Metabolite	Class	Dietary state	β	SE	*P*
lafa_f	Ratio of linoleic acid (18:2) to total fatty acids (%)	Fatty acids ratios	Fasting	−0.106	0.025	1.80 × 10^−5^
faw6fa_f	Ratio of ω-6 fatty acids to total fatty acids (%)	Fatty acids ratios	Fasting	−0.081	0.024	6.97 × 10^−4^
pufafa_f	Ratio of polyunsaturated fatty acids to total fatty acids (%)	Fatty acids ratios	Fasting	−0.081	0.024	6.80 × 10^−4^
cit_f	Citrate (mmol/L)	Glycolysis-related metabolites	Fasting	−0.079	0.024	8.63 × 10^−4^
leu_f	Leucine (mmol/L)	Amino acids	Fasting	0.068	0.020	5.62 × 10^−4^
lhdlc_p	Total cholesterol in large HDL (mmol/L)	Lipoprotein subclasses	Postprandial	−0.076	0.023	1.12 × 10^−3^
lhdlfc_p	Free cholesterol in large HDL (mmol/L)	Lipoprotein subclasses	Postprandial	−0.077	0.023	9.73 × 10^−4^
mvldlplpct_p	Phospholipids in medium VLDL to total lipids in medium VLDL ratio (%)	Lipoprotein subclass ratios	Postprandial	−0.073	0.022	9.98 × 10^−4^
idlfcpct_p	Free cholesterol in IDL to total lipids in IDL ratio (%)	Lipoprotein subclass ratios	Postprandial	−0.079	0.023	5.08 × 10^−4^
lhdlcpct_p	Total cholesterol in large HDL to total lipids in large HDL ratio (%)	Lipoprotein subclass ratios	Postprandial	−0.085	0.025	7.90 × 10^−4^
lhdlcepct_p	Cholesterol esters in large HDL to total lipids in large HDL ratio (%)	Lipoprotein subclass ratios	Postprandial	−0.086	0.026	1.03 × 10^−3^
lhdlfcpct_p	Free cholesterol in large HDL to total lipids in large HDL ratio (%)	Lipoprotein subclass ratios	Postprandial	−0.095	0.025	1.20 × 10^−4^
lafa_p	Ratio of linoleic acid (18:2) to total fatty acids (%)	Fatty acids ratios	Postprandial	−0.092	0.025	2.00 × 10^−4^
faw6fa_p	Ratio of ω-6 fatty acids to total fatty acids (%)	Fatty acids ratios	Postprandial	−0.080	0.024	7.83 × 10^−4^
pufafa_p	Ratio of polyunsaturated fatty acids to total fatty acids (%)	Fatty acids ratios	Postprandial	−0.077	0.024	1.10 × 10^−3^
cit_p	Citrate (mmol/L)	Glycolysis-related metabolites	Postprandial	−0.105	0.022	2.83 × 10^−6^
his_p	Histidine (mmol/L)	Amino acids	Postprandial	−0.085	0.024	3.96 × 10^−4^
mvldlplpct_r	Phospholipids in medium VLDL to total lipids in medium VLDL ratio (%)	Lipoprotein subclass ratios	Response	−0.091	0.027	6.75 × 10^−4^
svldlfcpct_r	Free cholesterol in small VLDL to total lipids in small VLDL ratio (%)	Lipoprotein subclass ratios	Response	−0.082	0.025	1.11 × 10^−3^
ala_r	Alanine (mmol/L)	Amino acids	Response	−0.081	0.023	5.91 × 10^−4^

MR summary statistics for the 20 metabolite traits causally associated with BMI. Provided are the trait name, metabolite description and units, metabolite class as defined by Nightingale Heath, the dietary state, and the MR effect estimate, standard error, and *P* value. All summary statistics were derived from the wNEO 2-stage-least squares 1-sample MR framework, using the NEO data set with a sample size of 5744. However, each metabolite has a varying amount of missingness, and exact sample sizes for each trait can be found in Supplemental Table 4. The effect estimates are in normalized standard deviation units of change per 1 unit increase of BMI (kg/m^2^).

Abbreviations: ala_r, alanine response; cit_f, fasting citrate; cit_p, postprandial citrate; faw6fa_f, fasting ratio of omega-6 fatty acids to total fatty acids; faw6fa_p, postprandial omega-6 fatty acids to total fatty acids; HDL, high density lipoprotein; his_p postprandial histidine; IDL, intermediate density lipoprotein; idlfcpct_p, postprandial free cholesterol in IDL to total lipids in IDL; lafa_f, fasting ratio of linoleic acid to total fatty acids; lafa_p, postprandial ratio of linoleic acid to total fatty acids; leu_f, fasting leucine; lhdlc_p, postprandial total cholesterol in large HDL; lhdlfc_p, postprandial free cholesterol in large HDL; lhdlcepct_p, postprandial cholesterol esters in large HDL to total lipids in large HDL; lhdlcpct_p, postprandial total cholesterol in large HDL to total lipids in large HDL; lhdlfcpct_p, postprandial free cholesterol in large HDL to total lipids in large HDL; MR, Mendelian randomization; mvldlplpct_p, postprandial phospholipids in medium VLDL to total lipids in meduim VLDL; mvldlplpct_r, response phospholipids in medium VLDL to total lipids in medium VLDL ratio; NEO, Netherlands Epidemiology of Obesity Study; pufafa_f, fasting ratio of polyunsaturated fatty acids to total fatty acids; pufafa_p, postprandial ratio of ployunsaturated fatty acids to total fatty acids; svldlfcpct_r, response free cholesterol in small VLDL to total lipids in small VLDL ratio; VLDL, very low density lipoprotein.

#### MR results—fasting

A total of 87 fasting traits exhibited nominal evidence of an association with BMI in the general population, 5 remained after correction for multiple testing (Table 2, Supplemental Table 4, Figure 6). The strongest association observed was with the ratio of linoleic acid to total fatty acids (lafa; β: −0.106; SE: 0.025; *P* = 1.80 × 10^−5^). This was followed closely by 4 other inverse associations: the ratio polyunsaturated fatty acids (pufafa; β: −0.081; SE: 0.024; *P* = 6.80 × 10^−4^) to total fatty acids, the ratio ω-6 to total fatty acids (faw6fa; β: −0.081; SE: 0.024; *P* = 6.97 × 10^−4^), and the abundance of citrate (β:−0.079; SE: 0.024; *P* = 8.63 × 10^−4^). In addition, 1 fasting metabolite showed evidence of a positive association with BMI, the BCAA leucine (β: 0.068; SE: 0.020; *P* = 5.62 × 10^−4^). If data are summarized by metabolite annotation class (Supplemental Table 1), we find that the classes with the largest mean absolute BMI effect are ketone bodies (mean β: −0.047, *n* = 2), amino acids (0.036, *n* = 8), inflammation (0.029, *n* = 1), glycerides and phospholipids (0.027, *n* = 9), and lipoprotein subclasses (0.023, *n* = 98). Those with the smallest mean absolute effect are lipoprotein particle size (0.009, *n* = 3), lipoprotein subclass ratios (0.003, *n* = 70), and cholesterol (0.002, *n* = 9) (Supplemental Figure 11).

#### MR results—postprandial

A total of 96 postprandial metabolites exhibited nominal evidence of an association with BMI, 12 remained after correcting for multiple testing (Table 2, Supplemental Table 4, Figure 6). Like the fasting metabolite traits, the fatty acid ratios linoleic acid (lafa; β: −0.092; SE: 0.025; *P* = 2.0 × 10^−4^), ω-6 (faw6fa; β: −0.080; SE: 0.024; *P* = 7.83 × 10^−4^), polyunsaturated fatty acids (pufafa; β: −0.077; SE: 0.024; *P* = 1.10 × 10^−3^), and citrate (β:−0.105; SE: 0.022; *P* = 2.83 × 10^−6^), which had the largest effect, each also had an inverse relationship with BMI (FIGURE 5, FIGURE 6). In addition, the amino acid histidine, large HDL cholesterol, and free cholesterol decreased with increases in BMI. Furthermore, the ratio of cholesterol, cholesterol esters, and free cholesterol to total lipids in HDL decreased with increases in BMI. Finally, the ratio of free cholesterol to total lipids in IDL and the ratio of phospholipids to total lipids in medium VLDL decreased with increases in BMI. If data are summarized by metabolite annotation class (Supplemental Table 1), we find that the classes with the largest mean absolute BMI effect are glycolysis-related metabolites (mean β: −0.046, *n* = 3), inflammation (0.40, *n* = 1), and lipoprotein subclasses (0.02, *n* = 98). The mean effect among amino acids is not negative (−0.019, *n* = 8) in opposition to that seen in fasting data (Supplemental Figure 11).

#### MR results—response

A total of 18 response metabolites exhibited nominal evidence of an association with BMI, 3 remained after correcting for multiple testing (Table 2, Supplemental Table 4, Figure 6). The largest effect was observed for the amino acid alanine (β: −0.081; SE: 0.023; *P* = 5.91 × 10^−4^). Data suggested that increases in BMI attenuates the increase in abundance of the amino acid alanine after a liquid mixed meal (FIGURE 5, FIGURE 6)—consistent with earlier observational analyses (Figure 4). The other 2 response traits that associated with BMI were the ratio of phospholipids in medium VLDL to total lipids in medium VLDL (β: 0.091; SE: 0.027; *P* = 6.75 × 10^−4^) and the ratio of free cholesterol in small VLDL to total lipids in small VLDL (β: 0.082; SE: 0.025; *P* = 1.11 × 10^−3^). As a class, amino acids (mean β: −0.059, *n* = 8) had the largest mean BMI response effect estimate (Supplemental Figure 11). This was followed by the ketone bodies acetate and β-hydroxybutyrate (mean β: 0.048, *n* = 2) and the glycolysis-related metabolites citrate, glucose, and lactate (mean β: −0.042, *n* = 3).

### Subpopulation and sensitivity analyses

All observational and MR analyses were repeated in each of the 2 subpopulations and in the NEO cohort without the inclusion of weights. These analyses allowed us to evaluate the variability in effect estimates measured in a random (Leiderdorp) and biased (Leiden) population sample as and the effectiveness of the weights in our primary analyses (wNEO). We found that effect estimates from the Leiderdorp and the wNEO frameworks are strongly correlated (observational Pearson *r* = 0.983; MR = 0.855) and correlation coefficients weakened when compared with the unweighted NEO and the Leiden frameworks (Supplemental Figure 12). Decreases in agreement between analyses as the sample population mean BMI shifts would indicate that there are either unaccounted confounders influencing the results or that the relationship between BMI and metabolite trait variation is not always linear.

To evaluate the influence of confounders on MR effect estimates, we reran the association analysis in the wNEO data set. Three additional covariates were included in the model (smoking, on a weight loss diet, and principal component 3) and 101 sample with a peroxidation sample quality flag, a sample quality metric identified by Metabolon, were removed. Overall primary (wNEO) observational (Pearson *r* = 0.998) and MR (Pearson *r* = 0.963) effect estimates correlated strongly with those from the sensitivity analysis (Supplemental Table 5). A more comprehensive description of these analyses is in Supplemental Results along with illustrations in Supplemental Figures 13–15.

### Comparison with previous work

We compared our observational effect estimates with 57 matching fasting metabolites measured using the same platform, in a young adult cohort [15]. Overall effect estimates correlated well (Pearson *r* = 0.82) with the caveat that estimates cannot be directly compared given differences in data transformations between the studies. However, there are 37 shared associations, 6 unique to this study, 12 unique to the other, and 2 yielding no association in either study (Figure 3B). Five of the shared associations were different across studies (total cholesterol, sphingomyelins, phosphatidylcholines, phosphoglycerides, and linoleic acid). Among the 12 metabolites unique to existing work before that presented in this study, are 10 metabolites that previously exhibited a strong positive effect but showed no evidence for an association or negative point estimates (total lipids in IDL, large LDL, medium LDL and small LDL, total cholesterol in IDL and LDL, and 4 fatty acid metabolites ω-3 and ω-6, polyunsaturated fatty acids, and docosahexaenoic acid). These differences may be the product of nonlinear associations between these metabolites and BMI in combination with age effects and differences in the distribution of BMI between these studies. Despite these difference, 57 fasting MR estimates that did overlap with previous work showed strong correlation between the 2 studies (Pearson *r* = 0.862; *P* = 7.99 × 10^−18^) (Supplemental Figure 16), with 1 shared fasting MR association—the amino acid leucine.

### Atherogenic lipoprotein profile

**FIGURE 7:**
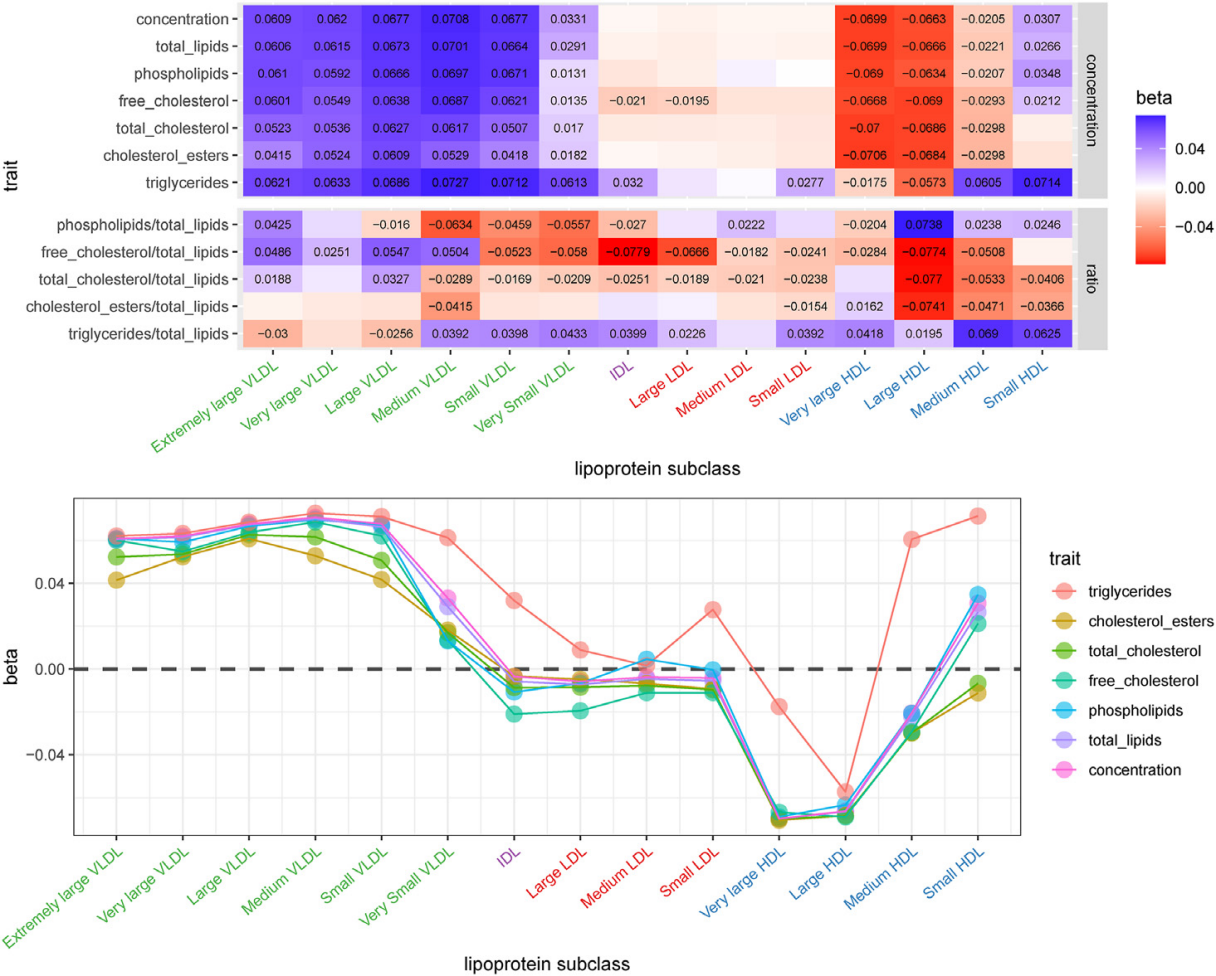
Fasting lipoprotein cross-sectional profile. (Upper) A tile plot of cross-sectional effect estimates for lipoproteins and lipoprotein ratios in the fasting state. Tiles with an effect estimate provided in text are those associated with BMI (*P* < 1.16 × 10^−3^). The lipoproteins are organized by size or density along the x-axis, and the component or ratio being measured is along the y-axis. (Lower) A dot plot or profile of cross-sectional effect estimates for lipoproteins (x-axis) ordered by lipoprotein size or density with effect estimates (y-axis). The component or measurement of each lipoprotein is defined by the color as described in the key. All results can be found in Supplemental Table 4. HDL, high density lipoprotein; IDL, intermediate density lipoprotein; LDL, low density lipoprotein; VLDL, very low density lipoprotein.

Given the prevalence of lipoproteins and their lipids on this platform, we looked for the expected atherogenic lipoprotein profile—that is decreases in HDL and increases in non-HDL lipoproteins with increase in BMI [[43], [44], [45], [46]]. Both observational and MR associations between BMI and fasting and postprandial dietary states do suggest a worsening atherogenic lipoprotein profile with BMI (Figure 7, Supplemental Figures 17 and 18). As illustrated with the fasting observational effect estimates in Figure 7, the atherogenic VLDL lipoproteins are higher with increases in BMI whereas nonatherogenic lipoproteins—or those involved in the reverse cholesterol transport system (HDL), were lower. In contrast to expectations, other atherogenic lipoproteins IDL and LDL, largely do not associate with BMI. In addition, triglycerides in nonatherogenic medium HDL and small HDL increase with increases in BMI. Furthermore, as HDL lipoprotein density increases, the inverse relationship with BMI weakens, and as seen with small HDL, the association becomes positive (Figure 7). This is consistent with the aforementioned observation that the strongest inverse association with BMI is that with mean diameter of HDL particles, suggesting that as BMI increases there is an associated shift toward smaller HDL particle size. Along with these changes, other observations were consistent with obesity influencing lipid profiles that are associated with cardiometabolic disease [14,22,47]. These included increases in apolipoprotein B, decreases in apolipoprotein A-1, and increases in the ratio of B to A-1 with increases in BMI, in both the fasting and postprandial states [45,48].

## Discussion

This study provided effect estimates for observational and MR associations between BMI and metabolites in a *1*) fasted, *2*) postprandial, and *3*) response state, using individual-level data from a middle-aged cohort of 5517 individuals of Northern European ancestry including a liquid mixed meal challenge. Each dietary state provides a unique examination at metabolite variation, the value of which are actively being explored. Broad observational associations were observed between BMI and metabolites (69% of tested traits). In addition, MR estimates were largely concordant with the observational estimates (Figure 3D). This is consistent with previous research and suggests that, at least for this lipidomic platform, observational estimates are reasonably well aligned to MR effect estimates [15]. This generalization holds less true for response traits than for postprandial and fasting trait (Figure 3D). After correcting for multiple testing, 20 metabolite traits maintain evidence of a causal BMI effect (Figure 6). This includes the inverse association with fasting and postprandial ratios of linoleic acid (lafa), ω-6 (faw6fa), and polyunsaturated fatty acids (pufafa) to total fatty acids and fasting and postprandial citrate abundance. In addition, the amino acid leucine has a positive MR association with BMI in the fasting state, histidine has an inverse MR association with BMI in the postprandial state, and alanine has an inverse MR association with BMI in the response state.

The MR association of BMI across BCAAs and aromatic amino acids (AAAs) were similar with positive effects in the fasting state, no effect in the postprandial state, and an inverse association in the response state (FIGURE 2, FIGURE 5). Meanwhile, the dietary profile of (glucogenic) amino acids suggests no effect in the fasting state and inverse effects in both the postprandial and response states. These observations may suggest that BMI may have a causal influence on the synthesis or metabolism of amino acids in general, particularly regarding their inverse association with BMI in the response state (FIGURE 2, FIGURE 5). BCAAs and AAAs have long been associated with obesity, glucose, insulin (resistance), and type 2 diabetes [[49], [50], [51], [52], [53], [54], [55]]. In support of early observations, numerous recent studies that have shown associations between amino acids and BMI, visceral adipose tissue and weight change [15,[56], [57], [58], [59]], between BCAAs and AAAs with insulin resistance and type 2 diabetes [[60], [61], [62], [63]], used MR to show that insulin resistance increase BCAAs concentration [64] and used a randomized control trail to show that restricting BCAAs can improve glucose tolerance and reduce fat accumulation [65]. Indeed evidence now suggests a potential causal pathway from BMI to type 2 diabetes through the intermediate traits of insulin resistance and BCAAs, respectively [64]. Observations in this study reinforce the causal association between BMI and amino acids, with the additional observations that postprandial abundance of the glucogenic amino acids decreases with BMI and that the relative change in amino acid abundance in response to a liquid mixed meal decreases with increases in BMI.

As the study was centered in the Netherlands, study samples were limited to individuals of Northern European ancestry, and as such, inferences made from results should be limited to populations of similar ancestry and environment. Sampling was limited to individuals of middle age (range 44 to 66 y and a mean of 56 y) (Table 1). Although this provides specificity to a middle age population it also limits the results to this population age range as well. The NEO population sampling was performed in 2 distinct batches, one focused on oversampling individuals of high BMI (Leiden) and the second, a random, and representative sampling of the population (Leiderdorp). The product of this led to not only the use of weighted linear regression analyses to maintain sample size and power but also providing estimates that are representative of the study population at large. The validity of this assumption rests in the credibility of the weights, which were specifically designed to make the BMI distribution of the Leiden sample mirror that of the Leiderdorp sample [42]. We have illustrated through comparison with the Leiderdorp sample that effect estimates are consistent across the 2 analyses, imparting support to the weighted analysis (Supplemental Figure 12). With these factors, there was just a single postprandial time point evaluated in this study limiting the inferences that can be drawn. It is reasonable to speculate that an earlier time point or a complete time series would provide additional information about how BMI might influence postprandial variation. Finally, although a single complex meal standardizes the complexities in evaluating metabolite response and postprandial abundances alternative meals could provide novel insights in the interplay among BMI, nutrition, and metabolite variation.

In conclusion, using a middle-aged cohort of 5517 individuals, we derived effect estimates for BMI on metabolite traits in the fasting, postprandial, and response dietary states. We were able to show that results are broadly correlated between observational and one-sample MR analyses and in the context of the numerous documented associations among BMI, metabolites, and disease, this gives support to a conclusion that metabolites may act as intermediates—or are at least biomarkers of underlying driver physiology—between adiposity accumulation and disease. Furthermore, this work suggested that the dynamic metabolome may potentially flag common biological events, which will systematically vary by BMI, which may be linked to the etiology of disease, and which are linked to life course events such as feeding and metabolic response.

## Acknowledgments

We thank Pat van Beelen for data collection, Petra Noordijk for laboratory management, and Ingeborg de Jonge for data management of the NEO study. We thank Nutricia Research, Utrecht, The Netherlands, for providing the liquid mixed meal.

## Author contributions

The authors’ responsibilities were as follows – DAH, RL-G, CJB, DOM-K, KWvD, NJT: designed the study; FRR, RdM: designed and conducted the NEO study and managed the data; DAH, NJT: analyzed the data; DAH, RL-G, KWvD, NJT: wrote the manuscript; DAH, NJT: had primary responsibility for final content; and all authors: read and approved the final manuscript.

### Conflict of interest

Ruifang Li-Gao is a part-time contractor of Metabolon Inc.

## Funding

This research was supported by the Netherlands Cardiovascular Research Initiative: an initiative with support from the Dutch Heart Foundation (CVON2014-02 ENERGISE). NJT is a Wellcome Trust Investigator (202802/Z/16/Z); is the PI of the Avon Longitudinal Study of Parents and Children (MRC & WT 102215/2/13/2) is supported by the University of Bristol NIHR Biomedical Research Centre (BRC-1215-20011), the MRC Integrative Epidemiology Unit (MC_UU_12013/3); and works within the CRUK Integrative Cancer Epidemiology Programme (C18281/A19169). DAH was supported by NJT’s Wellcome Investigator Award (202802/Z/16/Z) and along with CJB was also supported by the University of Bristol and UK Medical Research Council (MC_UU_00011/1 and MC_UU_00011/6). RL-G was supported by JPI HDHL-DIYUFOOD project. DOM-K is supported by the Dutch Science Organization (ZonMW-VENI Grant 916.14.023).

## Data availability

Data described in the manuscript are available to researchers according to the NEO research procedure. Contact the scientific director of the NEO study for access. All analytic codes are publicly and freely available without restriction at the github repository (https://github.com/hughesevoanth/NEO_BMI_Metabolite_MR).
