# Supplementary material for: The association between body mass index and metabolite response to a liquid mixed meal challenge: a Mendelian randomization study
Source: Am J Clin Nutr. 2024 Mar 16;119(5):1354–70. doi: 10.1016/j.ajcnut.2024.03.009 (PMC11130664; doi:10.1016/j.ajcnut.2024.03.009)
Supplement: Multimedia component 1 [file mmc1.pdf]

Supplementary Materials for:

The association between body mass index and metabolite response to a liquid mixed meal challenge: a Mendelian randomization study

David A. Hughes, Ruifang Li-Gao, Caroline J. Bull, Renée de Mutsert, Frits R. Rosendaal, Dennis O. Mook-Kanamori, Ko Willems van Dijk, Nicholas J. Timpson

Table of Contents

1. Annotation of Supplementary Tables pages 1-2
2. Supplementary Methods pages 2-4
3. Supplementary Results pages 4-6
4. Supplementary Figure Legends pages 6-14
5. Supplementary Figures pages 15-32
6. metaboprep log file pages 33-35
7. metaboprep report pdf pages 36-46

Annotation of Supplementary Tables

1. Supplementary Table 1
    a. metabolite annotation
2. Supplementary Table 2
    a. annotation of other study (co)variables
3. Supplementary Table 3
    a. observational and MR summary statistics for alternative response traits
4. Supplementary Table 4
    a. primary study results – observational and MR summary statistics for the wNEO framework
5. Supplementary Table 5
    a. observational and MR summary statistics for the wNEO sensitivity (additional covariables or confounders) analyses
6. Supplementary Table 6
    a. observational and MR summary statistics for the wNEO framework but with no transformation of the outcome (metabolite) traits
7. Supplementary Table 7
    a. observational and MR summary statistics for NEO cohort but with no weights
8. Supplementary Table 8
    a. observational and MR summary statistics for the Leiderdorp NEO sub-sample
9. Supplementary Table 9
    a. observational and MR summary statistics for the Leiden NEO sub-sample
10. Supplementary Table 10

    a. observational and MR summary statistics for females using the wNEO framework
11. Supplementary Table 11
    a. observational and MR summary statistics for males using the wNEO framework
12. Supplementary Table 12
    a. observational and MR summary statistics for females in the Leiderdorp sub-sample
13. Supplementary Table 13
    a. observational and MR summary statistics for males in the Leiderdorp sub-sample
14. Supplementary Table 14
    a. fasting -v- postprandial metabolite paired t-tests
15. Supplementary Table 15
    a. associations and variance explained between study variables (Table 2) and BMI and the PGS

Supplementary Methods

Metabolite data quality control

The initial NEO metabolite data set contained data for 5,744 individuals, 229 fasting metabolite, 229 postprandial metabolites, and 148 previously derived (38) ornls response metabolite traits. We used the R package metaboprep to perform quality control on the data prior to analysis. The following parameter values were used when running metaboprep: (1) feature missingness 0.2, (2) sample missingness 0.2, (3) total peak area standard deviation 5, (4) principal component (PC) outlier standard deviations 5, (5) tree cut height 0.5, (6) derived variable exclusion FALSE. Metaboprep performs an initial exclusion of samples with missingness greater than or equal to 80% (n = 9), followed by exclusion of features with missingness greater than equal to 80% (n = 0). After this initial filtering, the data set is filtered using the parameters defined above. This will filter (1) samples (n = 217) and then (2) features (n = 3) with greater than or equal to 20% missingness, (3) then filter samples with a total sum abundance (TSA) at complete features (no missingness) that fall beyond 5 standard deviations from the mean of the observed TSA distribution (n = 0), and then (4) filter samples (n = 0) that are 5 standard deviations from the mean of the first 'i' informative PCs, here defined by the Cattel's Scree test acceleration factor (i = 2). Parameter 5 defines the clustering dendrogram tree cut height which was used to estimate the number of clusters, and then used to identify the representative or principal variables in the data set. These principal variables were used in the data filter PC analysis used by parameter 4 above. Derived variables or features such as ratios that are derived from multiple features in the data set were (6) not excluded from any analyses. Given the defined *metaboprep* parameters 226 samples and 3 metabolites, all ornls response traits, were filtered from the data set. The *metaboprep* log file

and *metaboprep* report, as generated by *metaboprep*, are available at the end of this **Supplementary Materials** document.

Following metaboprep quality control, additional quality control steps were taken. First, all zero values were turned into NAs. Second, for each metabolite (in the fasting and postprandial state, individually) any sample with a value 10 interquartile distances from the median was turned into NAs. This QC-step removes all extreme observations that are unrealistic given the empirical distribution of a single metabolite trait. Third, for each metabolite we used the expectation that fasting and postprandial data are correlated to estimate a delta value (postprandial - fasting), from which all samples that fell five interquartile distances from the median of that metabolite's delta distribution were turned into NAs - in both the fasting and postprandial data. This QC-step removes all extreme observations that are unrealistic given the observed, empirical, correlated nature of this bivariate data. See **Supplementary Figure 3** for an example illustration of these QC steps.

Effective number of tested metabolites

There are 687 metabolite traits in the data set (fasting = 229, postprandial = 229, response = 229) but these traits are not strictly independent. First, given the presence of both fasting and postprandial data, and a response trait derived from the two, we would expect there to be a degree of dependency. Second, given the focus on lipids and lipoproteins on this platform we also expect an abundance of inter-correlated structure (**Supplementary Figure 4**). As such we used the R package *iPVs* (https://github.com/hughesevoanth/iPVs) to estimate the effective number of metabolites in the data set. The effective number represents an estimate of the number of representative or independent traits present in the data (47–49). The method implemented by iPVs provides an estimate by the iterative construction of a hierarchical clustering dendrogram followed by a tree cut to identify clusters of correlated variables and identification of a principal variable (PVs) for each cluster. The iPVs() function parameters were set to: "spearman" for the correlation matrix, "R" for the distance matrix, "complete" for the hierarchical cluster method, and 0.5 for the tree cut height. The distance matrix produced is equal to one minus the absolute Spearman's rho. At a tree cut height of 0.5, metabolites with a Spearman's rho greater than 0.5 cluster together. If the tree cut height was, for example, set to a value of 0.8, then metabolites with a Spearman's rho greater than 0.2 would cluster together. In total 43 clusters or representative variables in the NEO metabolite data set

were identified. Resultantly our data reduced, study-wide Bonferroni (BF) corrected p-value was set to 0.05/43 or 1.163×10^{-3}.

Identification of possible confounders

To identify possible confounders, or variables that may violate MR IV assumption number two (independence) we tested, in a univariate fashion, for an association between (a) sampling date and BMI and BMI-PGS, (b) study covariables and BMI, (c) study covariables and BMI-PGS and (d) study covariables and metabolite traits (**Supplementary Table 15**). An ANOVA analysis of the univariate linear models was then used to partition the sums of squares and estimate the variance explained, in the form of an eta-squared statistic (η^2). Eta-squared (η^2) is a measure of the variance explained and can be derived from sums of squares or deviances extracted from ANOVAs. In addition, an ANOVA F-test was used to estimate a p-value for each association. These analyses were carried out for the NEO cohort as a whole and for each of the two NEO sub-samples and provides a means to identify possible confounders that may invalidate the MR framework.

Supplementary Results

Possible confounders

We performed a complete assessment of the association between BMI and BMI-PGS with all study covariables to identify those variables that could be possible confounders in association analyses. All covariables were chosen for inclusion because of a potential correlation with BMI and metabolite trait variation. Indeed, we observed that 60 of the tested 91 covariables are associated with BMI (wNEO, $P < 9.26 \times 10^{-4}$; **Supplementary Table 15** and **Supplementary Figure 8**). Those with the strongest association with BMI are the study sampling variables visit date and sub-population (already included as model covariates), followed by the obesity adjacent traits weight and hip and waist circumference, and then basal metabolic rate, glucose metabolism, resting energy expenditure (kcal/day), mean oxygen production (VO2, ml/min) and mean carbon dioxide production (VCO2, ml/min). In addition, smoking as measured by packyears associates with BMI, explaining 3.2% (η^2) of the variation in BMI (wNEO, ANOVA F-test $P = 1.28 \times 10^{-37}$), as does education (edu_level, $\eta^2 = 0.044$, P

= $1.96x10^{-47}$), and household income (income_hh, $\eta^2 = 0.015$, P = $1.52x10^{-14}$, **Supplementary Table 15**).

In contrast, the MR instrumental variable (BMI-PGS) is associated with 17 covariables (wNEO, P < $9.26x10^{-4}$, **Supplementary Figure 9**), five of which are obesity adjacent traits, three are sampling and sub-population structure variables, three smoking variables, 4 diet variables, a calorimetry variable, and a sample quality variable. They are, in order of association strength: hip circumference ($\eta^2 = 0.039$, P = $2.05x10^{-49}$), weight ($\eta^2 = 0.038$, P = $9.97x10^{-49}$), waist circumference ($\eta^2 = 0.035$, P = $1.76x10^{-44}$), sub-population ($\eta^2 = 0.020$, P = $3.61x10^{-26}$), basal metabolic rate ($\eta^2 = 0.016$, P = $1.00x10^{-20}$), waist-to-hip ratio ($\eta^2 = 0.011$, P = $7.43x10^{-15}$), visit date ($\eta^2 = 0.029$, P = $1.56x10^{-14}$), principal component 3 ($\eta^2 = 0.006$, P = $5.03x10^{-09}$), smoking packyears ($\eta^2 = 0.006$, P = $4.16x10^{-08}$), on a weight loss diet ($\eta^2 = 0.004$, P = $6.40x10^{-07}$), imputed smoking packyears ($\eta^2 = 0.005$, P = $7.63x10^{-07}$), type of diet ($\eta^2 = 0.007$, P = $1.24x10^{-06}$), on a diet last month (yes|sometimes|no, $\eta^2 = 0.004$, P = $8.04x10^{-06}$), smoker (never|former|current, $\eta^2 = 0.004$, P = $1.01x10^{-05}$), food rule description ($\eta^2 = 0.037$, P = $9.60x10^{-05}$), mean CO_2 production ($\eta^2 = 0.010$, P = $6.04x10^{-04}$), and the sample quality flag for signs of peroxidation ($\eta^2 = 0.002$, P = $8.30x10^{-04}$, **Supplementary Table 15**). The association between BMI-PGS and principal component three is partially driven by its association with sub-population structure ($\eta^2 = 0.032$, P = $1.71x10^{-41}$, **Supplementary Figure 13**). All 17 covariables associated the BMI-PGS were also tested for an association with each metabolite trait in a univariable linear model. Results illustrated broad association across all traits and covariables, defining each covariable as a confounder in MR analysis (**Supplementary Figure 10**).

Sub-population analyses

All observational and MR analyses were repeated in each of the two sub-populations and in the NEO cohort without the inclusion of weights. The (i) randomly sampled Leiderdorp sub-population (n=1406) provides a means to verify the point estimates derived from the primary weighted NEO (wNEO) results presented above. The (ii) unweighted NEO cohort provides an evaluation of the weights, and the (iii) Leiden sub-population sample (n=4111) provides an evaluation of effect estimates derived from a sampling biased for elevated BMI.

First, observational effect estimates from the wNEO analysis strongly correlate with those from the Leiderdorp sub-sample, with a Pearson's r of 0.983. The correlation does reduce when compared to the un-weighted NEO analysis (r = 0.96) and the biased Leiden sample (r = 0.91, **Supplementary Figure 12**). Second, MR effect estimates remain strongly correlated between the wNEO and Liederdorp sample (r = 0.855), but as the shift in sample population BMI increases the correlation reduces. When compared to the un-weighted NEO sample the Pearson's r is 0.445 (P = $1.10x10^{-34}$) and even becomes negative when compared to the Leiden sample (Pearson's r = -0.107, P = $4.84x10^{-03}$; **Supplementary Figure 12**). The comparison to the randomly sample Leiderdorp sample would suggest that the weights used in the wNEO analysis did not introduce any strong error in effect estimates. Yet, the decrease in congruency between analyses as the sample population mean BMI shifts would indicate that there are either un-accounted for confounders influencing the results or that the relationship between BMI and metabolite trait variation are not linear.

Sensitivity analyses

To evaluate the influence of confounders on MR effect estimates we reran the association analysis in the wNEO data set. We assumed that the association between BMI-PGS and hip circumference, weight, waist circumference, basal metabolic rate, waist-to-hip ratio, and mean CO^2 production were the product of biological similarities with our exposure of interest, BMI, and not confounders. We did include a smoking variable (packyears), a diet variable (on a weight loss diet), and PC3 as new covariates in the MR analysis. We also excluded the 101 samples with a peroxidation flag on their samples. Overall primary (wNEO) observational (Pearson's r = 0.998) and MR (Pearson's r = 0.963) effect estimates correlate strongly with those from the sensitivity analysis (**Supplementary Table 5**). Further no effect estimates differ between the primary and sensitivity models (z-test, P< 0.05) indicating that the inclusion of the additional confounders had minimal effect on the association statistics (**Supplementary Figure 14** and **15**).

Supplementary Figure Legends

Supplementary Figure 1: Distribution of BMI by sub-population. Histograms of the distribution of BMI in the Leiderdorp (red, top left) and the Leiden (blue, top right) subpopulations, and compared to each other (lower) in a density plot.

Supplementary Figure 2: BMI and PGS by visit date. Box-plots of NEO sampling dates (month-year) along the x-axis with the mean (box vertical line), 1st and 3rd quartile (box) and 1.5*IQR of box (whiskers) illustrated. The red vertical line illustrates the mean value of the total sample. Upper plot illustrates variation in BMI. The lower plot illustrates the variation in the instrumental variable – BMI-PGS. Defined in each sub-header is the proportion of variation in BMI (upper) or BMI-PGS (lower) explained by sample date.

Supplementary Figure 3: Raw and QC'd fasting and postprandial scatter plots for S-HDL-C. (***Left***) A scatter plot for fasting (x-axis) and postprandaial (y-axis) cholesterol in small HDL (S-HDL-C). An equivalency line (x = y) is illustrated as a dashed grey line. Each dot is a sample, those colored blue had a zero value and were turned into NA, those colored green were identified as outliers (10 interquartile distances from the median) in one of the two dietary states and turned into NA in that dietary state, those colored red were identified as delta outliers (5 interquartile distances from the median). Delta was estimates as the postprandial value minus the fasting value. Those samples colored as grey were not altered. (***Right***) is a scatter plot for fasting (x-axis) and postprandaial (y-axis) total lipids in small HDL (S-HDL-L) after quality control. An an equivalency line (x = y) is illustrated as a dashed grey line. In addition, six linear models were fit to the data and are plotted. They are a (black) a Deming regression, (orange) a linear model, (blue) a linear model were the independent term or fasting data was fit as a quadratic term, (purple) a linear model where the fasting data was fit as a cubic term, (red) a median regression, and (green) a generalized additive model or GAM where the fasting data was fit as a smooth. Each of these models were fit to evaluate how much variability was present among each model type.

Supplementary Figure 4: Metabolite and covariable clustering dendrogram. Hierarchical clustering dendrogram of metabolites (top) and study covariables (bottom). Leafs colored blue indicate principal variables, as identified by the iPVs R package. The red horizontal line, at a height of 0.5, marks the tree cut height used to identify clusters by iPVs. The y-axis is equivalent to 1- absolute Spearman's rho.

Supplementary Figure 5: Mean lipid estimates across lipoproteins. For each lipoprotein (x-axis), organized by density or size (most dense to least dense – left to right) the mean (units = mmol/l) of each lipid fraction was estimated and plotted (y-axis). Each lipid fraction is plotted in a different color as indicated in the legend.

Supplementary Figure 6: Postprandial-Fasting delta distributions. A simple delta value (postprandial - fasting) was estimated for each sample by metabolite, after centring (mean=0) and scaling (SD=1) the fasting and postprandial data together. Plotted here, for each metabolite (x-axis) is the mean delta (point) and the 95% confidence intervals (whiskers) of the delta distribution (y-axis). The y-axis are on a fixed scale across all three plot rows. The top row illustrates values for metabolites partitioned and coloured by their respective class. The middle and bottom row illustrate the lipoproteins and lipoprotein ratios, respectively, coloured by their subclass or lipoproteins – organized from least dense to most dense, left to right. Plot values can be found in **Supplementary Table 14**.

Supplementary Figure 7: Observational effect estimates by dietary state and class. (A) BMI – metabolite effect estimates and their relationship with the linear model P-value (-log10(P)) are illustrated in the volcano plots, one for each dietary state (fasting, postprandial,

and response). Along the x-axis are the point or effect estimate and along the y-axis are the -log10(P-values). Metabolites are classified and colored by Nightingale Health class assignments. The alpha threshold for defining a metabolite as associated with BMI is indicated by the horizontal black line ($P<0.05/40$). **(B)** BMI – metabolite observational effect estimates are classified by dietary state (fasting, postprandial and response) and Nightingale Health class assignments (x-axis and color key) to illustrate the distribution and mean effect for each class of metabolite by dietary state. Each box defines the mean (box horizontal line), the 25th and 75th percentile of the data distribution (limits of the box), and the 1.5 * inter-quartile range of the box (whiskers) as defined by the ggplot function geom_boxplot().

Supplementary Figure 8: Variance explained in BMI by associated covariables. The x-axis provides an (weighted; wNEO) estimate of the variance explained (eta-squared) in BMI by each study covariable as derived by extracting the sums of squares from an analysis of variance. The y-axis provides the name of all covariables that associate with BMI. Covariable names can be mapped to descriptions in **Supplementary Table 2**. Study covariables were placed into categories (color key) and used to color each bar, which are also available in **Supplementary Table 2**. Plot values can be found in **Supplementary Table 15**. **(B)** For each (sub-)population (plot columns) analysis (1) wNEO, (2) Leiderdorp, (3) NEO, and (4) Leiden univariable linear models and analysis of variances (ANOVA) were run with either BMI or BMI-PGS (x-axis) set as the dependent and each covariable (y-axis) was defined as the independent. From each ANOVA the sums of squares were extracted, and an estimate of the variance explained in the dependent was derived in the form of an eta-squared (η^2) statistic (blue shading and cell text (η^2*100)). For each analysis where an association was observed ($P<0.05/40$) a red dot (scaled by -log10(ANOVA F-test P)) can be found in the relevant cell. The y-axis is ordered by the wNEO BMI η^2.

Supplementary Figure 9: Variance explained in BMI-PGS by associated study covariables. (A) The x-axis provides an (weighted; wNEO) estimate of the variance explained (eta-squared) in BMI-PGS by each study covariable as derived by extracting the sums of squares from an analysis of variance. The y-axis provides the name of all covariables that associate with BMI. Covariable names can be mapped to descriptions in **Supplementary Table 2**. Study covariables were placed into categories (color key) and used to color each bar, which are also available in **Supplementary Table 2**. Plot values can be found in **Supplementary Table 15**. **(B)** For each (sub-)population (plot columns) analysis (1) wNEO, (2) Leiderdorp, (3) NEO, and (4) Leiden univariable linear models and analysis of variances (ANOVA) were run with BMI-PGS (x-axis) set as the dependent and each covariable (y-axis) associated with BMI-PGS in the wNEO analysis was defined as the independent. From each ANOVA the sums of squares were extracted, and an estimate of the variance explained in the dependent was derived in the form of an eta-squared (η^2) statistic (blue shading and cell text (η^2*100)). For each analysis where an association was observed ($P<0.05/40$) a red dot (scaled by -log10(ANOVA F-test P)) can be found in the relevant cell. The y-axis is ordered by the wNEO p-value.

Supplementary Figure 10: Metabolite associations with "BMI-PGS associated" covariables. For each metabolite (x-axis) a univariable linear models and analysis of variances (ANOVA) were run against each covariable (plus alcohol and higher education) previously observed to be associated with BMI-PGS in univariable analyses (y-axis). Each metabolite trait was set as the dependent and each covariable was set as the independent variable. From each ANOVA the sums of squares were extracted, and an estimate of the variance explained in the dependent was derived in the form of an eta-squared (η^2) statistic

(blue shading). For each analysis where an association was observed ($P<0.05/40$) a red dot (scaled by -log10(ANOVA F-test P)) can be found in the relevant cell. The y-axis is ordered by the average η^2 across all metabolites.

Supplementary Figure 11: MR effect estimates by dietary state and class. (A) BMI – metabolite MR effect estimates and their relationship with the linear model P-value (-log10(P)) are illustrated in the volcano plots, one for each dietary state (response, postprandial and fasting). Along the x-axis are the point or effect estimate and along the y-axis are the -log10(P-values). Metabolites are classified and colored by Nightingale Health class assignments. The alpha threshold for defining a metabolite as nominally associated ($P<0.05$) is the dotted grey line, and associated with BMI is indicated by the horizontal black line ($P<0.05/43$). **(B)** BMI – metabolite MR effect estimates are classified by dietary state (response, postprandial, and fasting) and Nightingale Health class assignments (x-axis and color key) to illustrate the distribution and mean effect for each class of metabolite by dietary state. Each box defines the mean (box horizontal line), the 25th and 75th percentile of the data distribution (limits of the box), and the 1.5 * inter-quartile range of the box (whiskers) as defined by the ggplot function geom_boxplot().

Supplementary Figure 12: Scatter plot of Obs. & MR effect estimates by(sub)-population. Observational (top) and MR (bottom) effect estimates are compared between the weighted NEO (wNEO; x-axis) analyses and each other sample (sub)-population (y-axis) – left: Leiderdorp, middle: NEO, right: Leiden. Plotting dots in red, blue, and green denote effect estimates for metabolites in the response, postprandial, and fasting dietary states, respectively. Solid circles represent effect estimates that differ, as test by a z-test, between the two (sub-)sample populations at a p-value of 0.05. Solid squares are those that differ at a

Bonferroni adjusted p-value of 0.05/603. The thin black line represents an equivalency line (intercept = 0, slope = 1) and the thick black line is the best fit line through the data. An estimate of Pearson's r correlation coefficient is in the lower right corner of each plot. All effect estimates for the wNEO, Leiderdorp, NEO, and Leiden analysis (sub)-population frameworks can be found in **Supplementary Tables 4, 8, 7,** and **9**, respectively.

Supplementary Figure 13: Sub-population association with PC3. A scatter plot of NEO study samples on principal component three and four, as provided as covariables by NEO data managers. Each dot is an individual that is colored by the sample sub-population Leiden (red) and Leiderdorp (blue). PCs one (η^2 = 0.002, P = 9.33x10^{-04}), three (η^2 = 0.032, P = 1.71x10^{-41}), and four (η^2 = 0.006, P = 3.16x10^{-08}) are each correlated with sub-population, but PC3 has the strongest association that is also associated with the MR instrumental variable BMI-PGS making it a possible confounder in MR analysis, but only if sub-population was not already included in the MR models as a covariate.

Supplementary Figure 14: Forest plot of observational and MR sensitivity analyses. A forest plot of effect estimates (points) and 95% confidence intervals (whiskers) for MR (tsls, blue) and observational (obs, green) effect estimates for the weighted NEO (wNEO), weighted NEO sensitivity analyses (wNEO_sa), and the Leiderdorp (sub-)populations.

Supplementary Figure 15: Correlation between wNEO and wNEO sensitivity analysis MR estimates. Scatter plot of MR effect estimates in the weighted NEO (x-axis) and weighted NEO sensitivity analysis (y-axis). Point estimates for all metabolites in each of the three dietary states response (red), postprandial (blue), and fasting (green) are provided.

Supplementary Figure 16: NEO and Wurtz et al MR estimate scatter plot. Each of the 57 fasting, matched, metabolites are represented by a point. The x-axis provides the MR effect estimate as observed by Wurtz et al, and the y-axis is the MR effect estimate observed in this study (wNEO framework). The dotted black line is an equivalency line. The blue line is the best fit regression line. Estimates that were defined as associated in both NEO and Wurtz et al are colored green, those specific to NEO are colored red, those specific to Wurtz et al are colored blue, and those with no MR association in either study are purple. The associated metabolites are labelled with their metabolite ID in the plot. See **Supplementary Table 1** for further annotation.

Supplementary Figure 17: Postprandial observational profile. *Upper*: A tile plot of observational effect estimates for lipoproteins and lipoprotein ratios in the postprandial state. Tiles with an effect estimate provided in text are those with a p-value smaller than 0.05. The lipoproteins are labeled and color corradiated the x-axis, and the component or ratio being measured is along the y-axis. *Lower*: A dot plot or profile of postprandial observational effect estimates for lipoproteins (x-axis) ordered by lipoprotein size or density is provided to illustrate the correlation between effect estimates (y-axis) within a lipoprotein and the structure of estimates between lipoproteins by size. The component or measurement of each lipoprotein are defined by the color as described in the key. All effect estimates can be found in **Supplementary Table 4**.

Supplementary Figure 18: Fasting and Postprandial MR profiles. (A) Fasting MR and **(B)** Postprandial MR. *Upper*: A tile plot of MR effect estimates for lipoproteins and lipoprotein ratios in the fasting state. Tiles with an effect estimate provided in text are those with a p-value smaller than 0.05. The lipoproteins are labeled and color corradiated the x-axis, and the

component or ratio being measured is along the y-axis. *Lower*: A dot plot or profile of fasting MR effect estimates for lipoproteins (x-axis) ordered by lipoprotein size or density is provided to illustrate the correlation between effect estimates (y-axis) within a lipoprotein and the structure of estimates between lipoproteins by size. The component or measurement of each lipoprotein are defined by the color as described in the key. All effect estimates can be found in **Supplementary Table 4**.

The association between body mass index and metabolite response to a liquid mixed meal challenge: a Mendelian randomization study; Hughes et al

Supplementary Figure 1

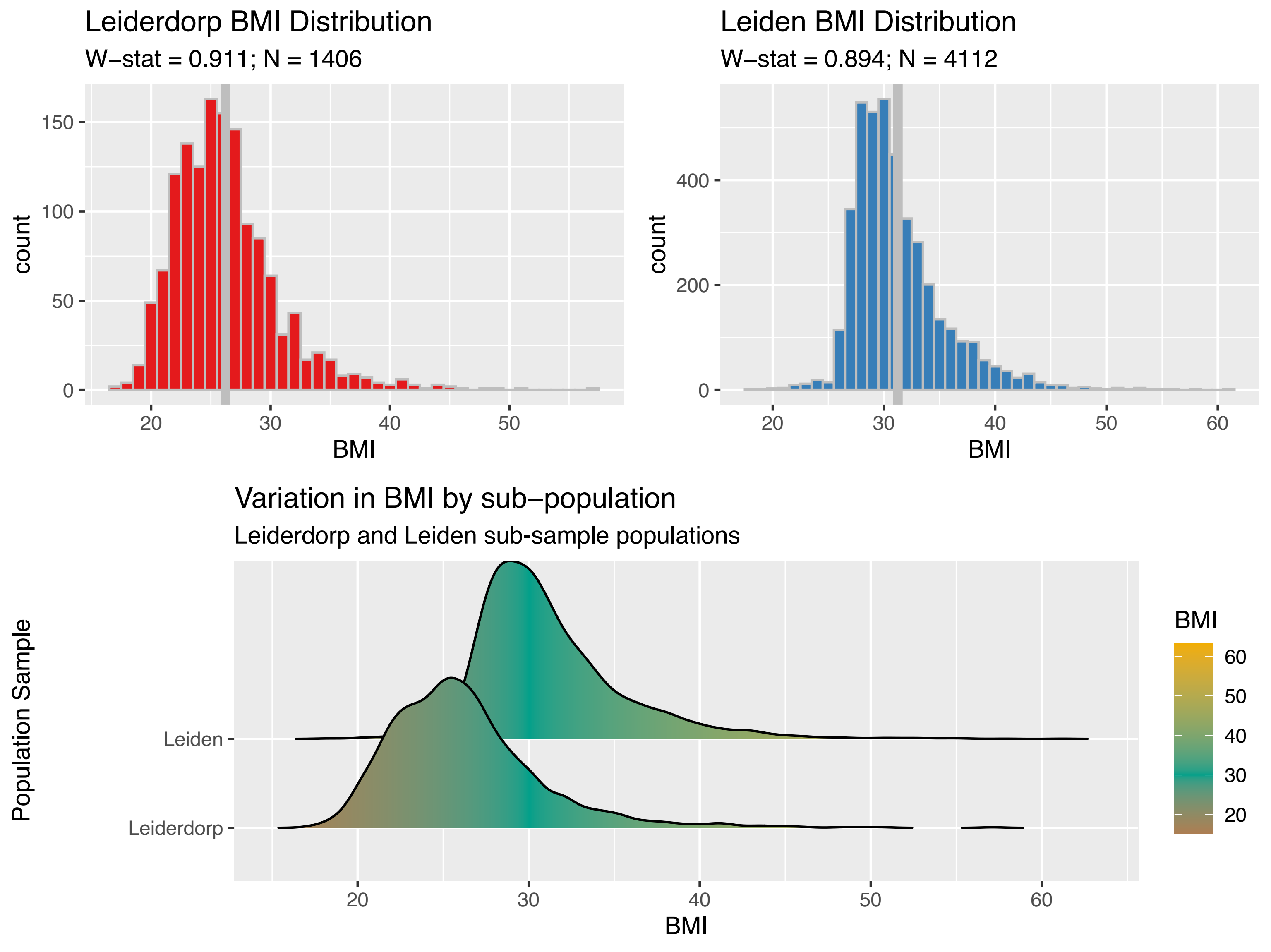

The association between body mass index and metabolite response to a liquid mixed meal challenge: a Mendelian randomization study; Hughes et al

Supplementary Figure 2

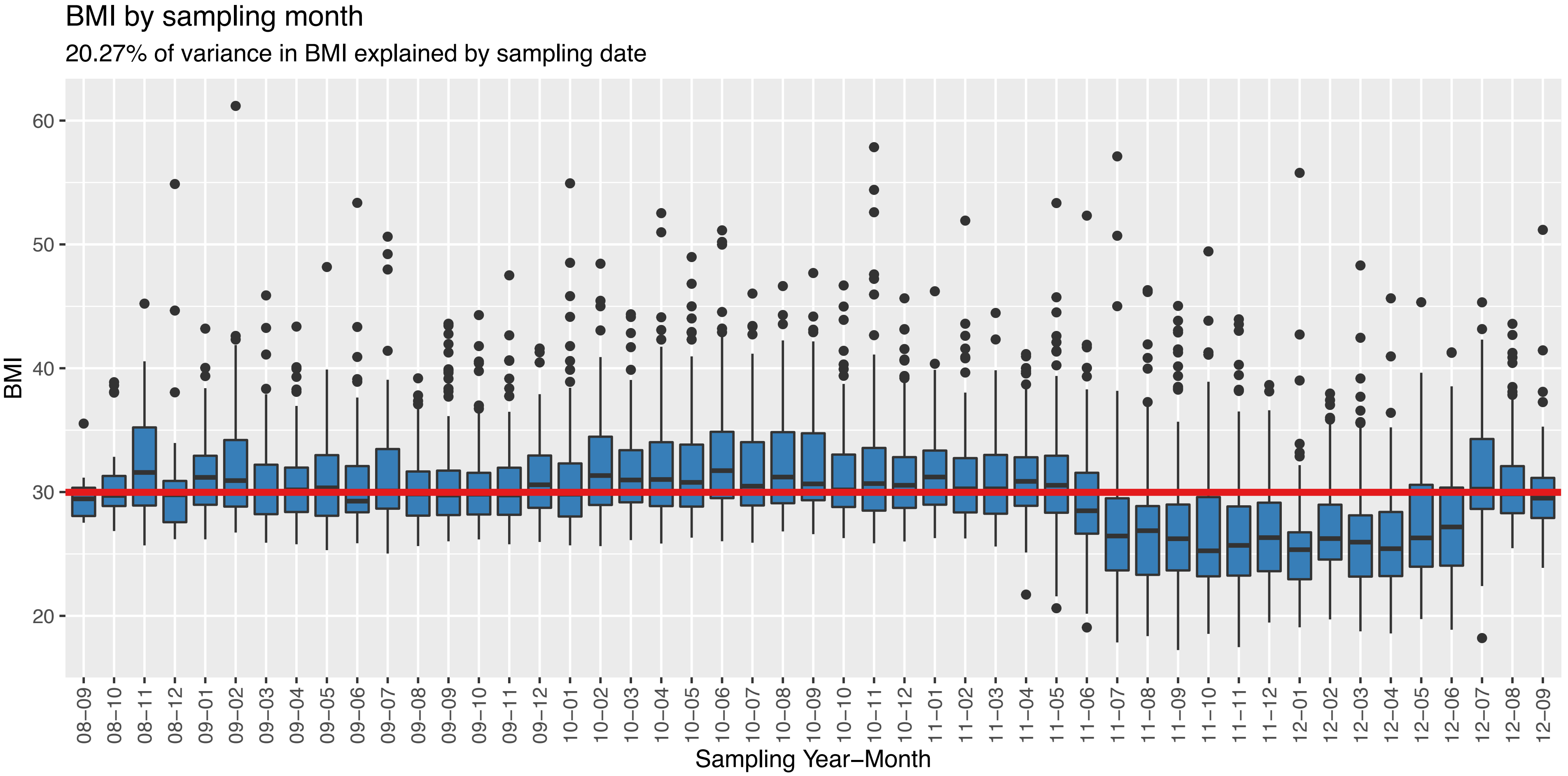

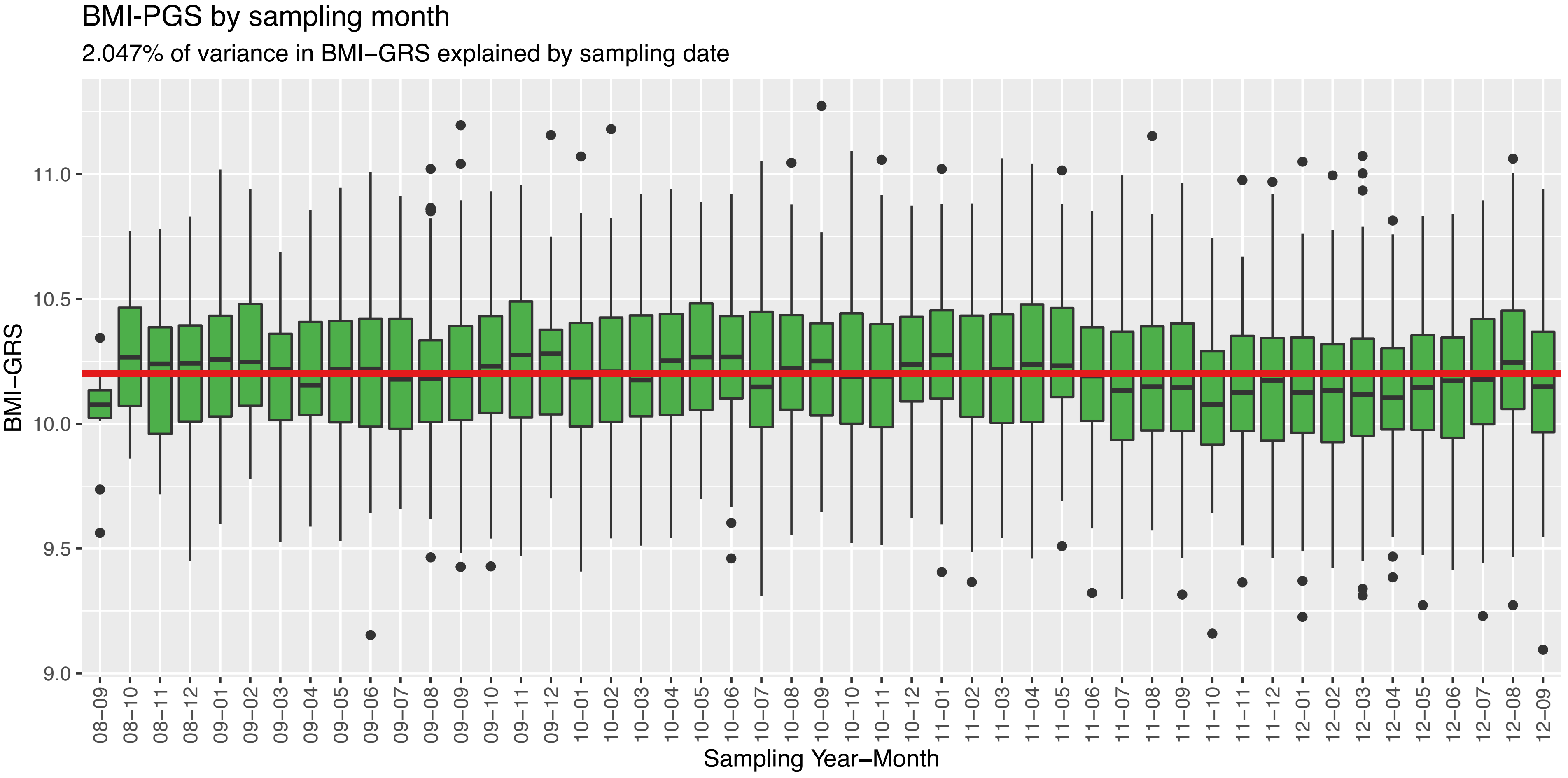

The association between body mass index and metabolite response to a liquid mixed meal challenge: a Mendelian randomization study; Hughes et al

Supplementary Figure 3

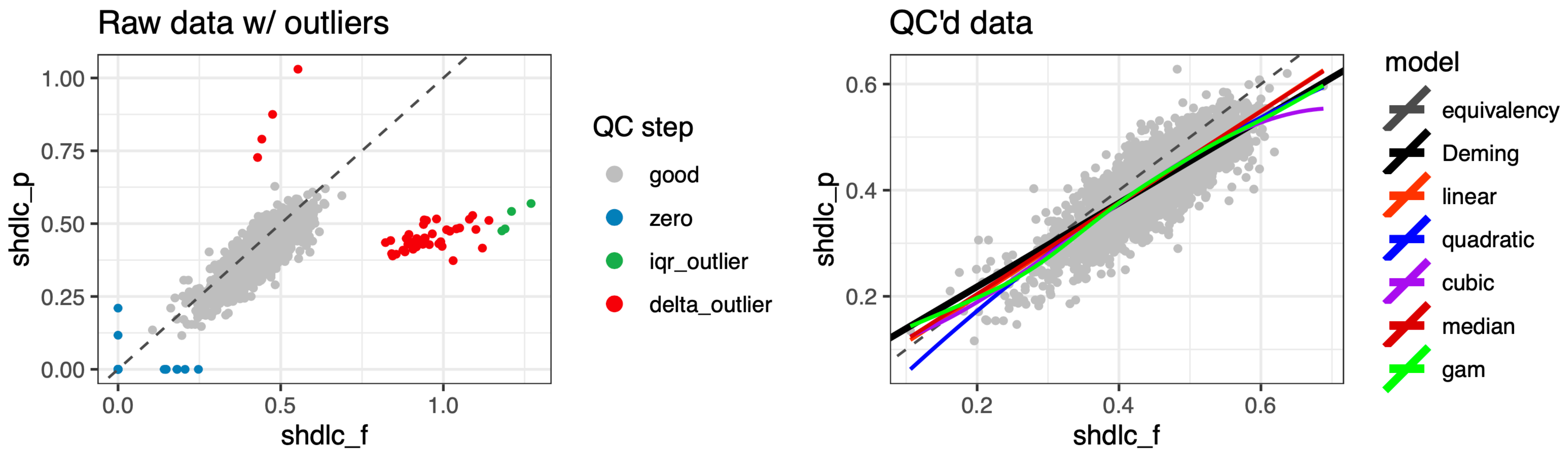

Supplementary Figure 4

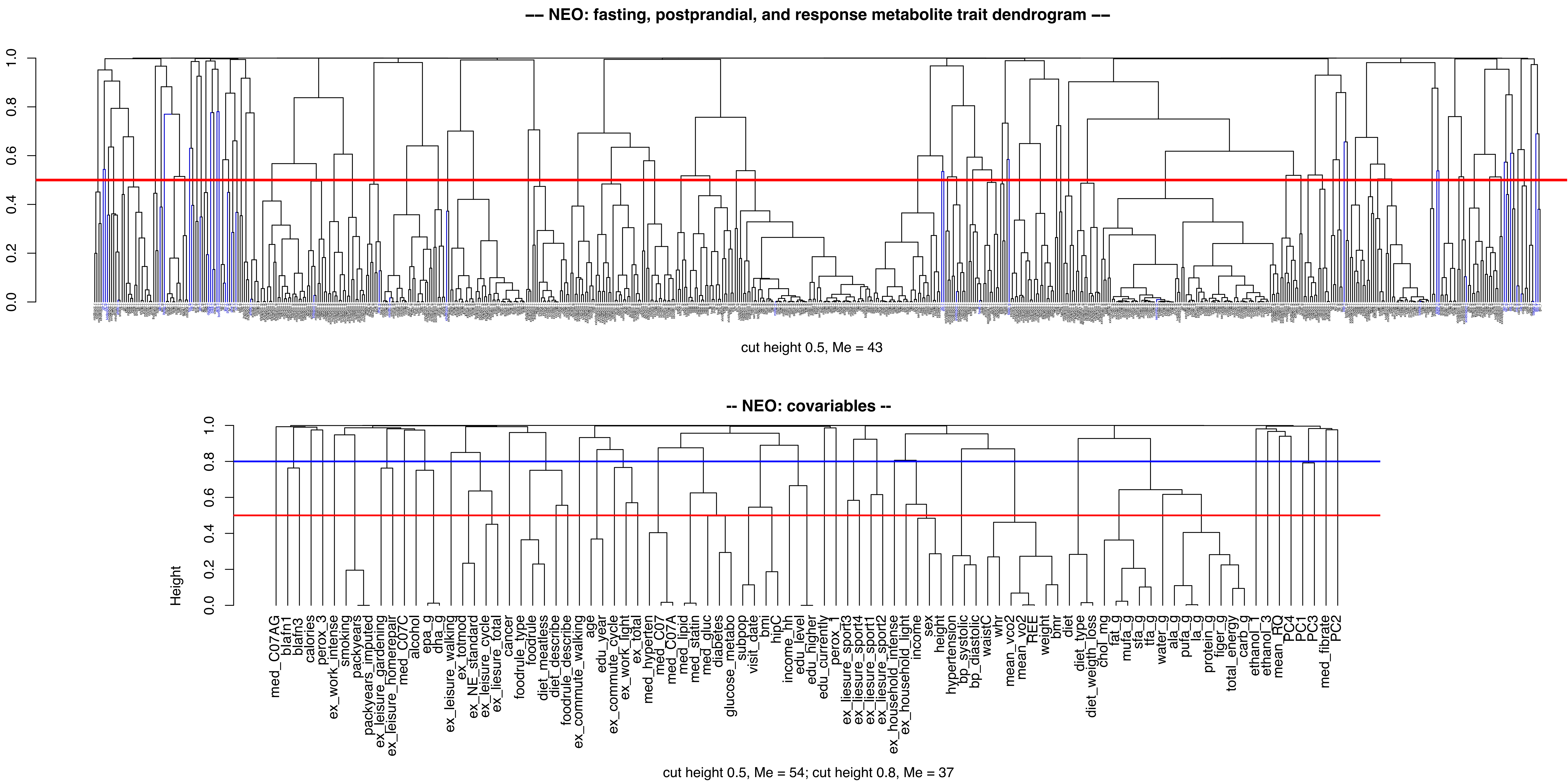

The association between body mass index and metabolite response to a liquid mixed meal challenge: a Mendelian randomization study; Hughes et al

Supplementary Figure 5

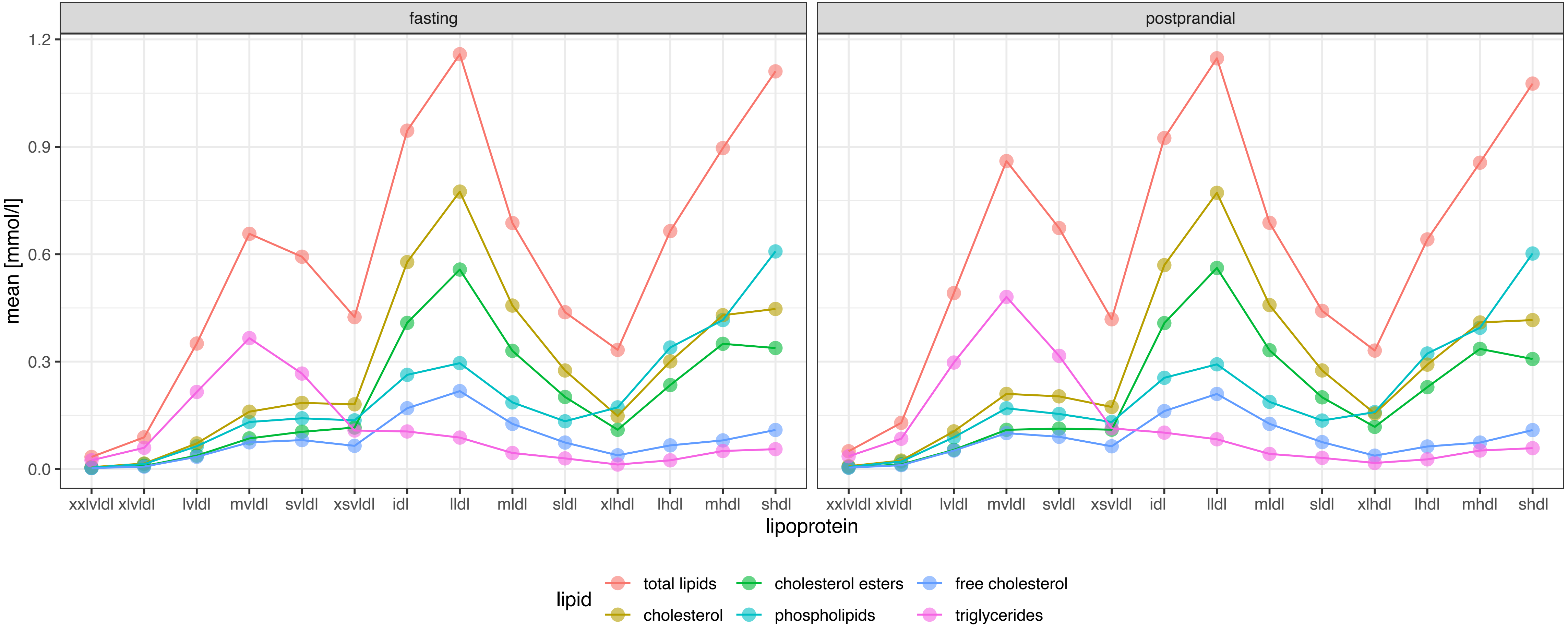

The association between body mass index and metabolite response to a liquid mixed meal challenge: a Mendelian randomization study; Hughes et al

Supplementary Figure 6

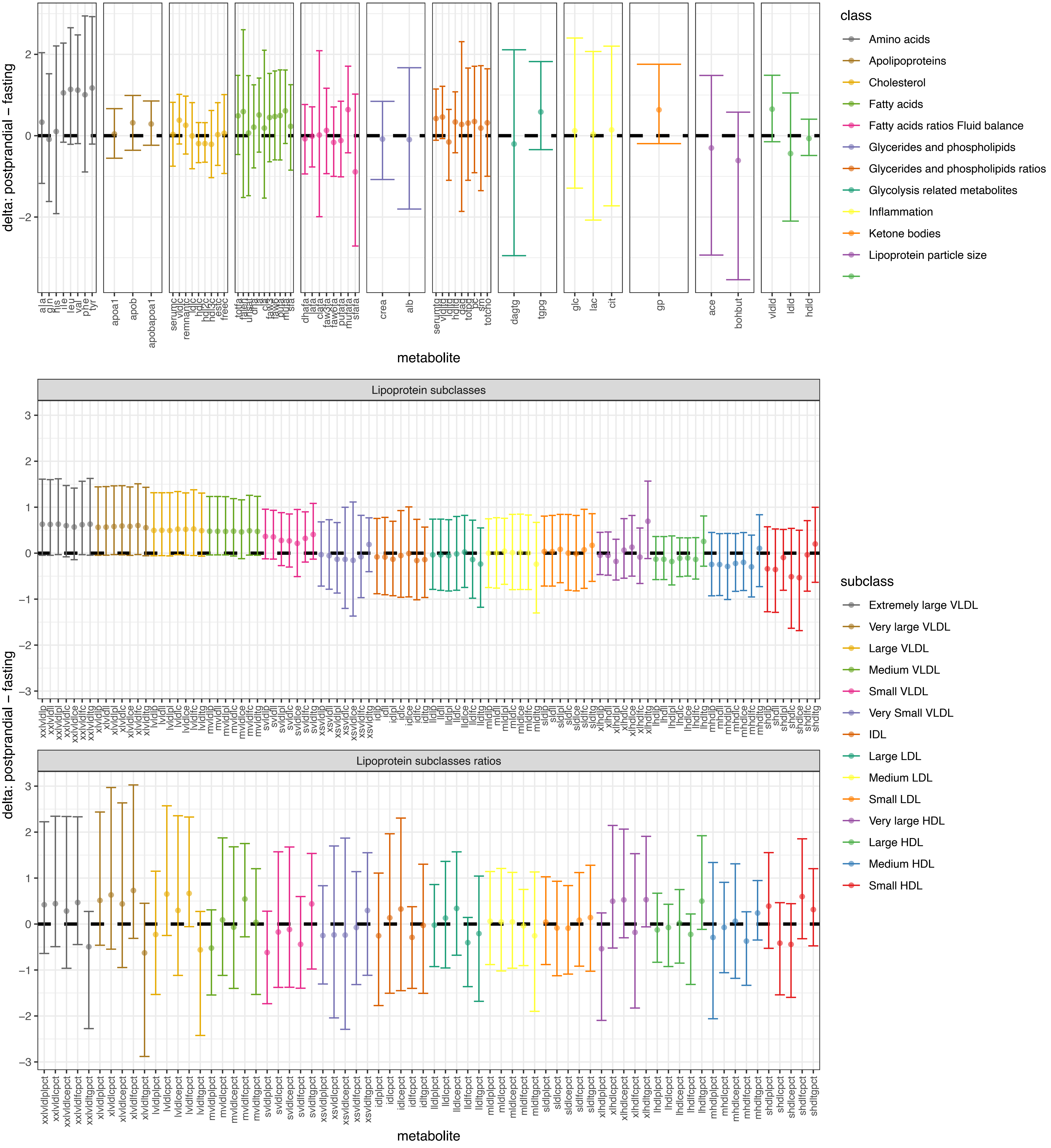

Supplementary Figure 7

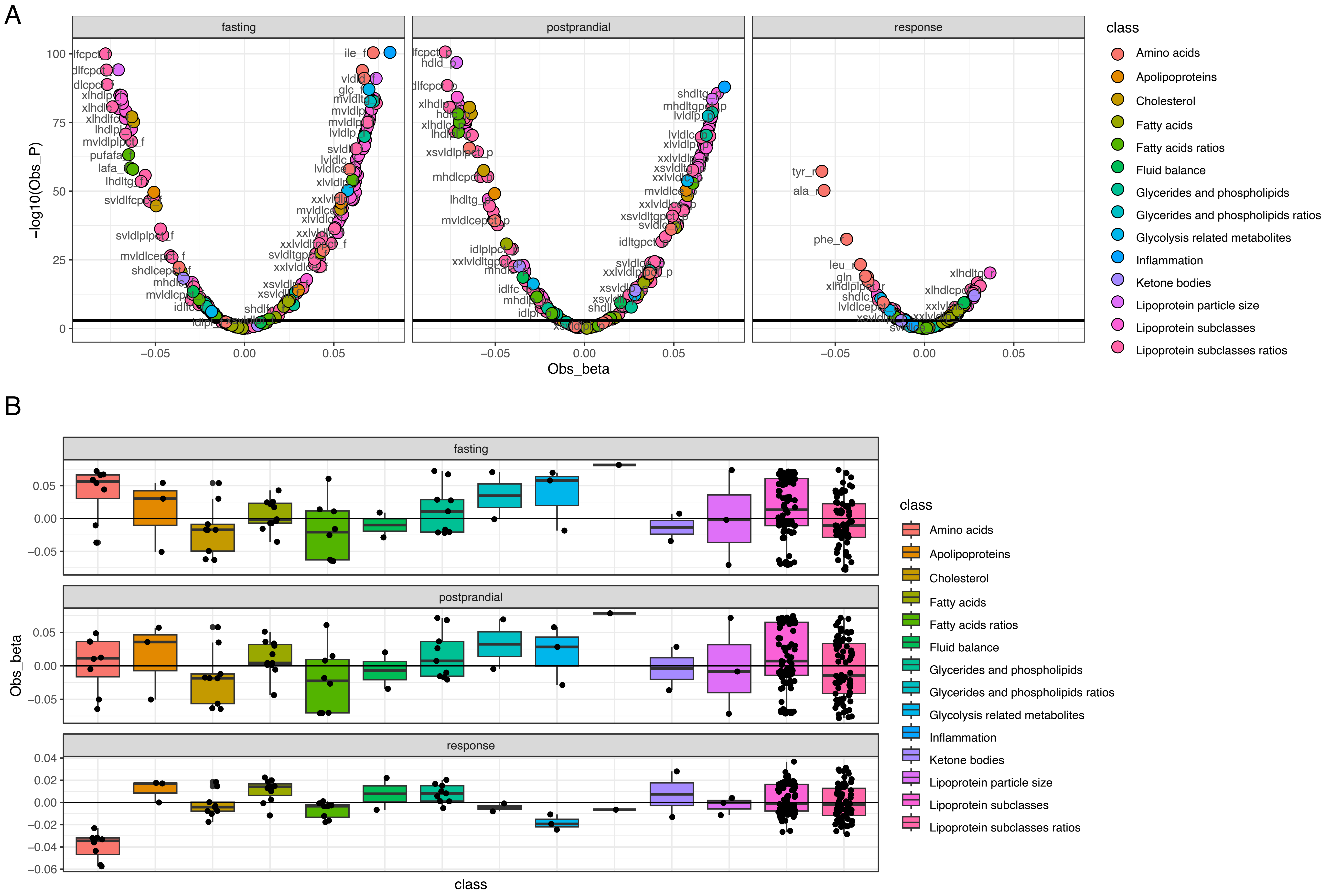

The association between body mass index and metabolite response to a liquid mixed meal challenge: a Mendelian randomization study; Hughes et al

Supplementary Figure 8

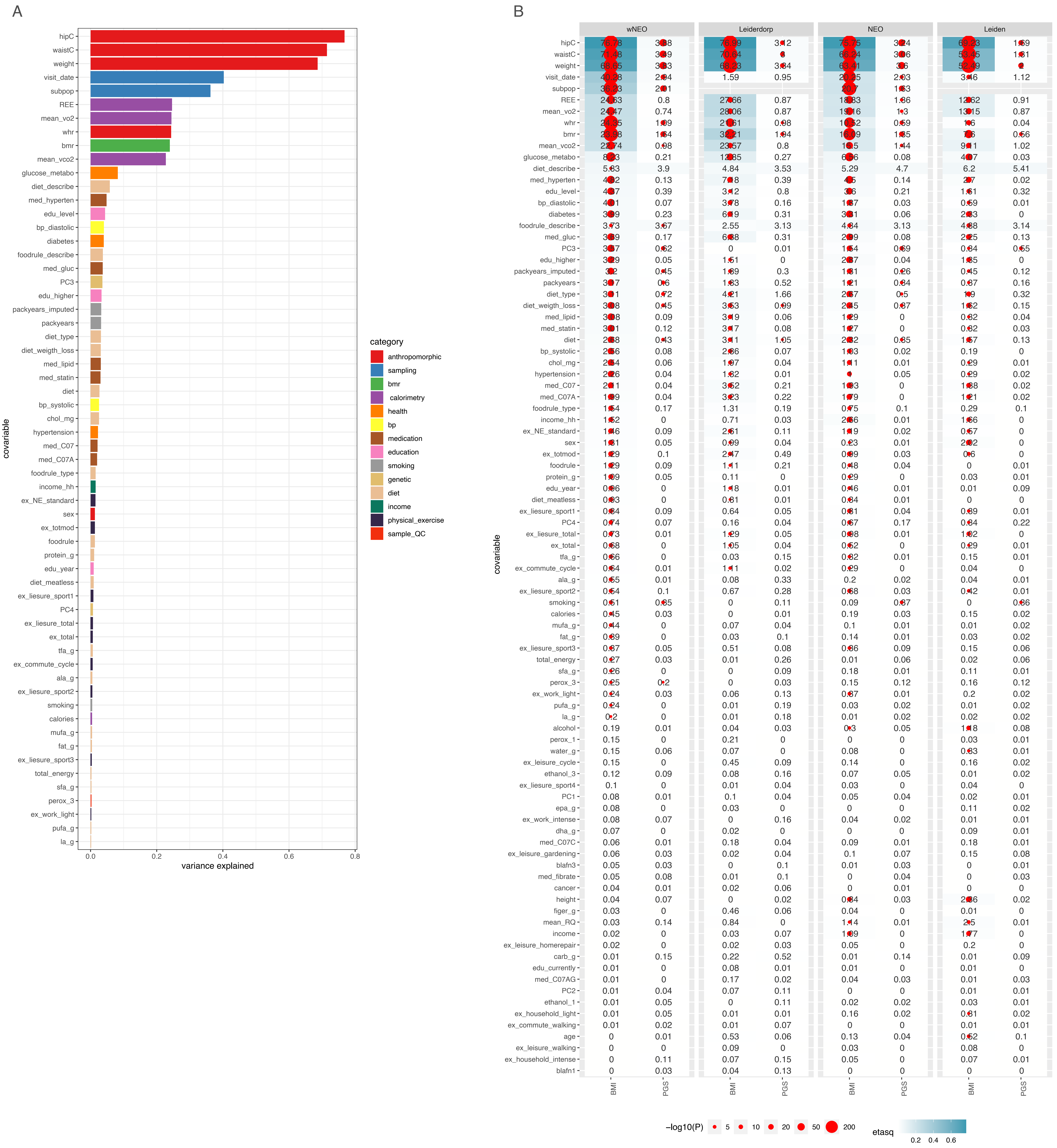

The association between body mass index and metabolite response to a liquid mixed meal challenge: a Mendelian randomization study; Hughes et al

Supplementary Figure 9

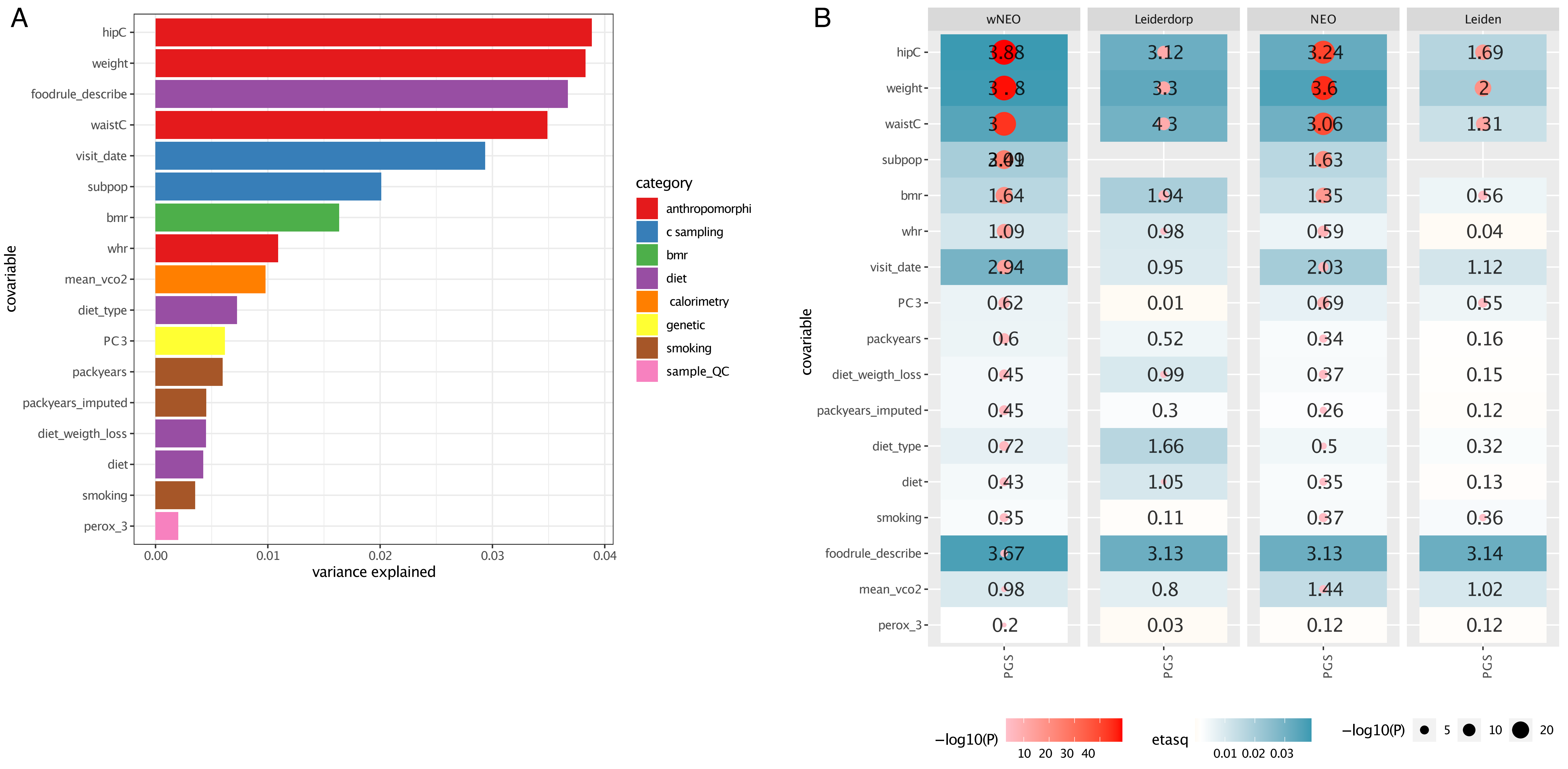

The association between body mass index and metabolite response to a liquid mixed meal challenge: a Mendelian randomization study; Hughes et al

Supplementary Figure 10

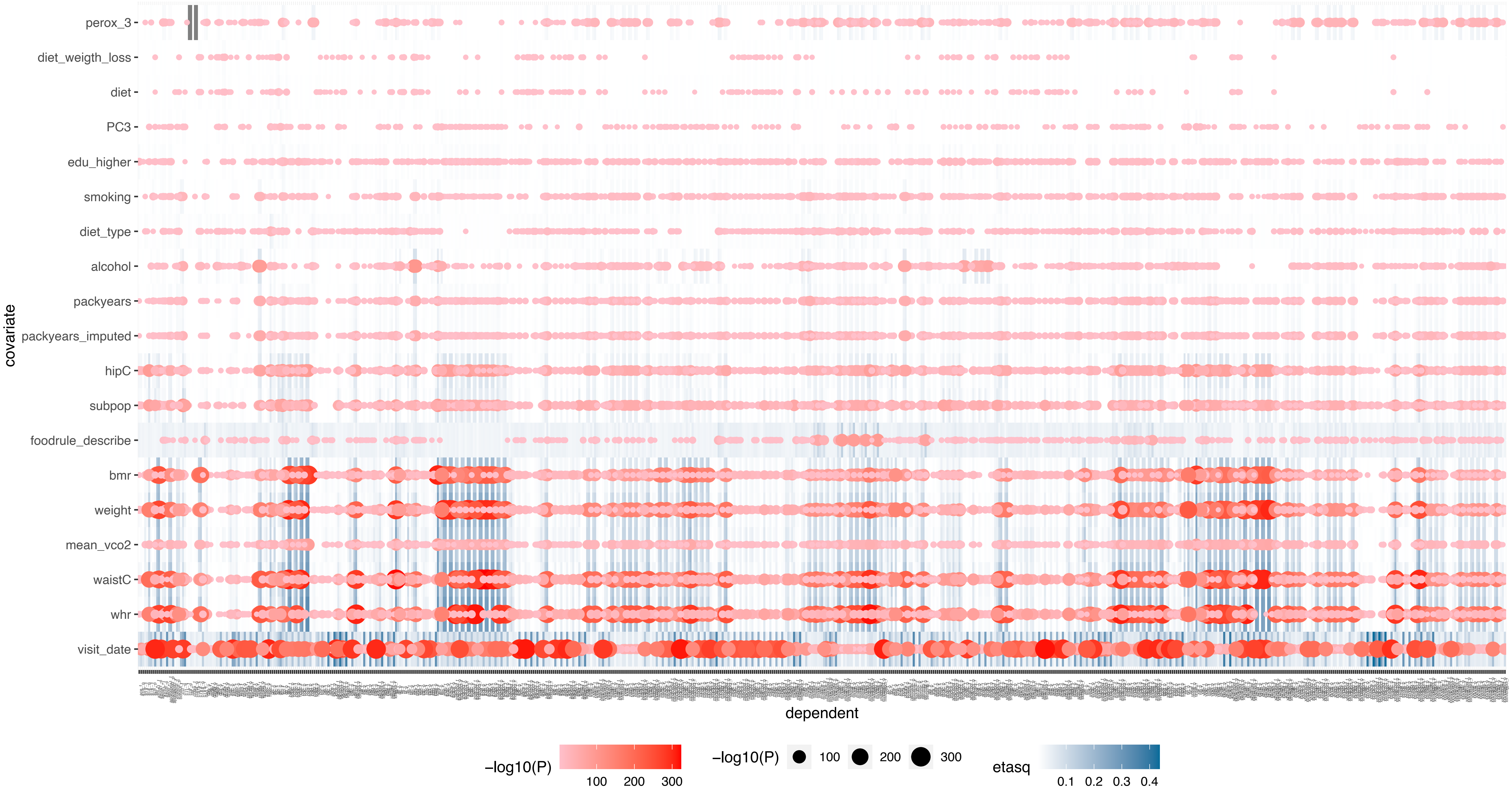

Supplementary Figure 11

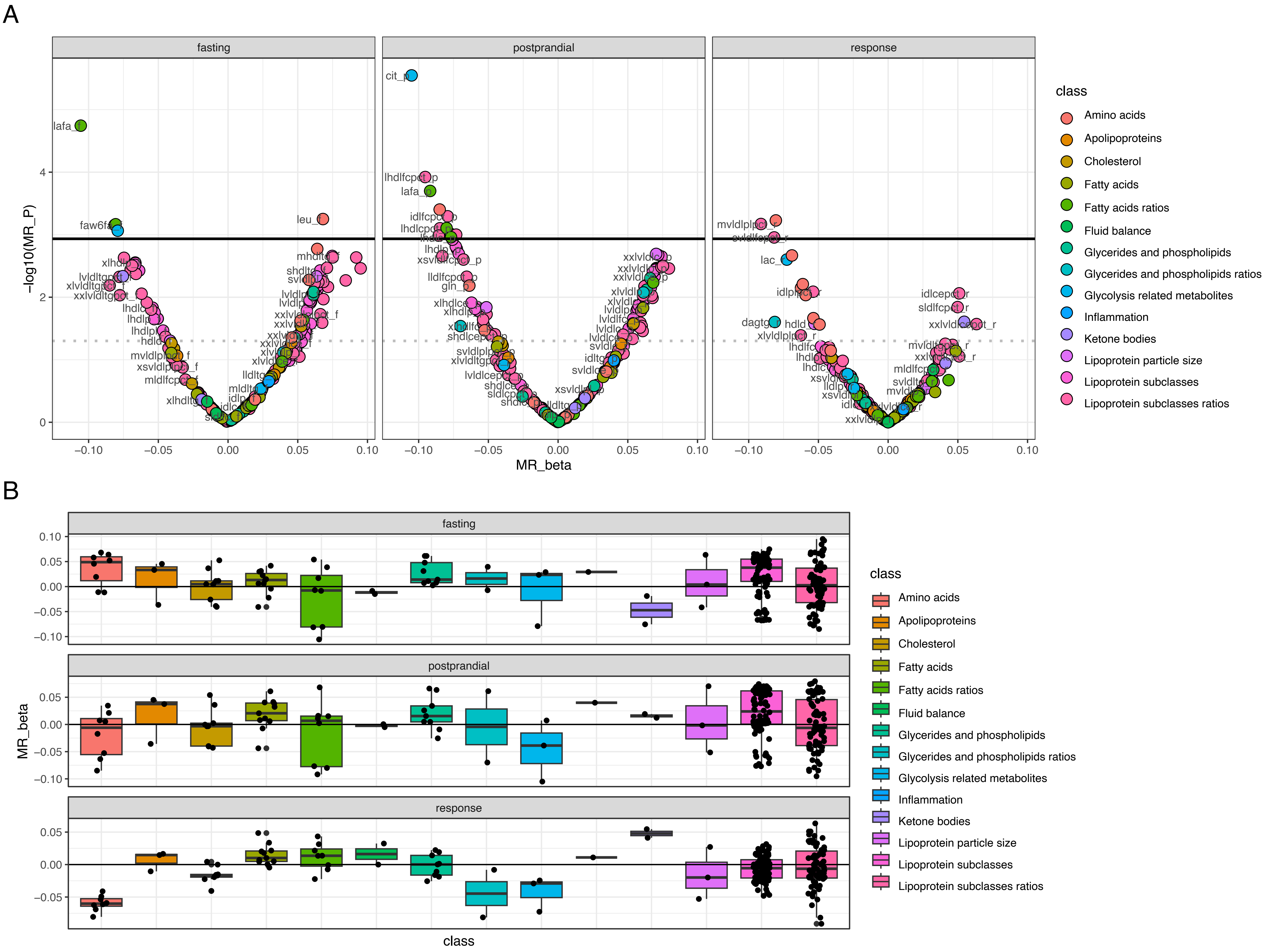

Supplementary Figure 12

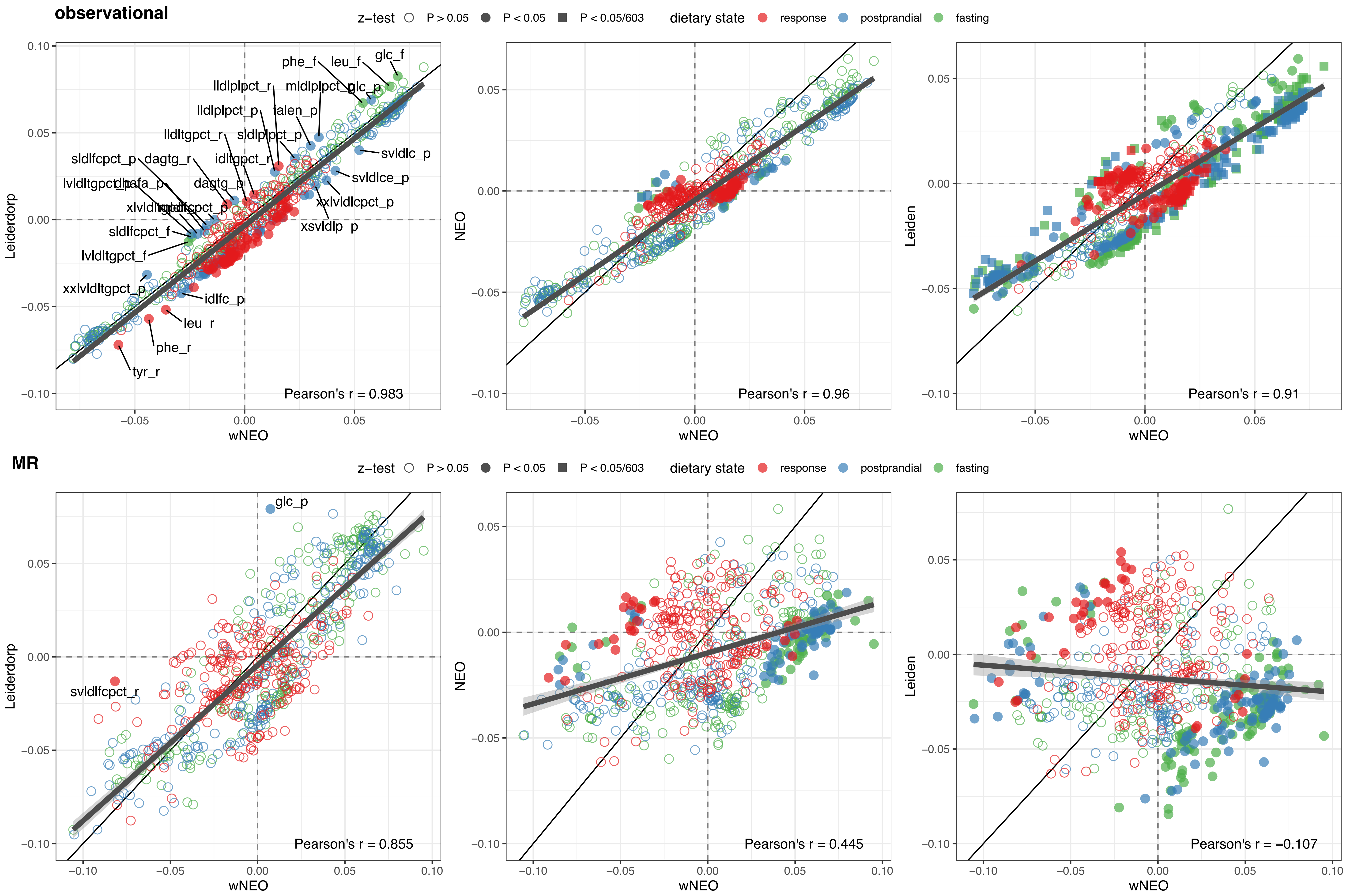

The association between body mass index and metabolite response to a liquid mixed meal challenge: a Mendelian randomization study; Hughes et al

Supplementary Figure 13

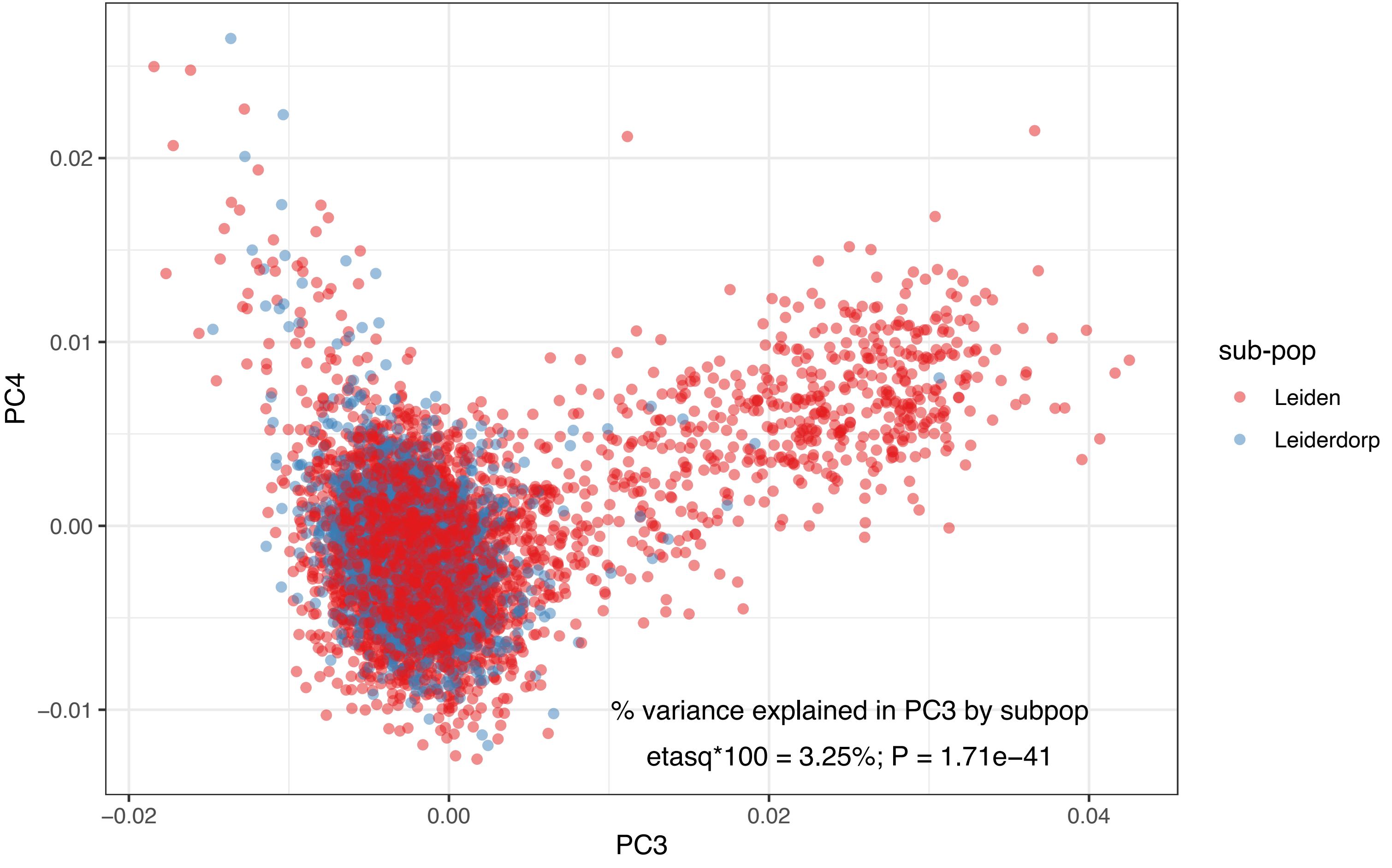

The association between body mass index and metabolite response to a liquid mixed meal challenge: a Mendelian randomization study; Hughes et al

Supplementary Figure 14

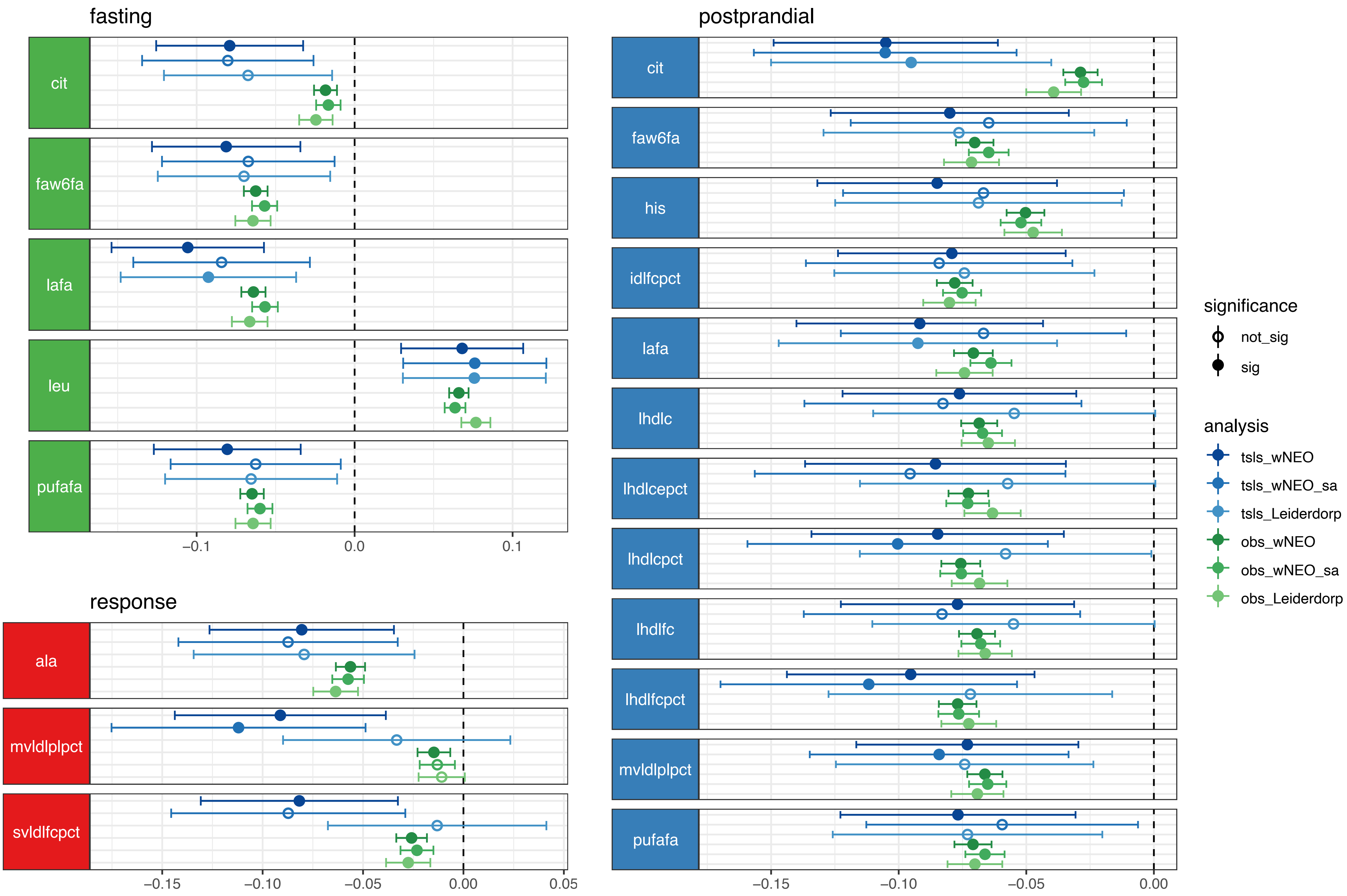

The association between body mass index and metabolite response to a liquid mixed meal challenge: a Mendelian randomization study; Hughes et al

Supplementary Figure 15

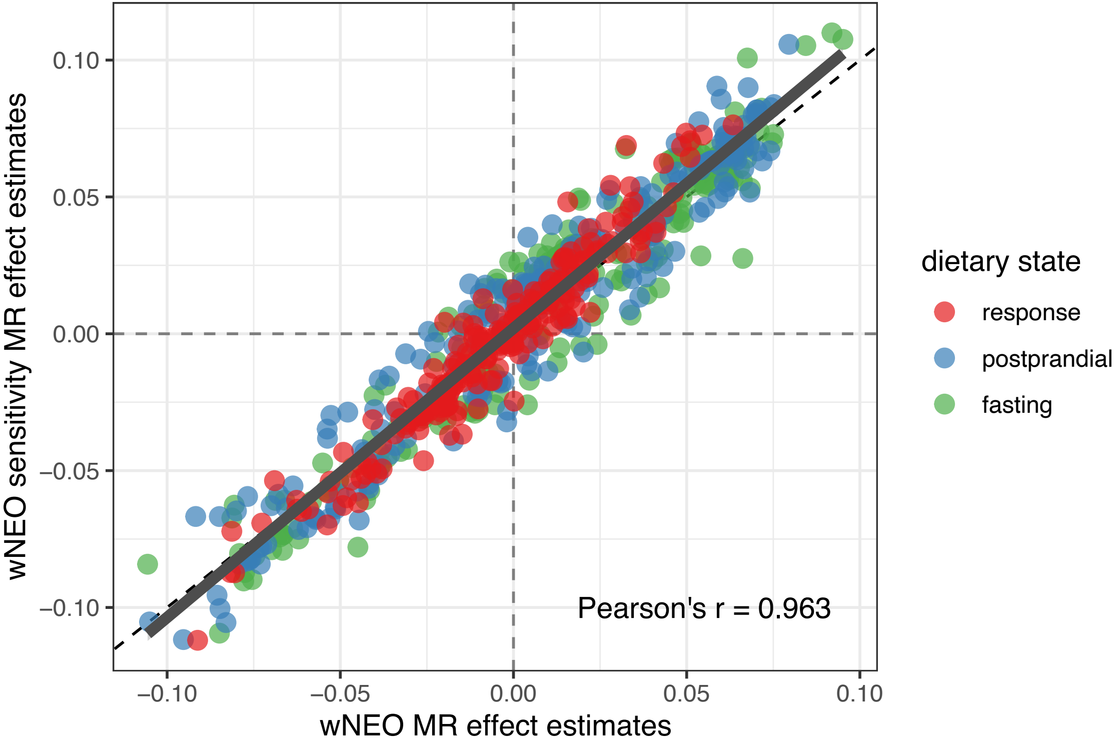

The association between body mass index and metabolite response to a liquid mixed meal challenge: a Mendelian randomization study; Hughes et al

Supplementary Figure 16

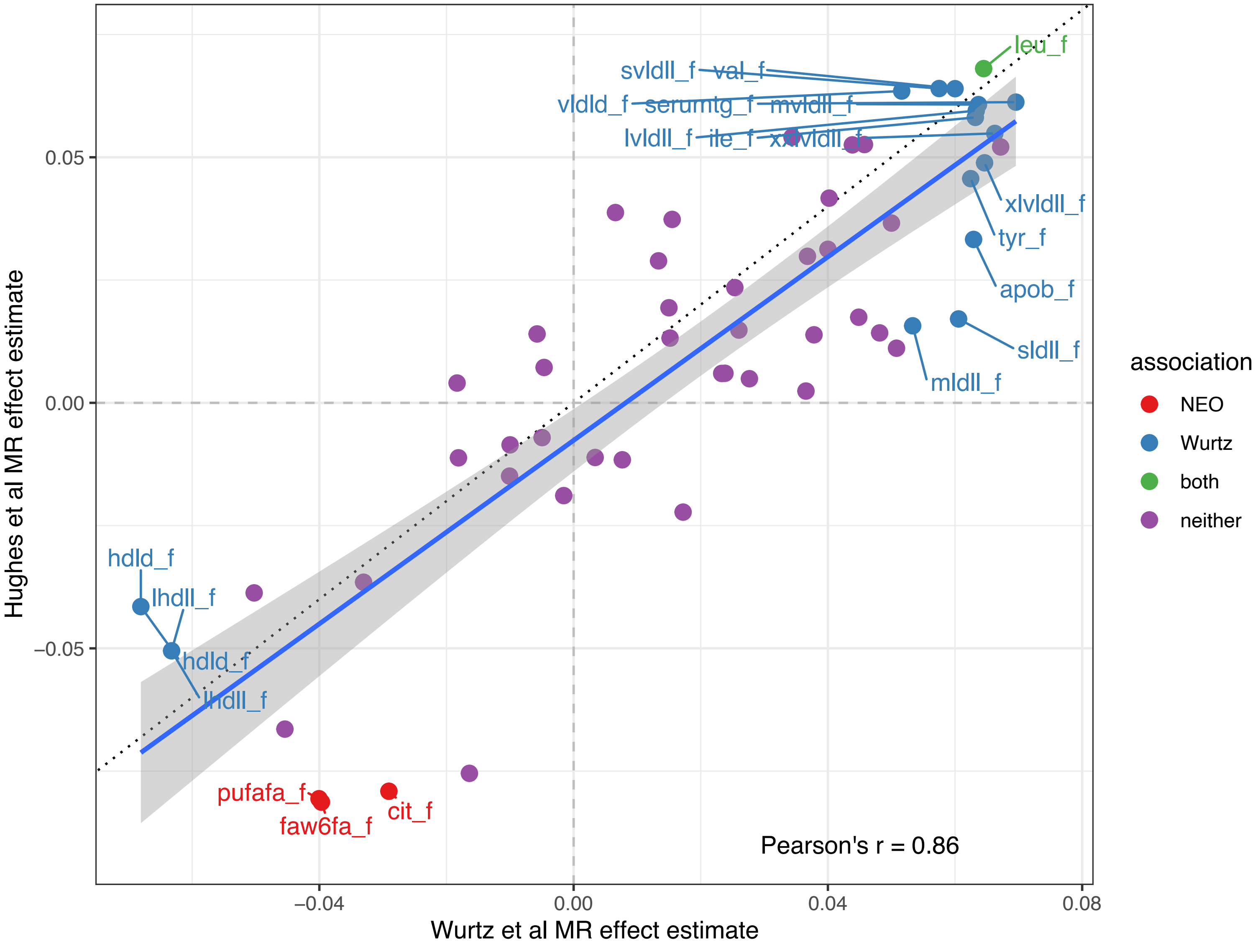

The association between body mass index and metabolite response to a liquid mixed meal challenge: a Mendelian randomization study; Hughes et al

Supplementary Figure 17

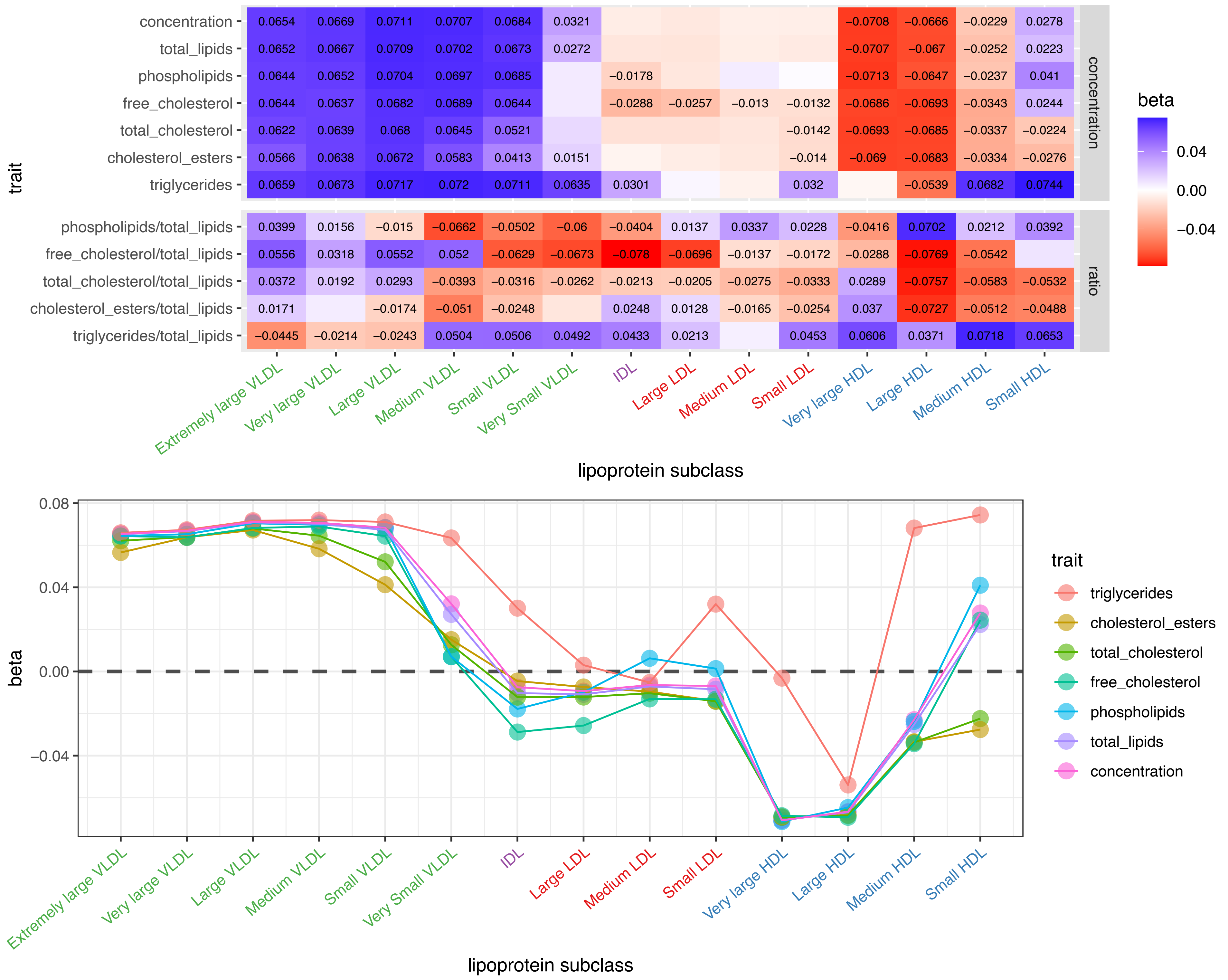

The association between body mass index and metabolite response to a liquid mixed meal challenge: a Mendelian randomization study; Hughes et al

Supplementary Figure 18

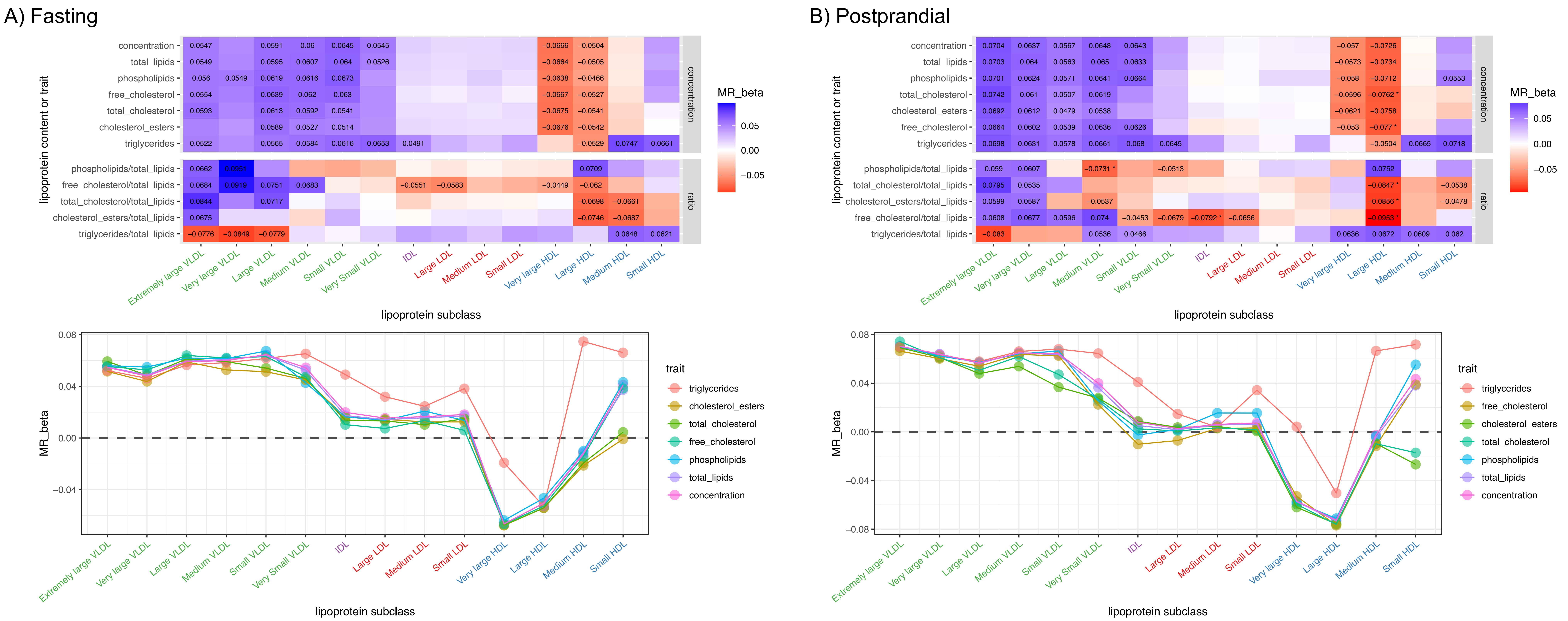

Metaboprep Log File:

I. Setting up your pipeline
-Your data directory is: /Volumes/MRC-IEU-research/projects/wt1/wp1/010/working/data/source/release2/metaboprep/
- Your metabolite data file is: source_metabolites_extracted.csv
Your metabolite data file was identified as a previously processed flat text file.
- Your provided feature annotation file to process is: source_metabolites_feature_data.txt
- Your provided sample | batch annotation file to process is: source_metabolite_batch_data.txt
- Your declared platform is: Other
- Feature filtering: Your declared feature missingness level is: 0.2
- Sample filtering: Your declared sample missingness level is: 0.2
- Sample filtering: Your declared total peak area filter level, in standard deviations from the mean is: 5
- Sample filtering: Your declared principal component (PC1 and PC2) exclusion, in standard deviations from the mean is: 5
- Metabolite independence: Your declared tree cut height is: 0.5
- Reading in your csv metabolite file
- Reading in your txt feature annotation file
- Reading in your txt sample annotation file
- Assuming sample IDs are in column 1 and redefining rownames
III. Your data has been read in.

-Your data has 5744 individuals and 606 metabolites.
-There are also 3 sample annotation|batch variables.
-There are also 10 feature annotation|batch variables.

IV. Estimating Summary Statistics On Raw Data Set.
a. Estimating summary statistics for samples
- Writing sample summary statistics to file.
b. Estimating summary statistics for features.
- Estimating the number of indpendent features.
- 12 features excluded from analysis for (>20% missingness) n smaller than 4595.
- Generating Correlation Matrix.
- Generating Distance Matrix.
- Constructing dendrogram.
- Performing tree cut. Cut height defined at 0.5
- Identifying independent features.
- Writing feature summary statistics to file.
c. Performing principle component analysis and identifying outliers.
- Re-Writing sample summary statistics to file to include PCs.
- Writing PC statistics to file.

V. Performing data filtering.
a. Performing data filtering.

- QCstep: estimate initial sample missingness.
- QCstep: exclude those sample with missingness >= 80%.
    * 9 sample(s) excluded for extreme missingness.
- QCstep: estimate initial feature missingness.
- QCstep: exclude those features with missingness >= 80%.
    * 0 features excluded for extreme missingness.
- QCstep: RE-estimate sample missingness.
- QCstep: exclude those sample with missingness >= 20%.
    * 217 sample(s) excluded for user defined missingness.
- QCstep: RE-estimate feature missingness.
- QCstep: exclude those features with missingness >= 20%.
    * 3 feature(s) excluded for user defined missingness.
- QCstep: estimate total peak area.
- QCstep: exclude those features with TPA >= 5SD from the mean.
    * 0 samples excluded for user defined TPA SD from the mean.
- QCstep: re-identify independent features through correlation analysis and dendrogram clustering.
    - using currently QCd data.
- Estimating the number of indpendent features.
    - Generating Correlation Matrix.
    - Generating Distance Matrix.
    - Constructing dendrogram.
    - Performing tree cut. Cut height defined at 0.5
    - Identifying independent features.
    * 78 independent features identified.
- QCstep: Perform Principle Componenet Analysis of currently QC'd data.
- QCstep: Identify PC 1-2 outliers >= +/-5SD of the mean.
    * 0 samples excluded as PC outliers.

b. Writing QC data to file.

VI. Estimating Summary Statistics on Filtered Data Set.

a. Estimating summary statistics for Filtered samples

b. Writing filtered sample summary statistics to file.

c. Estimating summary statistics for filtered features.

- Estimating the number of indpendent features.
    - Generating Correlation Matrix.
    - Generating Distance Matrix.
    - Constructing dendrogram.
    - Performing tree cut. Cut height defined at 0.5
    - Identifying independent features.
    - A total of 78 independent features were identified in the total filtered data set.

d. Writing feature summary statistics to file.

e. Performing principle component analysis on final filtered data set.

The number of informative principle components:

1. Cattel's Scree Test : acceleration factor = 2
1. Parrallel Analysis = 18

f. Re-Writing filtered sample summary statistics to file to include PCs.

g. Writing PC statistics to file.

VII. Generate Data Description pdf report.

metaboprep metabolomics data preparation report

2021-08-19

metaboprep report relates to:

- Project: NEO
- Platform: Other

The `metaboprep` `R` package performs three operations:

1. Provides an assessment and summary statistics of the raw metabolomics data.
2. Performs data filtering on the metabolomics data.
3. Provides an assessment and summary statistics of the filtered metabolomics data, particularly in the context of batch variables when available.

This report provides descriptive information for raw and filtered metabolomics data for the project NEO.

The data filtering workflow is as follows:

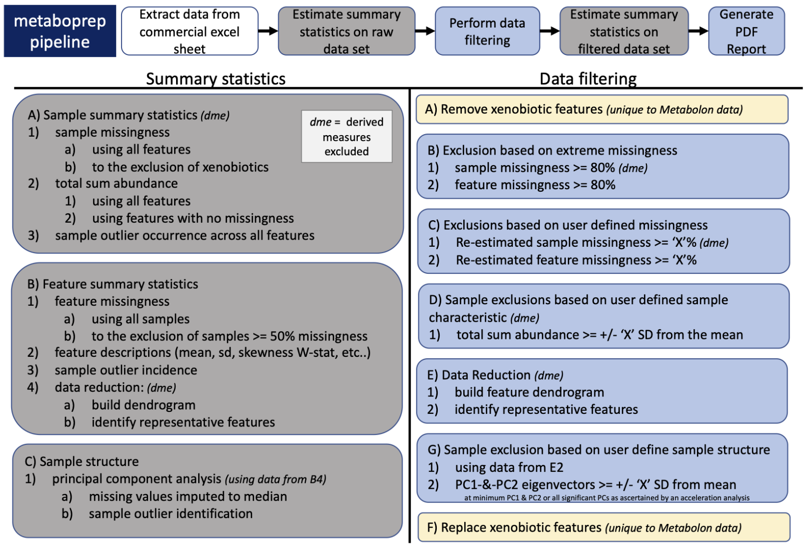

1. Issues can be raised on GitHub.
2. Questions relating to the `metaboprep` pipeline can be directed to David Hughes: d.a.hughes@bristol.ac.uk.
3. `metaboprep` is published in Journal to be determined and can be cited as:

1 Sample size of NEO data set

data.set	raw.data	filtered.data
number of samples	5744	5518
number of features	606	603

1.1 Missingness

Missingness is evaluated across samples and features using the original/raw data set.

1.1.1 Visual structure of missingness in your raw data set.

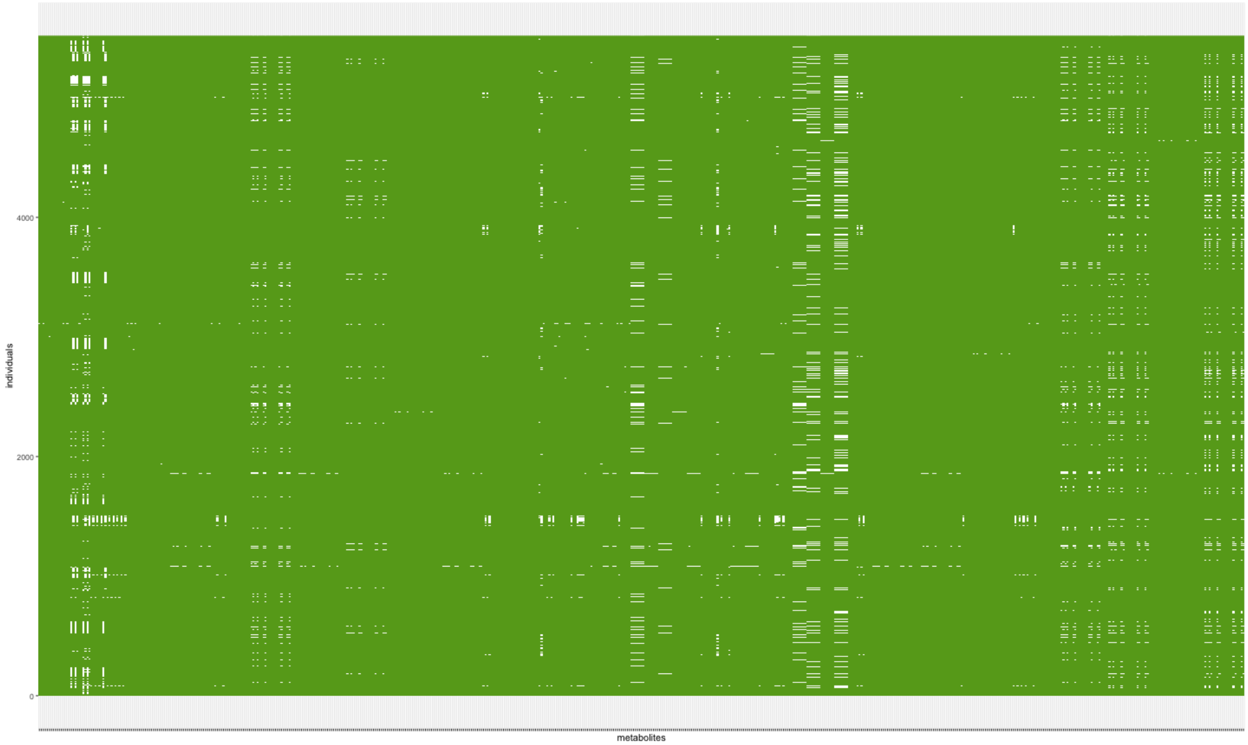

Figure Legend: Missingness structure across the raw data table. White cells depict missing data. Individuals are in rows, metabolites are in columns.

1.1.2 Summary of sample and feature missingness

–– Initial raw data set: Estimates of missingness for samples and features ––

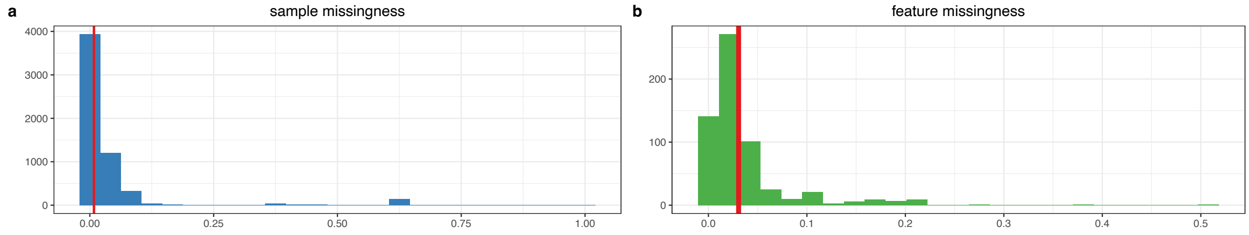

c

percentile	samples	features
0	0	0.0084
0.25	0	0.0108
0.5	0.0083	0.0306
0.75	0.033	0.0336
1	1	0.5164

d

percent.missing	sample.size
10% feature missingness	5169.6
20% feature missingness	4595.2
30% feature missingness	4020.8
40% feature missingness	3446.4
50% feature missingness	2872.0

Figure Legend: Raw data - (a) Distribution of sample missingness with sample mean illustrated by the red vertical line. (b) Distribution of feature missingness sample mean illustrated by the red vertical line. (c) Table of sample and feature missingness percentiles. A tabled version of plot a and b. (d) Estimates of study samples sizes under various levels of feature missingness.

1.2 Data Filtering

1.2.1 Exclusion summary

exclusions	count
Extreme_sample_missingness	9
Extreme_feature_missingness	0
User_defined_sample_missingness	217
User_defined_feature_missingness	3
User_defined_sample_totalpeakarea	0
User_defined_sample_PCA_outliers	0

Table Legend: Six primary data filtering exclusion steps were made during the preparation of the data. (1) Samples with missingness >=80% were excluded. (2) features with missingness >=80% were excluded (xenobiotics are not included in this step). (3) sample exclusions based on the user defined threshold were excluded. (4) feature exclusions based on user defined threshold were excluded (xenobiotics are not included in this step). (5) samples with a total-peak-area or total-sum-abundance that is >= N standard deviations from the mean, where N was defined by the user, were excluded. (6) samples that are >= N standard deviations from the mean on principal component axis 1 and 2, where N was defined by the user, were excluded.

1.2.2 Metabolite or feature reduction and principal components

A data reduction was carried out to identify a list of representative features for generating a sample principal component analysis. This step reduces the level of inter-correlation in the data to ensure that the principal components are not driven by groups of correlated features.

data.reduction	count
total metabolite count	603
metabolites included in data reduction	603
number of metabolite clusters	78
number of representative metabolites	78

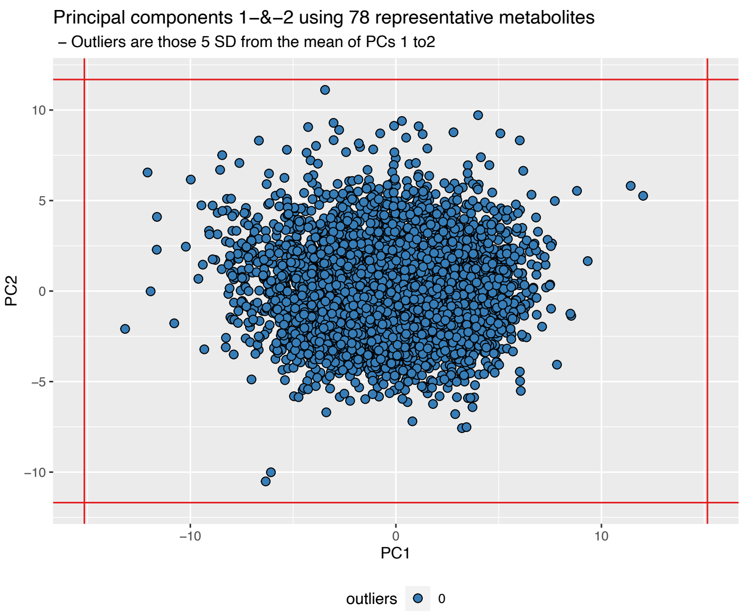

Figure Legend: The data reduction table on the left presents the number of metabolites at each phase of the data reduction (Spearman's correlation distance tree cutting) analysis. On the right principal components 1 and 2 are plotted for all individuals, using the representitve features identified in the data reduction analysis. The red vertical and horizontal lines indicate the standard deviation (SD) cutoffs for identifying individual outliers, which are plotted in red. The standard deviations cuttoff were defined by the user.

---

2 Filtered data

2.1 N

- The number of samples in data = 5518
- The number of features in data = 603

2.2 Relative to the raw data

- 226 samples were filtered out, given the user's criteria.
- 3 features were filtered out, given the user's criteria.
- Please review details above and your log file for the number of features and samples excluded and why.

2.3 Summary of filtered data

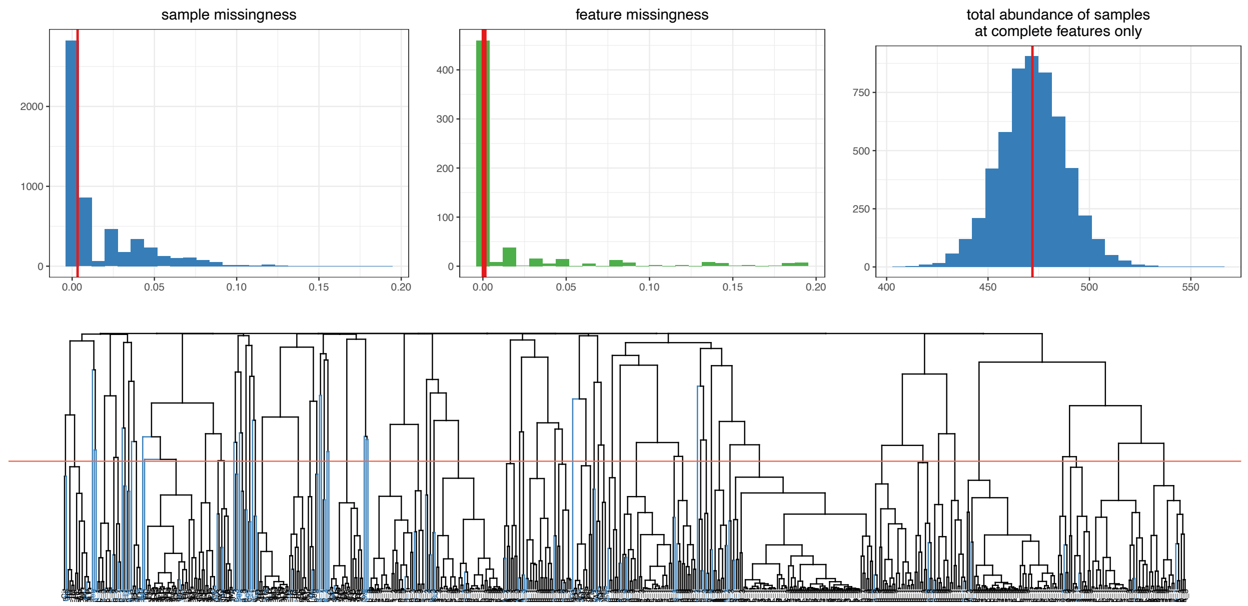

data.reduction	count
total metabolite count	603
metabolites included in data reduction	603
number of metabolite clusters	78
number of representative metabolites	78

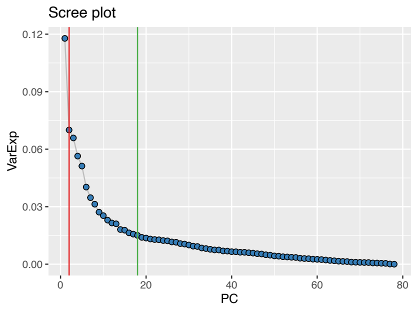

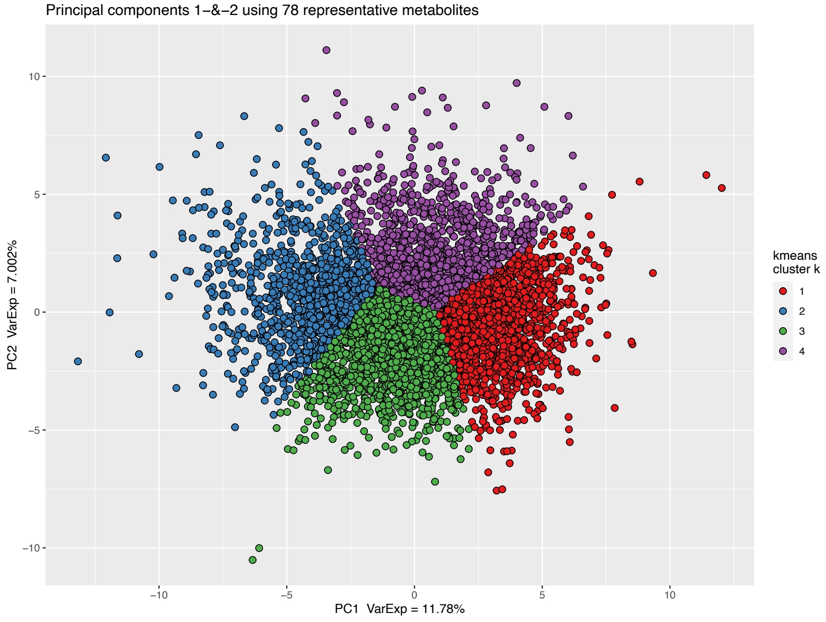

Figure Legend: Filtered data summary. Distributions for sample missingness, feature missingness, and total abundance of samples. Row two of the figure provides a Spearman's correlation distance clustering dendrogram highlighting the metabolites used as representative features in blue, the clustering tree cut height is denoted by the horizontal line. Row three provides a summary of the metabolite data reduction in the table, a Scree plot of the variance explained by each PC and a plot of principal component 1 and 2, as

derived from the representative metabolites. The Scree plot also identifies the number of PCs estimated to be informative (vertical lines) by the Cattel's Scree Test acceleration factor (red, n = 2) and Parallel Analysis (green, n = 18). Individuals in the PC plot were clustered into 4 kmeans (k) clusters, using data from PC1 and PC2. The kmeans clustering and color coding is strictly there to help provide some visualization of the major axes of variation in the sample population(s).

2.4 Structure among samples: top 5 PCs

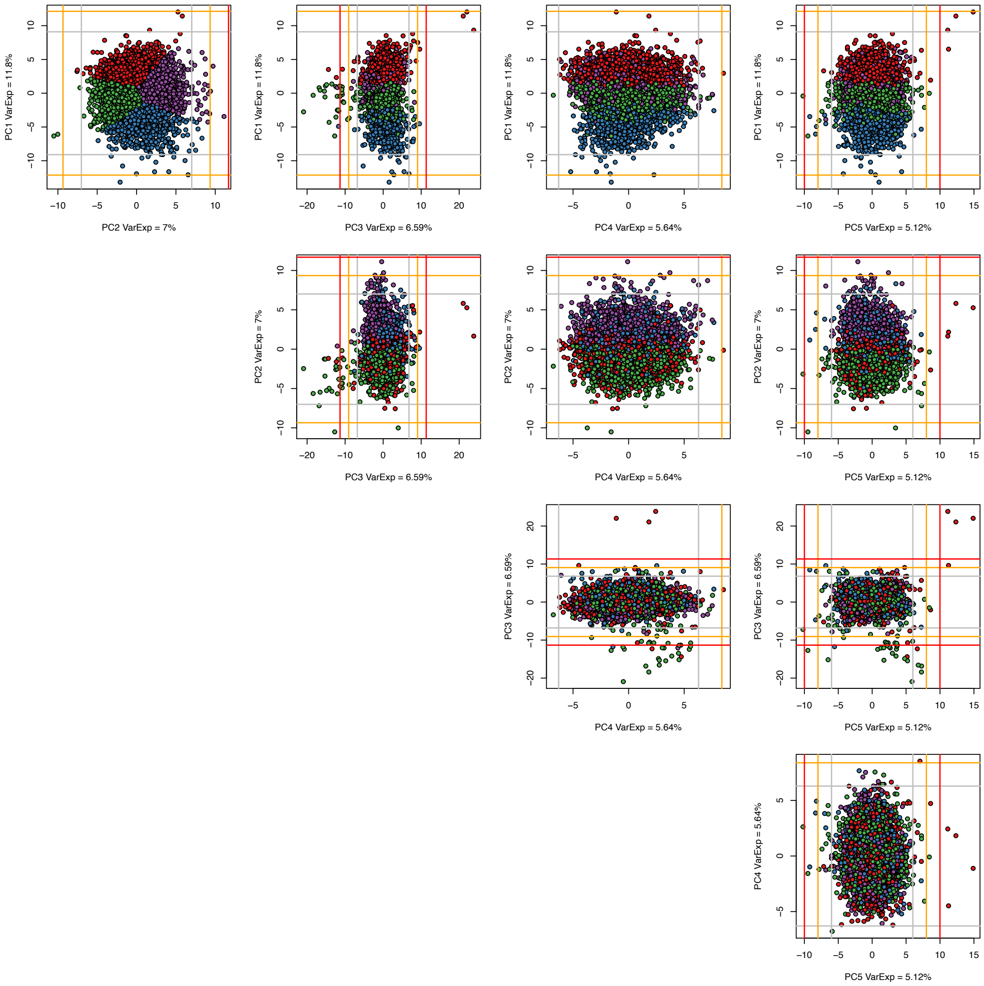

Figure Legend: A matrix plot of the top five principal components including demarcations of the 3rd (grey), 4th (orange), and 5th (red) standard deviations from the mean. Samples are color coded as in the summary PC plot above using a kmeans analysis of PC1 and PC2 with a k (number of clusters) set at 4. The choice of k = 4 was not robustly chosen it was a choice of simplicity to help aid visualize variation and sample mobility across the PCs.

2.5 Feature Distributions

2.5.1 Estimates of normality: W-statistics for raw and log transformed data

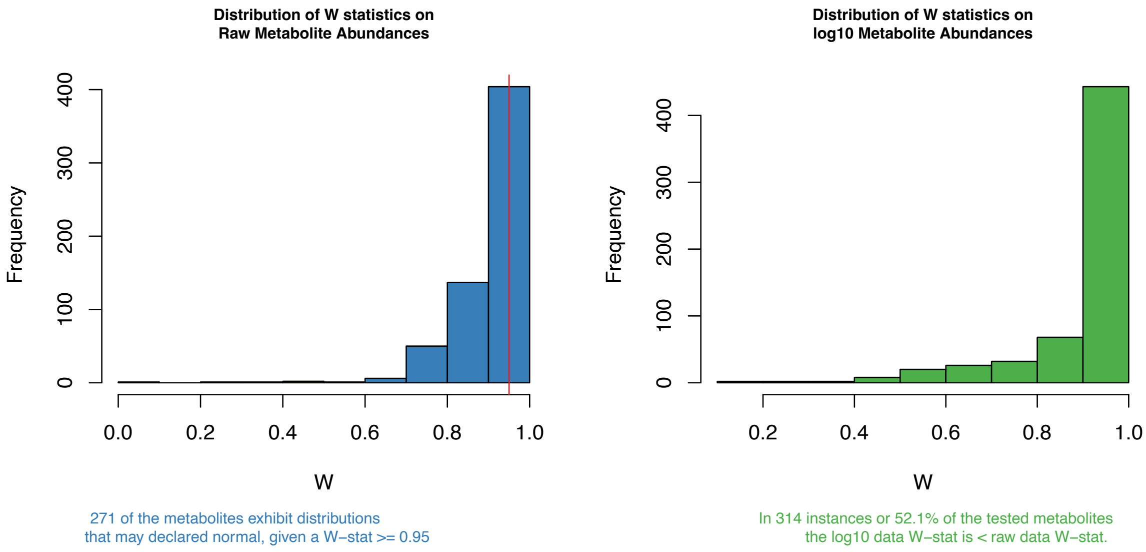

Figure Legend: Histogram plots of Shapiro W-statistics for raw (left) and log transformed (right) data distributions. A W-statistic value of 1 indicates the sample distribution is perfectly normal and value of 0 indicates it is perfectly uniform. Please note that log transformation of the data *may not* improve the normality of your data.

Analysis details: Of the 603 features in the data 0 features were excluded from this analysis because of no variation or too few observations ($n < 40$). Of the remaining 603 metabolite features, a total of 271 may be considered normally distributed given a Shapiro W-statistic >= 0.95.

2.5.2 Distributions

A pdf report is being written to NEO_outlier_detection_pre_filtering.pdf that contains dotplot, histogram and distribution summary statistics for each metabolite in your data set, providing an opportunity to visually inspect all your metabolites feature data distributions.

2.6 Outliers

Evaluation of the number of samples and features that are outliers across the data.

percentile	outlying.features.by.sample	outlying.samples.by.feature
0%	0	0
25%	0	3
50%	0	8
75%	0	16
100%	135	52

Table Legend: The table reports the number of point estimates for the minimum (0%) median (50%) and maximum (100%) number of outlying features across samples and the number of outlying samples across features.

2.6.1 Notes on outlying samples at each metabolite|feature

There may be extreme outlying observations at individual metabolites|features that have not been accounted for. You may want to:

1. Turn these observations into NAs.
2. Winsorize the data to some maximum value.
3. Rank normalize the data which will place those outliers into the top of the ranked standard normal distribution.
4. Turn these observations into NAs and then impute them along with other missing data in your data set.

---

3 Influence of batch variables on filtered data

3.1 Filtered data *feature* missingness: influenced by possible explanatory variables

Feature missingness may be influenced by the metabolites (or features) biology or pathway classification, or your technologies methodology. The figure(s) below provides an illustrative evaluation of the proportion of *feature missigness* as a product of the variable(s) available in the raw data files.

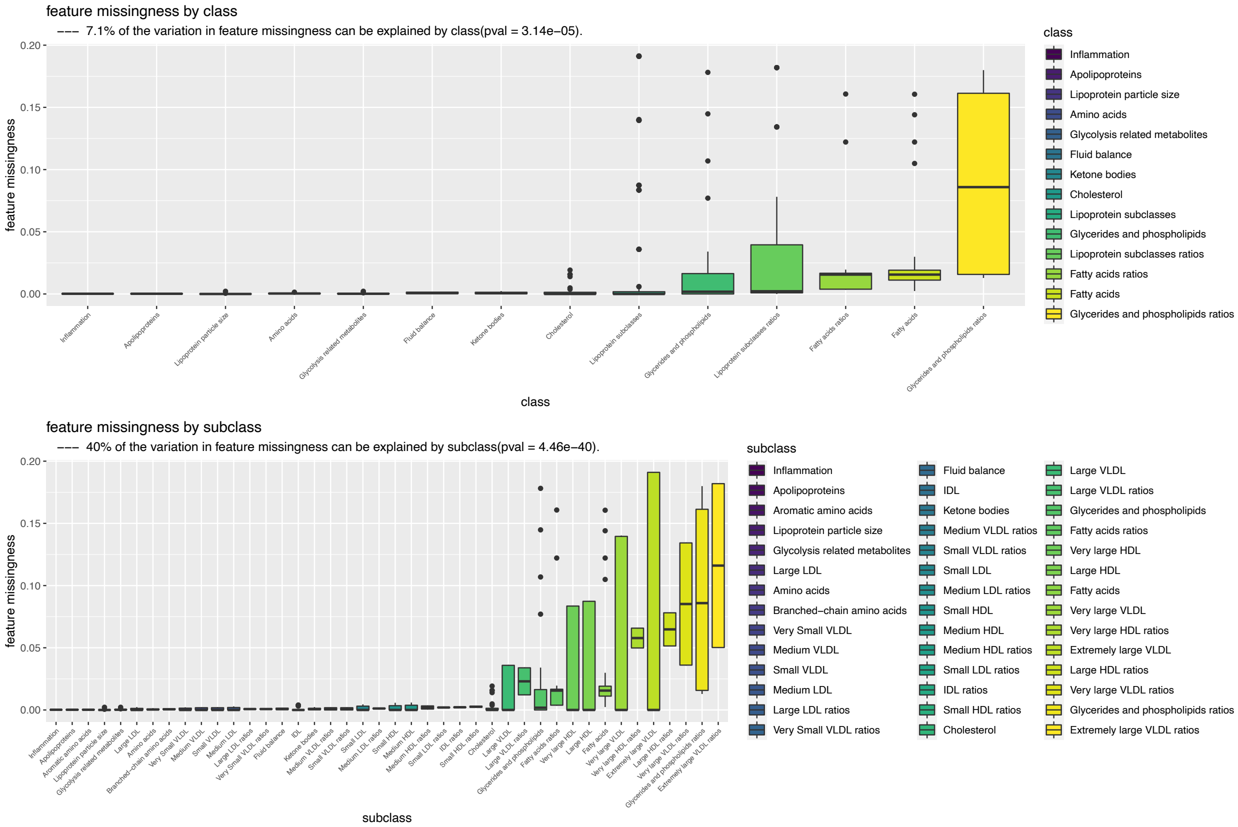

Figure Legend: Box plot illustration(s) of the relationship that available batch and biological variables have with feature missingness.

3.2 Filtered data *sample* missingness: influenced by possible explanatory variables

The figure provides an illustrative evaluation of the proportion of *sample missigness* as a product of sample batch variables provided by your supplier. This is the univariate influence of batch effects on *sample missingness*.

```
## [1] " -- No sample level batch variables were provided or all were invariable -- "
```

Figure Legend: Box plot illustration(s) of the relationship that available batch variables have with sample missingness.

3.3 Multivariate evaluation: batch variables

```
## [1] " -- No sample level batch variables were provided or all were invariable -- "
```

Table Legend: TypeII ANOVA: the eta-squared (eta-sq) estimates are an estimation of the percent of variation explained by each independent variable, after accounting for all other variables, as derived from the sum of squares. This is a multivariate evaluation of batch variables on *sample missingness*.

4 Sample Total Peak|Abundance Area (TPA):

Total peak|abundance area (TPA) is simply the sum of the abundances measured across all features. TPA is one measure that can be used to identify unusual samples given their entire profile. However, the level of missingness in a sample may influence TPA. To account for this we:

1. Evaluate the correlation between TPA estimates across all features with TPA measured using only those features with complete data (no missingness).

2. Determine if the batch effects have a measurable impact on TPA.

4.1 Relationship with missingness

Correlation between total peak area (at complete features) and missingness

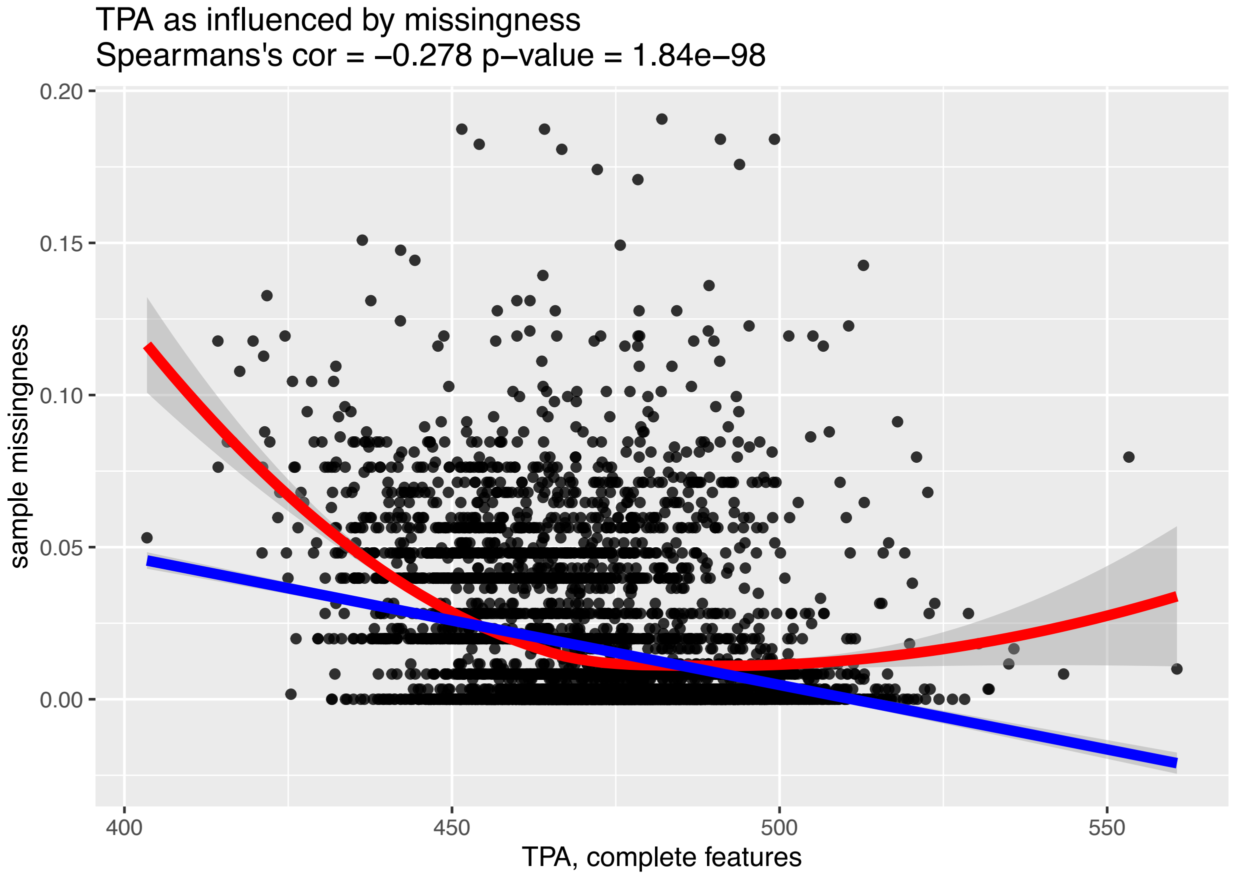

Figure Legend: Relationship between total peak area at complete features (x-axis) and sample missingness (y-axis).

4.2 Univariate evaluation: batch effects

The figure below provides an illustrative evaluation of the *total peak area* as a product of sample batch variables provided by your supplier.

```
## [1] " -- No sample level batch variables were provided or all were invariable -- "
```

Figure Legend: Violin plot illustration(s) of the relationship between total peak area (TPA) and sample batch variables that are available in your data.

4.2.1 Multivariate evaluation: batch variables

```
## [1] " -- No sample level batch variables were provided or all were invariable -- "
```

Table Legend: TypeII ANOVA: the eta-squared (eta-sq) estimates are an estimation on the percent of variation explained by each independent variable, after accounting for all other variables, as derived from the sum of squares. This is a multivariate evaluation of batch variables on *total peak|abundance area* at complete features.

---

5 Power analysis

Exploration for case/control and continuous outcome data using the filtered data set

Analytical power analysis for both continuous and imbalanced presence/absence correlation analysis.

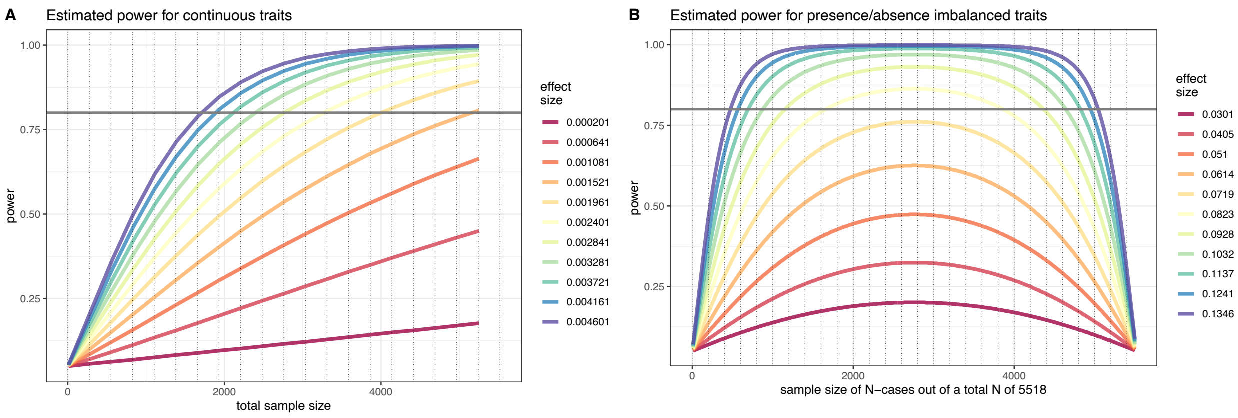

Figure Legend: Simulated effect sizes are illustrated by their color in each figure. Figure (A) provides estimates of power for continuous traits with the total sample size on the x-axis and the estimated power on the y-axis. Figure (B) provides estimates of power for presence/absence (or binary) traits in an imbalanced design. The estimated power is on the y-axix. The total sample size is set to 5518 and the x-axis depicts the number of individuals present (or absent) for the trait. The effects sizes illustrated here were chosen by running an initial set of simulations which identified effects sizes that would span a broad range of power estimates given the sample population's sample size.
